# Consistency in microbiomes in cultures of *Alexandrium* species isolated from brackish and marine waters

**DOI:** 10.1111/1758-2229.12736

**Published:** 2019-03-07

**Authors:** Eva Sörenson, Mireia Bertos‐Fortis, Hanna Farnelid, Anke Kremp, Karen Krüger, Elin Lindehoff, Catherine Legrand

**Affiliations:** ^1^ EEMiS, Department of Biology and Environmental Science, Linnaeus University Linnæus University Centre of Ecology and Evolution in Microbial Model Systems 39231, Kalmar Sweden; ^2^ Marine Research Centre Finnish Environment Institute P.O. Box 140, 00251, Helsinki Finland; ^3^ Leibniz Institute for Baltic Sea Research Warnemunde Seestrasse 15, 18119, Rostock Germany; ^4^ Max Planck Institute for Marine Microbiology Celsiusstraße 1, 28359, Bremen Germany

## Abstract

Phytoplankton and bacteria interactions have a significant role in aquatic ecosystem functioning. Associations can range from mutualistic to parasitic, shaping biogeochemical cycles and having a direct influence on phytoplankton growth. How variations in phenotype and sampling location, affect the phytoplankton microbiome is largely unknown. A high‐resolution characterization of the bacterial community in cultures of the dinoflagellate *Alexandrium* was performed on strains isolated from different geographical locations and at varying anthropogenic impact levels. Microbiomes of Baltic Sea Alexandrium ostenfeldii isolates were dominated by Betaproteobacteria and were consistent over phenotypic and genotypic *Alexandrium* strain variation, resulting in identification of an A. ostenfeldii core microbiome. Comparisons with *in situ* bacterial communities showed that taxa found in this A. ostenfeldii core were specifically associated to dinoflagellate dynamics in the Baltic Sea. Microbiomes of Alexandrium tamarense and *minutum,* isolated from the Mediterranean Sea, differed from those of A. ostenfeldii in bacterial diversity and composition but displayed high consistency, and a core set of bacterial taxa was identified. This indicates that *Alexandrium* isolates with diverse phenotypes host predictable, species‐specific, core microbiomes reflecting the abiotic conditions from which they were isolated. These findings enable in‐depth studies of potential interactions occurring between *Alexandrium* and specific bacterial taxa.

## Introduction

Interactions between phytoplankton and bacteria in aquatic environments are intricate and have large impacts on ecosystem functioning (Azam and Malfatti, 2007; Buchan *et al*., 2014). Specifically, phytoplankton bacteria interactions, ranging from mutualism (Amin *et al*., 2009) to resource competition (Risgaard‐Petersen *et al*., 2004), have effects on the whole ecosystem in terms of the amount of carbon that is recycled or stored (Cole, 1982; Jiao *et al*., 2010), the level of regenerated nitrogen (Eppley and Peterson, 1979) and utilization of captured energy (Buchan *et al*., 2014). The diversity, functioning and efficiency of bacteria associating with phytoplankton cells (the microbiome) are thought to be determined by the biochemical composition of phytoplankton and their released organic matter (Azam and Malfatti, 2007; Buchan *et al*., 2014; Seymour *et al*., 2017). The composition of the microbiome has been shown to be host specific in association with, for example, diatoms and dinoflagellates (Schäfer *et al*., 2002; Guannel *et al*., 2011; Lawson *et al*., 2017; Behringer *et al*., 2018).

Dinoflagellates release organic matter continuously during their life cycle (Villacorte *et al*., 2015) affecting both composition and function of their microbiome (Sarmento *et al*., 2013). The role of bacteria‐dinoflagellate interactions may include growth stimuli (Sakami *et al*., 1999; Ferrier *et al*., 2002; Bolch *et al*., 2011), life cycle cues (Adachi *et al*., 2003) and regulation of toxin production (Doucette and Powell, 1997; Gallacher and Smith, 1999). Lawson *et al*. (2017) showed the existence of a consistent core microbiome among dinoflagellate *Symbiodinium* clades, pointing at taxonomically consistent interactions with significance to dinoflagellate functions. Few studies have characterized these complex interactions (Hattenrath‐Lehmann and Gobler, 2017), which is a prerequisite for understanding consequences for large scale biogeochemical processes.

Dinoflagellates of the genus *Alexandrium* are reported from many geographical areas in the world's oceans (Anderson *et al*., 2012) and are known to release bioactive compounds, potent toxins (Anderson *et al*., 2012) and allelochemicals that are poorly chemically characterized (Ma *et al*., 2009), which can interact with microbial food web dynamics (Weissbach *et al*., 2011). *Alexandrium* populations display a large intraspecific phenotypic diversity, in terms of growth rate (Suikkanen *et al*., 2013; Brandenburg *et al*., 2018), toxin production (Tillmann *et al*., 2009; Suikkanen *et al*., 2013; Martens *et al*., 2017; Brandenburg *et al*., 2018), allelochemical activity (Hakanen *et al*., 2014; Brandenburg *et al*., 2018), nutritional strategies (Glibert and Legrand, 2006) and ability to bioluminesce (Valiadi *et al*., 2012). Due to this large phenotypic diversity *Alexandrium* is an ideal model organism for investigating the consistency of microbiomes over host specific, intraspecific or environmental variations (Tahvanainen *et al*., 2012; Suikkanen *et al*., 2013; Hakanen *et al*., 2014). We hypothesize that the high diversity among *Alexandrium* isolates would result in diverse, isolate specific, microbiomes in culture.

Several earlier studies have taxonomically characterized bacteria associated with marine *Alexandrium* in culture, identifying Proteobacteria [mainly Roseobacter (Alphaproteobacteria) and Alteromonas (Gammaproteobacteria) clades] and Bacteroidetes as dominating bacterial phyla (Kopp, 1997; Hold *et al*., 2001; Biegala *et al*., 2002; Jasti *et al*., 2005). However, these studies are limited to marine conditions (salinity ~31) and low sequencing resolution and coverage, which preclude conclusions of the microbiome consistency and specific associations of individual bacterial species and *Alexandrium* isolates. A recent study of an *Alexandrium* bloom at marine conditions, using 16S amplicon sequencing, found associations with Flavobacteriia (*Owenweeksia* and the NS5 marine group), Alpha‐ (Rhodobacterales) and Gammaprotebacteria (Alteromonadales) (Hattenrath‐Lehmann and Gobler, 2017), showing concordance, at phylum and order level, between findings in culture with those from the environment.

In this study, we define microbiome as consisting of the whole bacterial assemblage found in the *Alexandrium* cultures. Our aim was to explore how host specificity, intraspecies variations and sampling location influence the diversity and consistency of the microbiome of *A. ostenfeldii*, *A. minutum* and *A. tamarense* isolates, and investigate the existence of a species‐specific core microbiome. In total 28 strains were included, of which 20 belong to *A. ostenfeldii*, isolated from four distinct locations in the brackish Baltic Sea Proper. These strains have been shown to have phenotypic (Suikkanen *et al*., 2013; Hakanen *et al*., 2014) and genotypic (Tahvanainen *et al*., 2012) differences. The remaining strains belonged to *A. minutum* (*n* = 7) and *A. tamarense* (*n* = 1), isolated from eight locations in the northwest Mediterranean Sea (marine conditions) with different anthropogenic impact levels, classified using the Land Uses Simplified Index, LUSI (E. Flo, unpubl.). Two *in situ* datasets from the Baltic Sea were used to investigate if members from the identified core microbiome of *A. ostenfeldii* in culture were linked to natural dinoflagellate dynamics.

## Results and discussion

### *Identification of dinoflagellates and diversity of microbiomes*

The dinoflagellates were taxonomically identified by amplification and sequencing of their ITS‐regions which corroborated identifications previously made using microscopy (S. Fraga and I. Bravo, unpubl.; Kremp *et al*., 2009) except for the strain AL10C, which was previously identified as *A. minutum,* but showed a high level of identity (99%) to an *A. tamarense* strain (AJ005048.1; Supporting Information Fig. S1). The *Alexandrium* strains were cultured using sterile methods at conditions mimicking those of their respective origins, brackish (salinity 6.5 or 7) for *A. ostenfeldii* and marine (salinity 31) for *A. minutum/tamarense* (Supporting Information Table S1). Nucleic acid samples were obtained by filtering cultures (density of *A. ostenfeldii*: 0.03–0.15 × 10^5^ cells/ml and *A. minutum/tamarense*: 2.10–3.50 x 10^5^ cells/ml; Supporting Information Table S1) using 0.2 μm filters, capturing the whole bacterial assemblage of the cultures at late exponential growth phase. The microbiomes were characterized using high‐throughput 16S rRNA gene amplicon sequencing, which resulted in a total of 9 776 193 sequences and 3649 operational taxonomic units (OTUs; 97% similarity clustering; Supporting Information Table S2). In this first high resolution characterization of the associated bacterial community of multiple species of *Alexandrium*, large differences in richness and diversity of the microbiomes were observed between the species (Supporting Information Fig. S2). At the OTU‐level, the *A. ostenfeldii* microbiomes showed a higher level of richness [average chao1‐index: 1081, ± 252 (SD)] and a higher diversity [average alpha Shannon index: 4.22, ± 0.67 (SD)] compared to the *A. minutum/tamarense* richness [average chao1‐index: 481, ± 152 (SD)] and diversity [average alpha Shannon index: 3.15, ± 0.56 (SD)] (Supporting Information Fig. S2). The differences in richness and diversity between *A. ostenfeldii* and *A. minutum/tamarense* microbiomes might reflect the longer time the latter species were kept in culture, 2 years compared to 8–16 years respectively (Supporting Information Table S1).

### *Similarity of microbiome composition*

**Figure 1:**
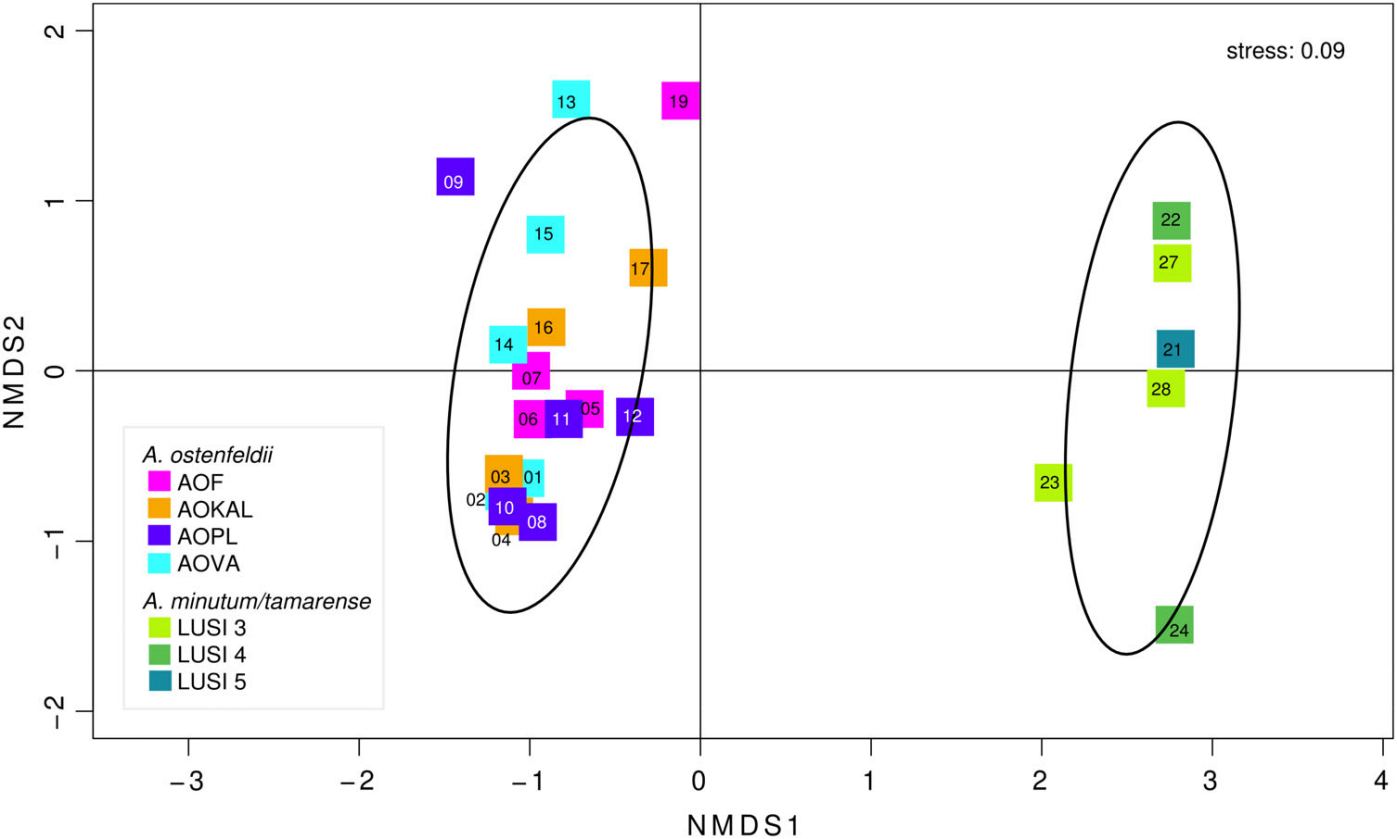
Dissimilarity analysis, nMDS, using a distance matrix with Bray Curtis dissimilarity of all strains, using R version 3.3.2, with packages Vegan (Oksanen *et al*., 2008) and Cluster (Maechler *et al*., 2016). Inherent stress: 0.09. Samples with > 162 000 read‐pairs were included in the analysis (excluding samples 18 (AOKAL), 20 (AOPL), 25 (LUSI1) and 26 (LUSI6), having < 32 000 read‐pairs; Supporting Information Table S2). The colours represent location of sampling: A. ostenfeldii – strains were isolated as resting cysts from four locations in the Baltic Sea Proper (AOF – Föglö Archipelago, Åland, AOKAL – Kalmar strait, Sweden, AOPL – Hel, Poland, AOVA – Valleviken, Sweden) (Tahvanainen *et al*., 2012); or level of anthropogenic impact (LUSI index): *A. minutum/tamarense* – strains were isolated from the north west Mediterranean Sea as vegetative cells, from locations with varying levels of anthropogenic impacts estimated using the LUSI index, 1–6 (E. Flo, unpubl.; Supporting Information Fig. S4); and sample ID for each strain (Supporting Information Fig. S4 and Table S2). The confidence limit was set at 0.80.

To study if the composition of the microbiomes associated with *Alexandrium* could be related to intraspecies variations of *Alexandrium* strains or specific conditions at the location of isolation an nMDS analysis, using Bray–Curtis dissimilarity, was performed. This analysis showed that there was a clear separation between *A. ostenfeldii* and the *A. minutum*/*tamarense* strains regarding frequently occurring taxa (Fig. 1 and Supporting Information Fig. S3). The *A. ostenfeldii* microbiomes were very homogeneous (permAnova *R* ^2^ = 0.13, *P* value: 0.73; Fig. 1) considering that the strains were isolated from four geographically distant locations of the Baltic Sea and the inherent intraspecific variations previously demonstrated for *A. ostenfeldii* (Kremp *et al*., 2016; Martens *et al*., 2017; Supporting Information Fig. S4). Consequently, our results indicate that intraspecific variations or sampling location in the Baltic Sea did not have a significant impact on the composition of the microbiomes. These findings indicate that the interactions that occur between the dinoflagellate and their microbiomes, are consistent within a species‐specific functional range of *Alexandrium*, disregarding phenotypic variations of individual dinoflagellate strains. In the nMDS, the *A. minutum/tamarense* associated microbiomes were dispersed with a spread similar to that of the *A. ostenfeldii* microbiomes (Fig. 1), and they did not cluster according to the anthropogenic impact level at each location (estimated using LUSI; E. Flo, unpubl.; Supporting Information Fig. S4). The results indicate a high microbiome similarity also across the *A. minutum/tamarense* strains. Taken together, our results show that both the environment at the sampling location, brackish or marine, and the species of *Alexandrium*, had important impacts on the composition of the microbiomes.

### *Microbiome of A. ostenfeldii isolates*

**Figure 2:**
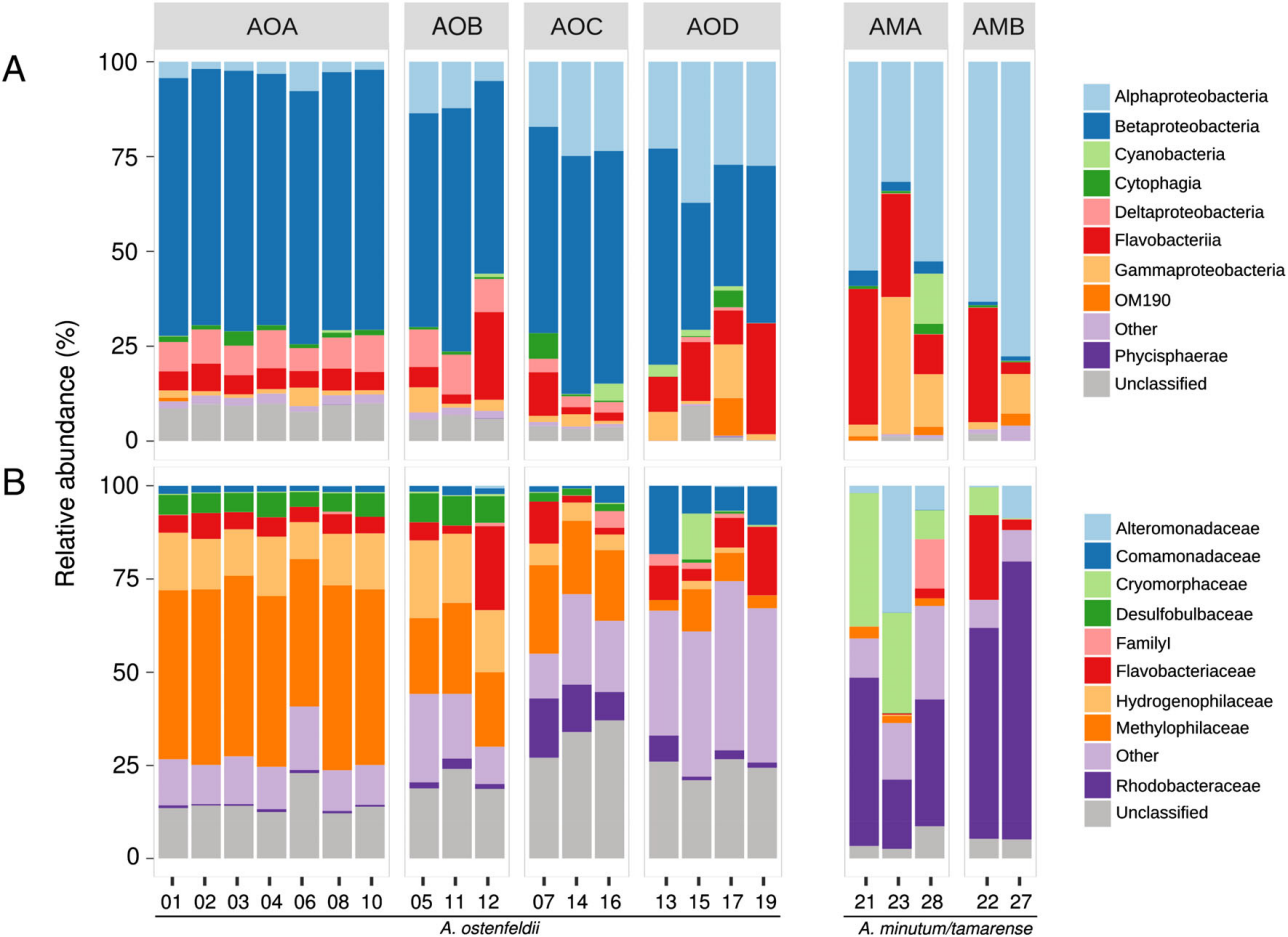
Composition of microbiomes of *Alexandrium* isolates at (A) Class and (B) Family level. Samples are grouped by dissimilarity (Supporting Information Fig. S3); AOA‐D: A. ostenfeldii group A–D, AMA‐B: *A. minutum/tamarense* group A–B. The cultures were harvested during late exponential phase using 0.2 μm, 47 mm filters and DNA was extracted (Boström *et al*., 2004). The V3‐V4 region of the 16S rRNA gene was amplified using primers 341F (CCTACGGGNGGCWGCAG) and 805R (GACTACHVGGGTATCTAATCC) and sequenced using Illumina MiSeq (Herlemann *et al*., 2011; Hugerth *et al*., 2014). The read‐pairs were clustered into OTUs using Usearch v8.1 (radius 1.5) (Edgar, 2013) corresponding to ~97% sequence identity and singletons were removed (Supporting Information Table S2). OTUs were classified using SILVA db 123 SSURef NR99; (Quast *et al*., 2013) using SINA v. 1.2.13 (Pruesse *et al*., 2012). The graph was constructed using ggplot2 (v. 2.2.1) (Wickham, 2009). The numbers for each sample specify the ID of each strain given in Supporting Information Fig. S4. The sequence data have been submitted to the European Nucleotide Archive (ENA) database under accession numbers ERS1617530‐ERS1617557.

The microbiomes of the *A. ostenfeldii* isolates were highly similar, forming four clusters (based on Bray–Curtis dissimilarity) of 3–7 samples each, originating from different locations (Supporting Information Fig. S3). These microbiomes included sequences affiliated with a broad range of bacterial classes: Beta‐ (58% of total sequences), Alpha‐ (14% of total sequences), Gamma‐ (3% of total sequences) and Deltaproteobacteria (6% of total sequences), Flavobacteriia (9% of total sequences) and Cytophagia (1.5% of total sequences). Represented, at family level, by *Methylophilaceae* (28% of total sequences), *Hydrogenophilaceae* (10% of total sequences), *Flavobacteriaceae* (7% of total sequences), *Comamonadaceae* (4% of total sequences), *Desulfobulbaceae* (4% of total sequences) and *Rhodobacteraceae* (3% of total sequences; Fig. 2). The high frequency of Betaproteobacteria in association with *A. ostenfeldii,* may be a reflection of the *in situ* brackish bacterial community composition of the Baltic Sea (Herlemann *et al*., 2011), which hosts more Betaproteobacteria compared to environments with higher salinity. The betaproteobacterial sequences, were primarily affiliated with *Methylophilaceae, Burkholderiaceae/Limnobacter* and *Comamonadaceae* (Supporting Information Fig. S5). Although Betaproteobacteria are not frequently found to dominate bacterial communities associated with phytoplankton, both *Methylophilaceae* and *Limnobacter* have previously been found in association with diatoms and coccolithophores (Amin *et al*., 2012; Green *et al*., 2015). Members of the family *Methylophilaceae*, are known to produce the growth promoting hormone indole‐3‐acetic acid (IAA) in association with plants (Doronina *et al*., 2014). Bacterial IAA has been observed to be exchanged for tryptophan in diatom cultures (Amin *et al*., 2015). Thus, a similar interaction may also occur between *A. ostenfeldii* and *Methylophilaceae. Methylophilaceae* prefer C1‐compounds, methanol or methylamine, as a source of carbon (Doronina *et al*., 2002) and they are known to degrade dimethylsulfide (DMS) (Eyice *et al*., 2015). The consistency of the dominating groups of the microbiome suggest that associations between these groups and *A. ostenfeldii* may be of importance for dinoflagellate metabolism, at brackish conditions, and are not an artefact of culturing.

### *Microbiome of A. minutum and A. tamarense isolates*

The microbiomes of *A. minutum/tamarense* isolates were clustered by similarity into two groups, of 2 and 3 samples, respectively, mixing samples from different environmental conditions (estimated using LUSI; E. Flo, unpubl.; Supporting Information Figs S3 and S4). These microbiomes were dominated by sequences affiliated with Alphaproteobacteria (56% of total sequences), Flavobacteriia (21% of total sequences) and Gammaproteobacteria (13% of total sequences). At family‐level these were represented by *Rhodobacteraceae* (46% of total sequences), *Cryomorphaceae* (16% of total sequences), *Alteromonadaceae* (10% of total sequences) and *Flavobacteriaceae* (6% of total sequences; Fig. 2). The microbiomes were highly similar to what has previously been reported from cultures of marine *Alexandrium* species including bacterial taxa such as *Rhodobacteraceae*, *Alteromonadaceae/Marinobacter* and *Cryomophaceae* (Sala *et al*., 2005; Jasti *et al*., 2005). Flavobacteriia as a group are known as degraders of complex organic carbon (Kirchman, 2002), and are commonly found in close association to phytoplankton cells in culture (Grossart *et al*., 2005). Alphaproteobacteria belonging to the Roseobacter group can be found both attached and free‐living and exhibit a broad potential for nutrient uptake (Grossart *et al*., 2005; Moran *et al*., 2007). Gammaproteobacteria are associated with phytoplankton bloom conditions, with specialized populations responding to phytoplankton decay (Teeling *et al*., 2012). Taken together the results show that the microbiomes of and *A. tamarense* strains included in the study are representative of *Alexandrium* cultured at marine conditions (salinity 31).

### Alexandrium *core microbiome*

Core microbiomes were calculated for *A. ostenfeldii* (Supporting Information Fig. S6) and *A. minutum/tamarense* (Supporting Information Fig. S7) strains, respectively, according to Lawson *et al*. (2017). The *A. ostenfeldii* core microbiome, contained five OTUs present in all 20 samples with an abundance > 0.0001%, affiliated to *Limnobacter* and *Hoeflea* (Supporting Information Fig. S6 and Table S3A). Within each group (Supporting Information Fig. S3), additional ubiquitous OTUs were identified (50), among which all four groups had OTUs that were affiliated to *Hydrogenophaga*, *Methylothenera*, *Flavobacteriaceae* or *Comamonadaceae* (Supporting Information Table S4A). These OTUs, though not identical, had the same taxonomical affiliations and were therefore considered to belong to the core *A. ostenfeldii* microbiome, in total 55 OTUs. For the *A. minutum/tamarense* isolates, 13 OTUs were present in all samples (excluding samples 25 and 26 with < 162 000 reads) (Supporting Information Fig. S7 and Table S3B). These were affiliated to *Limnobacter, Pyruvatibacter, Tepidamorphus, Hoeflea, Hyphomonas, Marivita, Marinobacter, Methylophaga* and *Salinispirillum*. Within the two groups of strains (Supporting Information Fig. S3), additional OTUs with the same taxonomical annotations were identified (39), which were affiliated to *Taesokella, Fabibacter, Sphingopyxis, Spongibacter, Salinirepens* and *Ahrensia* (Supporting Information Table S4B). Together, these 52 OTUs were considered the core microbiome of *A. minutum/tamarense*. The consistency of the core microbiomes indicates that associations between *Alexandrium* and bacteria in culture are stable and predictable. These findings enable further identification and characterization of specific *Alexandrium*‐bacterial interactions.

### *Culture conditions*

There are several considerations that should be made before drawing conclusions about *in situ* associations from *in vitro* conditions (Sala *et al*., 2005; Garcés *et al*., 2007; Hattenrath‐Lehmann and Gobler, 2017; Behringer *et al*., 2018). For example, culture conditions and time in culture can have significant impacts as culturing can alter the bacterial community composition within hours (Sapp *et al*., 2007; Weissbach *et al*., 2010). To investigate the effect of long term culturing on the associated bacterial communities, *A. ostenfeldii* (strains 13–20) and *A. minutum/tamarense* (strains 21–28) isolates were monitored after 6 and 4 months of culturing respectively. Similar to studies of diatoms in culture (Schäfer *et al*., 2002; Behringer *et al*., 2018), the bacterial communities of the *Alexandrium* isolates is this study were shown to be consistent over time (Supporting Information Table S1 and Fig. S8). However, in the high‐resolution sequencing data, shifts in the relative abundance among some of the most frequent betaproteobacterial OTUs were observed in *A. ostenfeldii* strains 1–12, compared to strains 13–20 (Supporting Information Fig. S5). Strains 13–20 had previously been kept at the same conditions as 1–12, but were moved to another lab and sampled after 6–9 weeks of exposure to slightly different conditions with regards to light, salinity and temperature (Supporting Information Table S1). Taken together these results show that, given stable conditions, the members of the core microbiome of *Alexandrium* are consistent, but shifts in the relative abundance of the microbiome, including members of the core, can be expected as a reflection of changed environmental conditions.

### *Connections with* in situ *dinoflagellate populations in the Baltic Sea*

**Figure 3:**
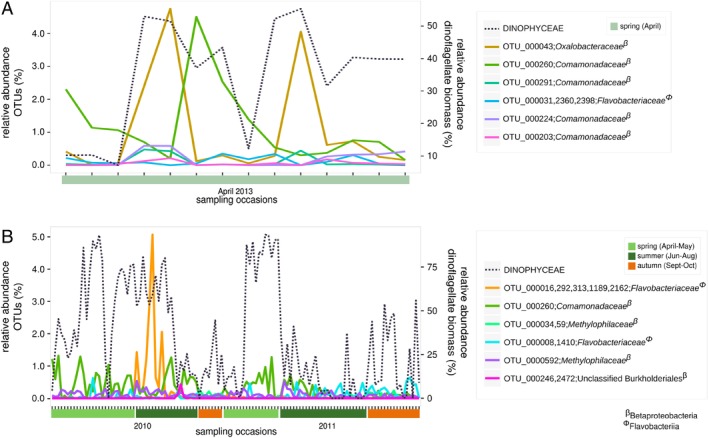
Dynamics of OTUs with a relative abundance > 0.2% and a similarity of ≥ 95% to members from the core microbiome of *A. ostenfeldii,* in (A) a naturally occurring spring bloom, Prodiversa, 2013 (Bunse *et al*., 2016; Godhe *et al*., 2016) and (B) a time series covering April–October in a coastal to off‐shore transect, Planfish, 2010–2011 (Legrand *et al*., 2015), both from the Baltic Sea Proper. Relative abundance (%) of OTUs on the left *y*‐axis, the dinoflagellate biomass dynamics from the corresponding datasets on the right *y*‐axis (%). The legend lists Planfish/Prodiversa‐OTUs named by corresponding OUTs from the A. ostenfeldii dataset, including their taxonomic affiliation at family level. The relative abundances of dinoflagellate biomass were calculated based on the carbon content of dinoflagellates per total phytoplankton carbon, including the species: *Dinobryon* spp, *Dinophysis* sp*, Gymnodiniales* spp, *Gymnodinium* spp, *Gyrodinium* spp, *Heterocapsa rotundata*, Katodinium glaucum, *Peridinella catenata, Peridinella* (single cell), *Protoperidinium* spp and *Scrippsiella*. Plots were made using ggplot2 (v. 2.2.1) (Wickham, 2009) in R 3.4.0.

In contrast to the Mediterranean Sea isolates, which were isolated as vegetative cells, the *A. ostenfeldii* isolates were isolated as cysts (Tahvanainen *et al*., 2012). To investigate if OTUs from the *A. ostenfeldii* core microbiome were present in the pelagic zone, and if their frequency could be related to natural dinoflagellate dynamics, two Baltic Sea microbial datasets were explored (Fig. 3A). The Prodiversa cruise took place during April 2013, representing a transect of the Baltic Sea Proper, capturing a mixed dinoflagellate spring bloom (Bunse *et al*., 2016; Godhe *et al*., 2016). The Planfish dataset was sampled during the productive season (April–October) of 2010–2011, covering a smaller geographic area west of the island of Gotland, capturing both spring and summer blooms of different dinoflagellate species (Legrand *et al*., 2015) (Fig. 3B). OTUs corresponding to members of the *A. ostenfeldii* core microbiome (OTU_000043 and OTU_000260), were found to follow the biomass dynamics of the dinoflagellate spring bloom, captured by the Prodiversa cruise (Fig. 3A). These OTUs were affiliated to Betaproteobacteria (*Oxalobacteriaceae* and *Comamonadaceae*; Supporting Information Table S4A). In the Planfish dataset, one of the same core OTUs (OTU_000260), increased in relative abundance during spring and summer dinoflagellate blooms (Fig. 3B). These results indicate that members of the core microbiome of *A. ostenfeldii* in culture followed *in situ* dinoflagellate occurrences, suggesting that they may be closely associated with Baltic Sea dinoflagellates, regardless of species.

## Conclusions

This study, the first high resolution molecular characterization of the microbiome of *A. ostenfeldii* isolates, showed that neither intraspecific variations nor the location of sampling in the brackish Baltic Sea Proper had a significant impact on the isolate microbiome composition. Similarly, the level of anthropogenic impact at the different sites of isolation of *A. minutum/tamarense* strains could not be linked to the composition of the different microbiomes. Instead, the microbiomes were highly consistent among strains of *A. ostenfeldii* and *A. minutum/tamarense,* respectively, and core microbiomes were identified. These core microbiomes were likely shaped by abiotic conditions, like salinity, light and temperature as shifts in relative abundances could be seen upon changed culture conditions, and selected by the respective dinoflagellate host in a species‐specific manner. The identification of an *Alexandrium* core microbiome makes this model system ideal for in‐depth studies of specific interactions likely occurring between the phytoplankton and key bacterial taxa – principal for understanding the impact of interactions on the ecosystem. In future studies, core microbiomes could be identified from natural phytoplankton blooms using culture independent methods such as single cell genomics.

## Conflict of interest

The authors declare no conflict of interest.

## Supporting information


**Appendix S1:** Supporting Information

**Table S3A** – Core microbiome of Alexandrium ostenfeldii, with an OTU‐abundance > 0.0001% (Lawson *et al*. 2017) . Specified by the Class and Family/Genus of the closest relative in GenBank (as given in Table S4A), the number of OTUs per Family and the relative abundance of those OTUs per group. Groups are specified in Fig. S4; AO – all A. ostenfeldii strains; AOA – strains 1–4, 6, 8, 10; AOB – strains 5, 11, 12; AOC – strains 7, 14, 16; AOD – strains 13, 15, 17, 19. Note that samples 18 and 20 are excluded (< 162 000 reads) and samples 9 is considered and outgroup of the A. ostenfeldii cluster (Fig. S4) and are therefore not included in any group.
**Table S3B** – Core microbiome of *Alexandrium minutum/tamarenes*, with an OTU‐abundance > 0.0001% (Lawson *et al*. 2017). Specified by the Class and Family/Genus of the closest relative in GenBank (as given in table S4B), the number of OTUs per Family and the relative abundance of those OTUs per group. Groups are specified in Figure S4; AM – all *A. minutum/tamarense* strains (excluding samples 25, 26 (< 162 000 reads); AMA – strains 21, 23, 28; AMB – strains 22, 27. Note that strain 24 is considered and outgroup of the *A. minutum/tamarense* cluster in Figure S4 and therefore not included in any group.

**Table S4A** Core microbiome (55 OTUs) of Alexandrium ostenfeldii, specifying: OTU, accession number of closest relative in GenBank, % identity, genus and family of that strain and the sequence of the OTU.
**Table S4B**. Core microbiome (52 OTUs) of *Alexandrium minutum/tamarense*, specifying: OTU, accession number of closest relative in GenBank, % identity, genus and family of that strain and the sequence of the OTU.
