# Supplementary material for: Consistency in microbiomes in cultures of Alexandrium species isolated from brackish and marine waters
Source: Environ Microbiol Rep. 2019 Mar 7;11(3):425–33. doi: 10.1111/1758-2229.12736 (PMC6563467; doi:10.1111/1758-2229.12736)
Supplement: Supplementary file 1 — Appendix S1: Supporting Information [file EMI4-11-425-s001.docx]

Supplementary material Sörenson et al.

Figure S1


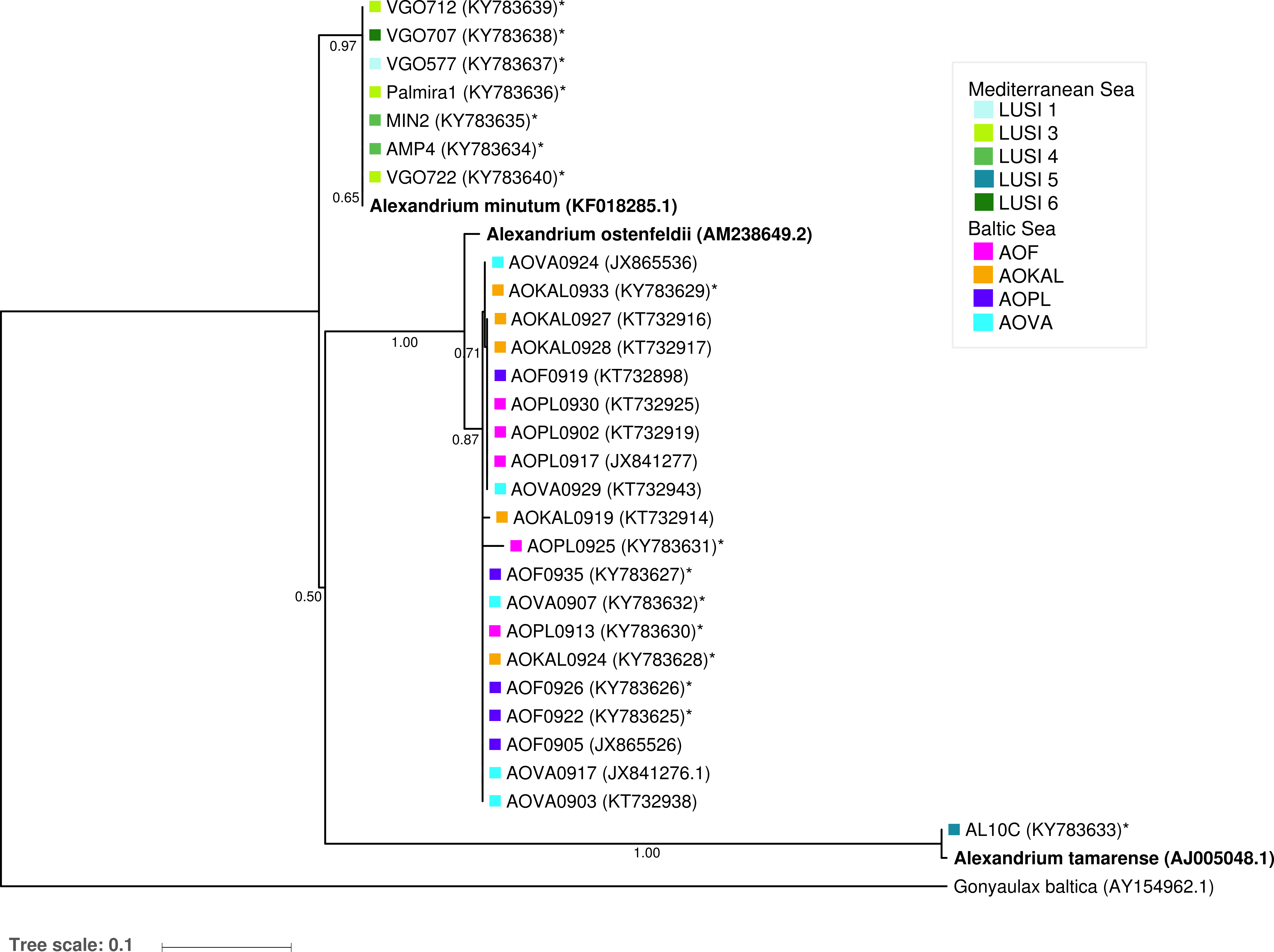


Figure S1 – Identification of the *Alexandrium* strains in this study (n = 28), using the ITS1, 5.8S rRNA and ITS2 region. The ITS region was amplified using the primers ITS1 (TCCGTAGGTGAACCTGCGG) and ITS4 (TCCTCGCTTATTGATATGC) (White *et al.*, 1990) followed by bi-directional sequencing of the 600 bp amplification product at Eurofins (Eurofins, Ebersberg, Germany). The rooted Maximum-Likelihood tree was made using PhyML 3.0, “A la carte” mode at Phylogeny.fr (Dereeper *et al.*, 2008) with 100 bootstraps, and includes sequences aligned using MAFFT v7.130b with the L-INS-I, local pairwise alignment option (Katoh *et al.*, 2005). Samples marked with * were sequenced during this study, unmarked samples are from previous studies (Tahvanainen *et al.*, 2012; Le Tortorec *et al.*, 2016). AOF – Föglö, Åland, AOKAL – Kalmar, Sweden, AOPL – Hel, Poland, AOVA – Valleviken, Sweden, (followed by 09XX; Table S1); LUSI 1: VGO577– La Fosca beach; LUSI 3: Palmira1 – Palmira beach, VGO712 – Vilanova harbor and VGO722 – Cambrils harbor; LUSI 4: MIN2 – Arenys harbor and AMP4 – Palma harbor; LUSI 5: AL10C – Estartit (*A. tamarense* strain); LUSI 6: VGO707 – Alfacs bay. The Land Uses Simplified Index (LUSI) was used to assess the level of anthropogenic impact, by measuring chl-*a* as a proxy for the level of eutrophication by continental nutrient load, scale 0.75-8.75 going from little to strong influence, from land into the sea (E. Flo, unpublished). Reference sequences of closest relatives are shown in bold. Accession numbers to all sequences are given in brackets. *Gonyaulax baltica* was included as an outgroup.

Figure S2


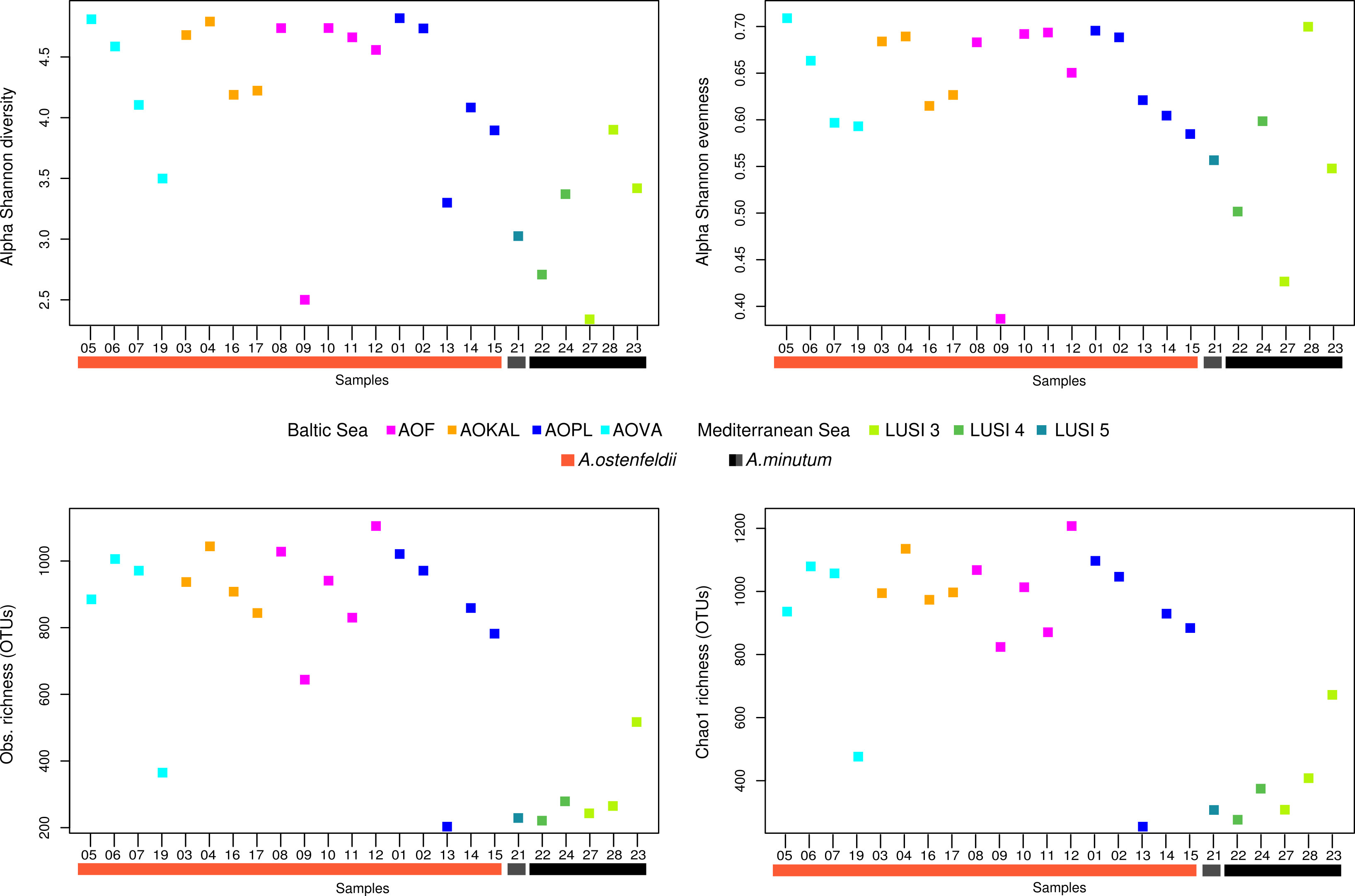


Figure S2 – Analysis of diversity and richness of *Alexandrium* microbiomes; Baltic Sea samples were grouped by location of sampling and the Mediterranean Sea samples according to classification by the LUSI index. The numbers for each sample specify the ID of each strain given in Figure S4. Samples were rarefied using Qiime (Caporaso *et al.*, 2010), to 162000 reads per sample, which excluded four samples (18 (AOKAL), 20 (AOPL), 25 (LUSI1), 26 (LUSI6)) OTUs with <10 reads were removed. The analyses were done in R, using the Vegan package (Oksanen *et al.*, 2008).

Figure S3


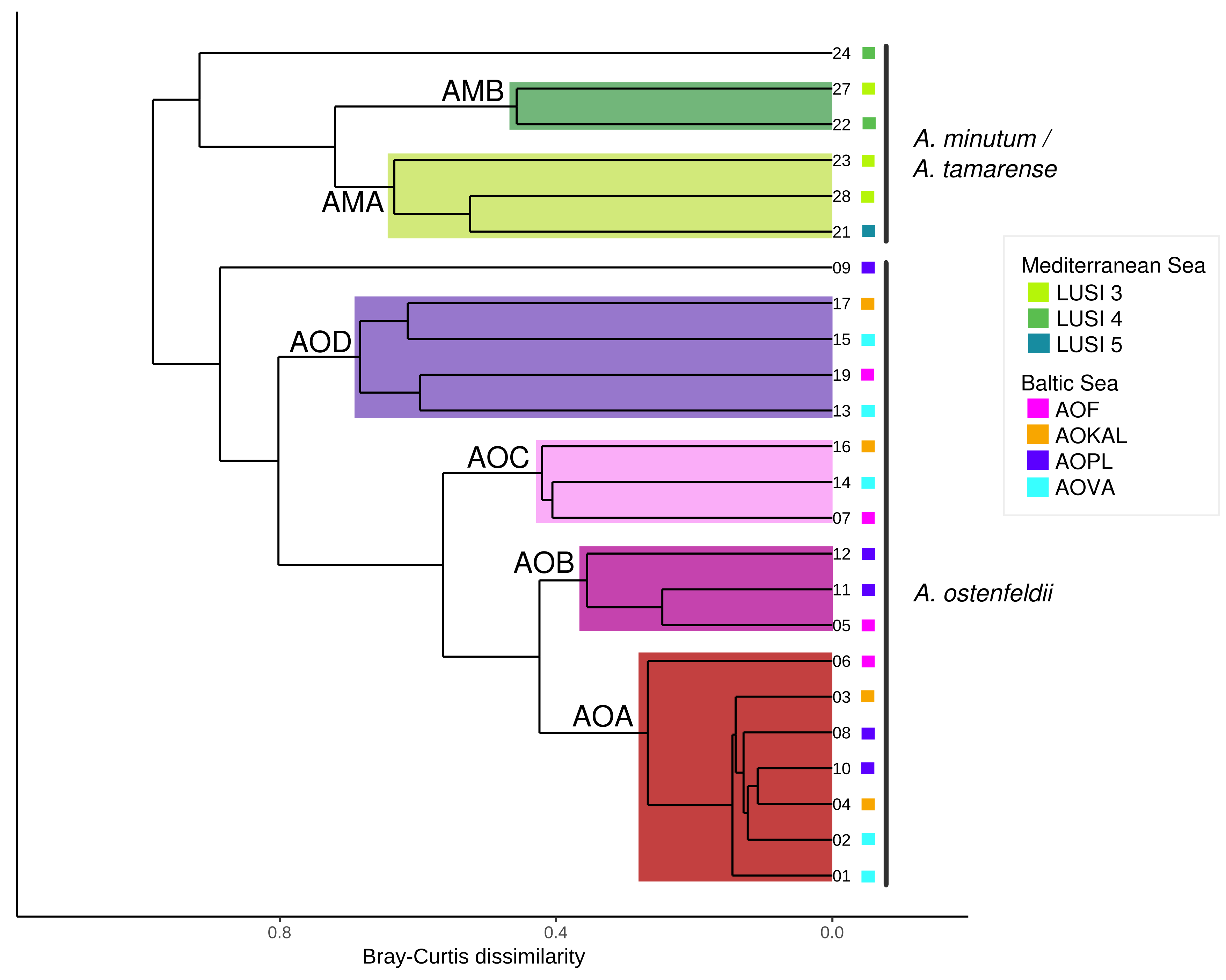


Figure S3 – Clusters of similar *Alexandrium* strains, later used to establish the core microbiome of the respective *Alexandrium* species (Figures S6 and S7, Tables S3 and S4). Bray-Curtis dissimilarity of *Alexandrium* strains was calculated on rarefied data, excluding samples with either too few reads (<162,000; 18 (AOKAL), 20 (AOPL), 25 (LUSI1), 26 (LUSI6))) or samples that are considered as outgroups (9 and 24). Clusters are indicated by colored rectangles and AOA-D (*A. ostenfeldii*, A-D) and AMA-B (*A. minutum/tamarense* A-B). All calculations and the plot were made using R 3.4.0 and packages Vegan (Oksanen *et al.*, 2008) and ggplot2 (Wickham, 2009).

Figure S4


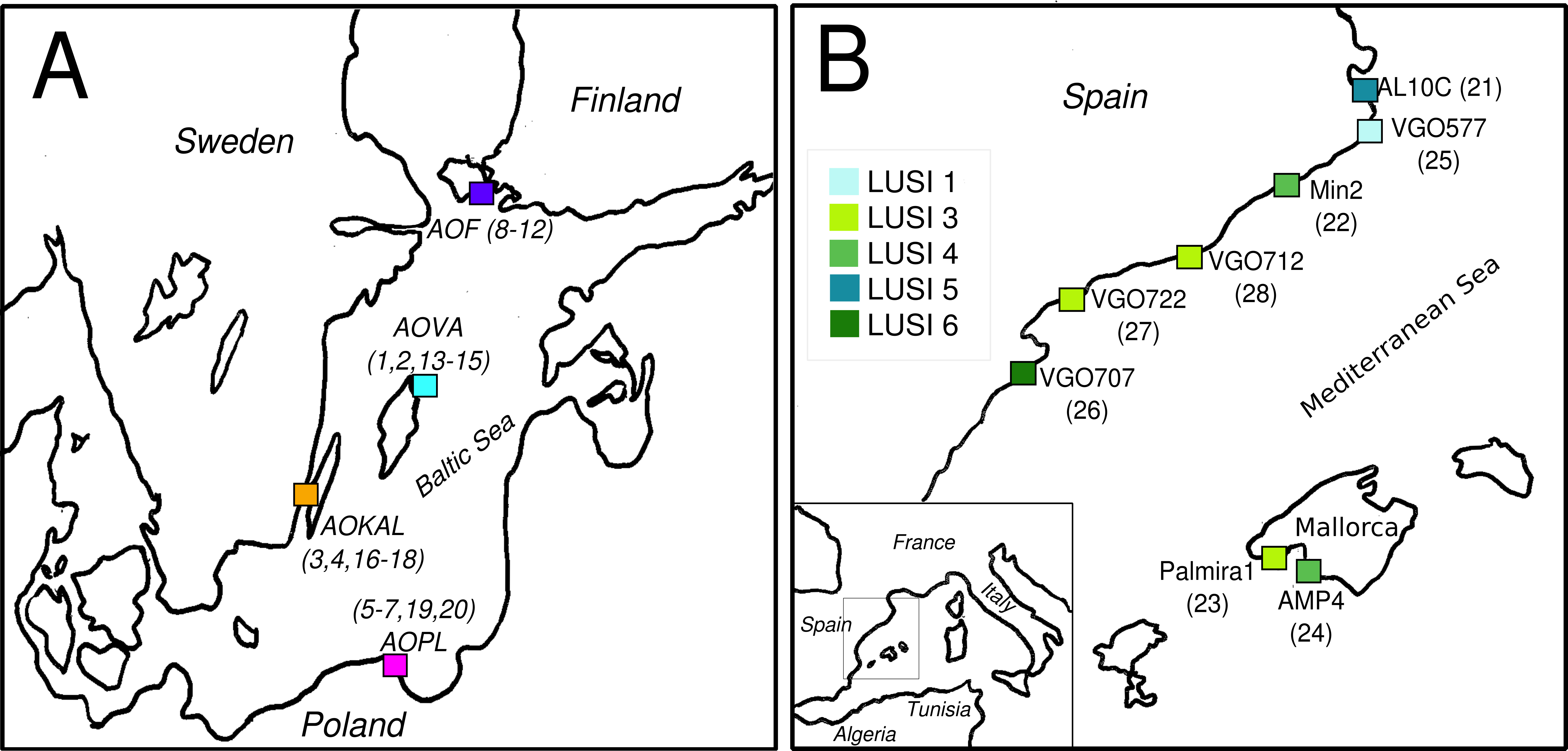


Figure S4 - Maps of the geographic location of sampling of the *Alexandrium* strains (numerical sample identifiers are indicated at each location within brackets); (A) *A. ostenfeldii* isolates from the Baltic Sea Proper (salinity 6.5 or 7); AOF – Föglö Archipelago, Åland, AOKAL – Kalmar Strait, Sweden, AOPL – Hel, Poland and AOVA – Valleviken, Sweden; (B) *A. minutum* isolates from the north west Mediterranean Sea (salinity 31), along the Catalán coast; the anthropogenic impact was assessed using the Land Uses Simplified Index (LUSI). This was done by measuring chl-*a* as a proxy for the level of eutrophication by continental nutrient load, scale 0.75-8.75 going from little to strong influence, from land into the sea (E. Flo, unpublished). LUSI 1: VGO577– La Fosca beach; LUSI 3: Palmira1 – Palmira beach, VGO712 – Vilanova harbor and VGO722 – Cambrils harbor; LUSI 4: MIN2 – Arenys harbor and AMP4 – Palma harbor; LUSI 5: AL10C – Estartit (*A. tamarense* strain); LUSI 6: VGO707 – Alfacs bay.

Figure S5


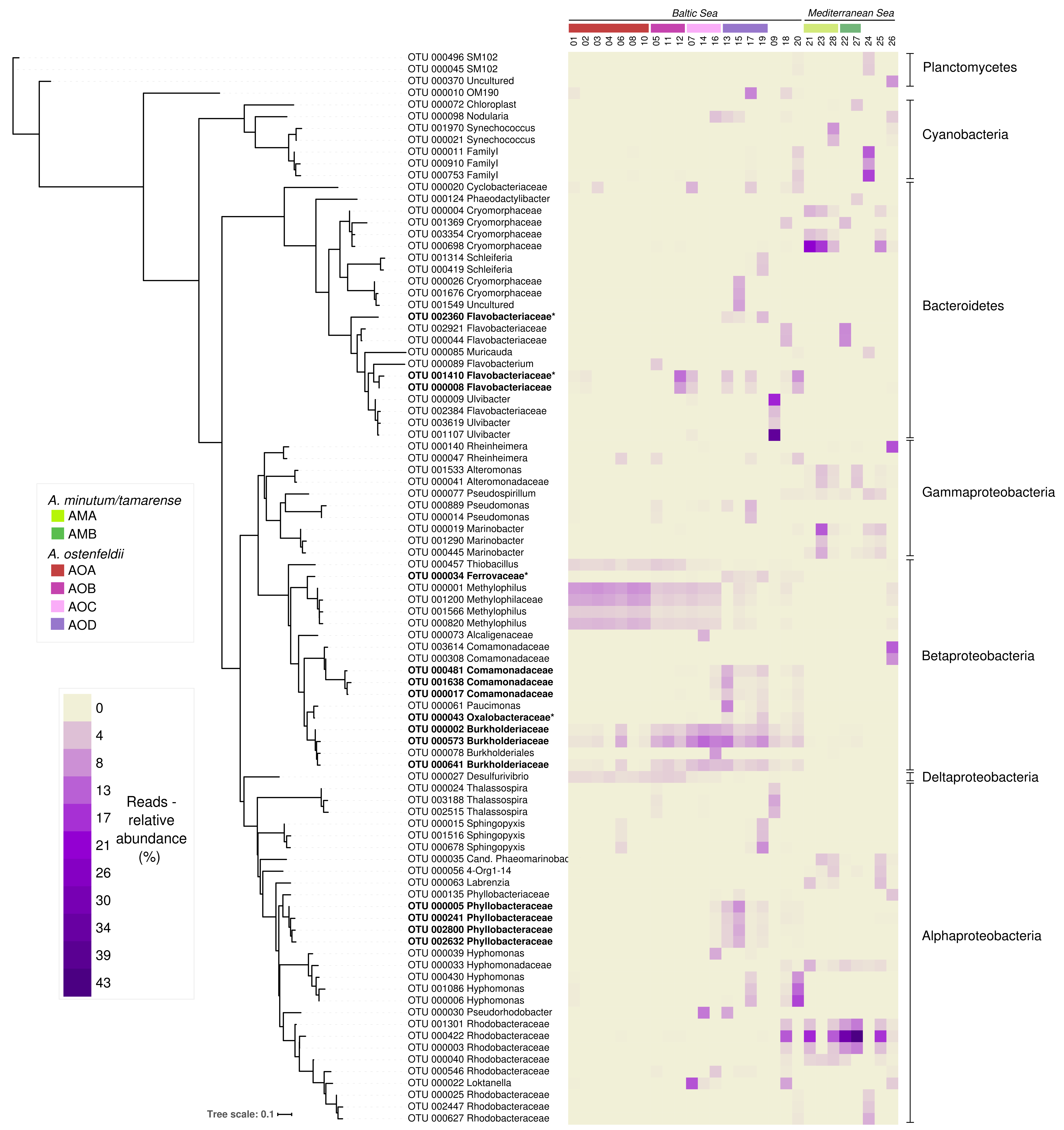


Figure S5 – Phylogenetic affiliation and relative abundance of the 91 OTUs with a relative abundance of ≥2% in one sample or more. OTUs are identified to the lowest taxonomic level (SILVA db 123 (SSURef NR99; (Quast *et al.*, 2013) using SINA v. 1.2.13 (Pruesse *et al.*, 2012). The number of reads were normalized to the total number of reads in each sample giving relative abundance. The heatmap was constructed in iTol (Letunic and Bork, 2016) with the OTUs distributed according to a Maximum-Likelihood tree (100 bootstraps) made using MAFFT (Katoh *et al.*, 2005). The bacterial clades are indicated to the right by class: Alpha-, Beta-, Delta- and Gammaproteobacteria, or phylum: Bacteroidetes, Cyanobacteria and Planctomycetes. A darker colour in the heatmap corresponds to a higher relative abundance. Heatmap columns are specified by sample identifiers and coloured according to the groups based on clusters of similarity (Figure S3). OTUs in bold belong to the *A. ostenfeldii* core microbiome (family level) and those marked with * were found to match OTUs from the Planfish (Legrand *et al.*, 2015) and Prodiversa (Bunse *et al.*, 2016) Baltic Sea natural community data sets (Table S4).

Figure S6


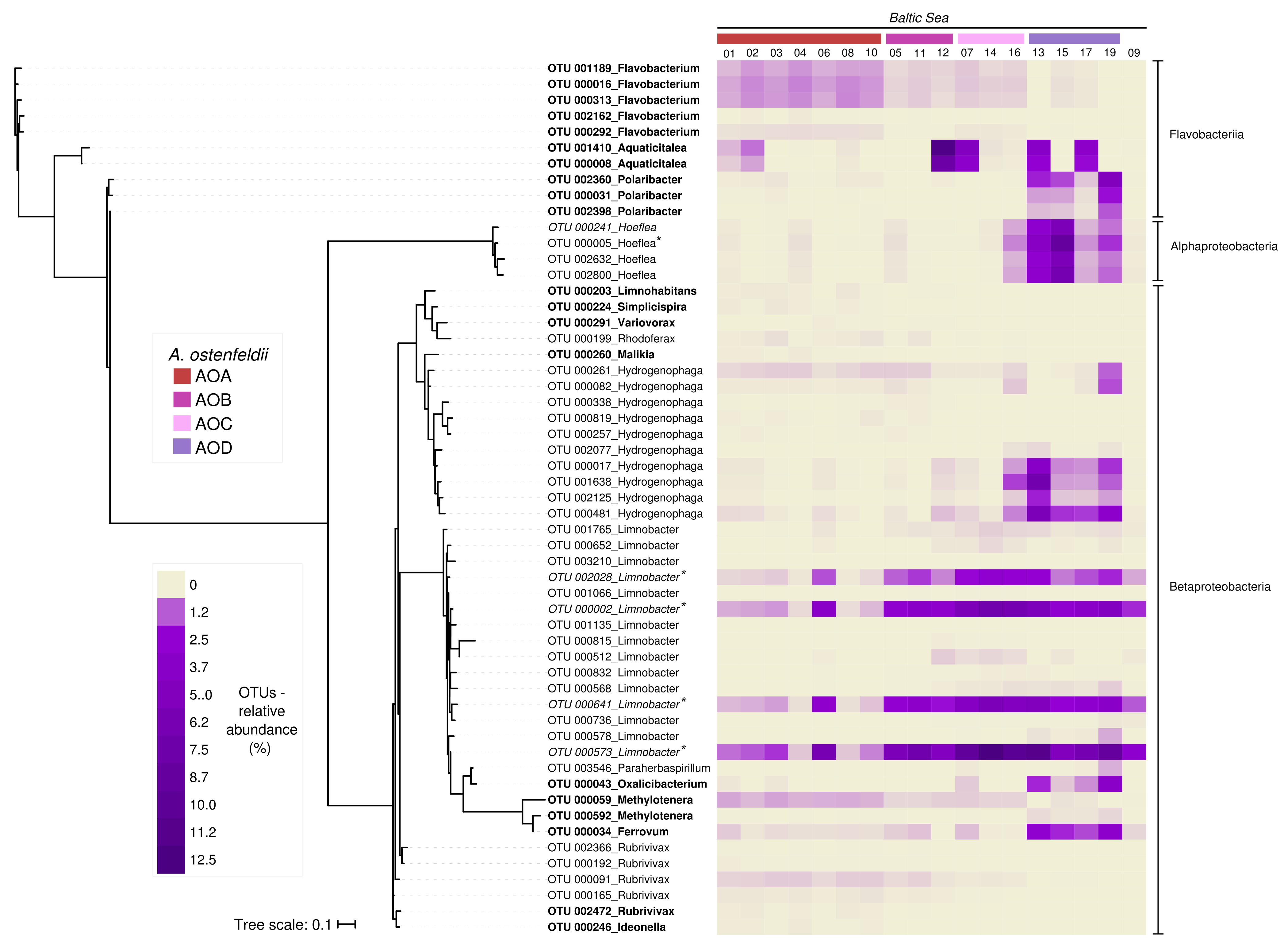


Figure S6 – Phylogenetic affiliation and relative abundance of *A. ostenfeldii* core microbiome OTUs with a read abundance >0.0001%. Samples are grouped according to identified clusters of similarity AOA-D (Figure S3) and sample 9 (outgroup). The number of reads were normalized to the total number of reads in each sample giving relative abundance. The heatmap was constructed in iTol (Letunic and Bork, 2016) with the OTUs distributed according to a Maximum-Likelihood tree (100 bootstraps) made using MAFFT (Katoh *et al.*, 2005). The bacterial clades are indicated to the right by class: Flavobacteriia, Alpha- and Betaproteobacteria. A darker colour in the heatmap corresponds to a higher relative abundance. OTUs with * are present in all samples, those in *italics* are also members of the *A. minutum/tamarense* core microbiome. OTUs are identified by the closest relative in GenBank (Table S4) at genus level. OTUs in **bold** were found to match OTUs from the Planfish (Legrand *et al.*, 2015) and Prodiversa (Bunse *et al.*, 2016) Baltic Sea natural community data sets (Table S4).

Figure S7


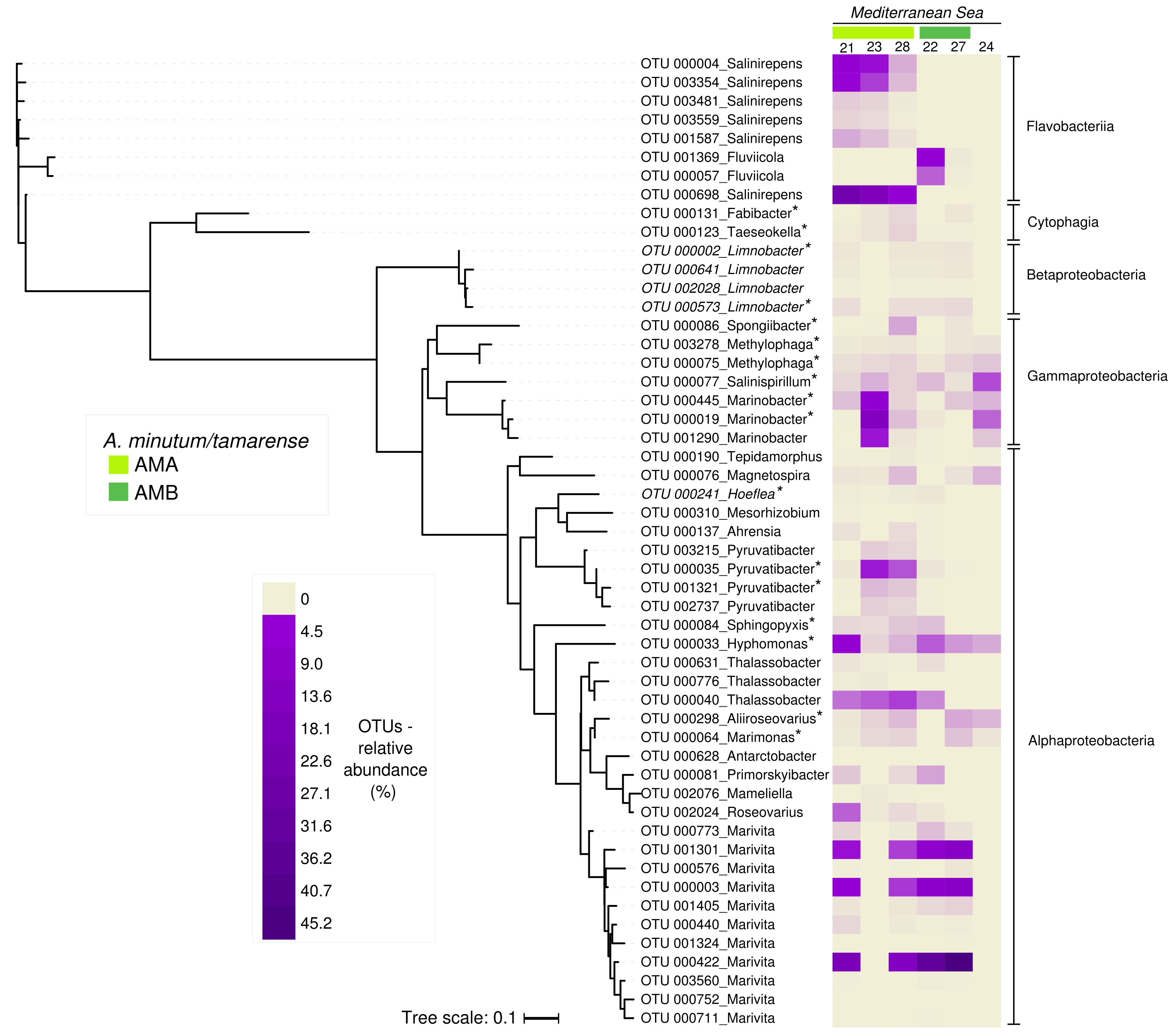


Figure S7 – Phylogenetic affiliation and relative abundance of *A. minutum/tamarense* core microbiome OTUs with a read abundance >0.0001%. Samples are grouped according to identified clusters of similarity AMA-B (Figure S3) and sample 24 (outgroup). The number of reads were normalized to the total number of reads in each sample giving relative abundance. The heatmap was constructed in iTol (Letunic and Bork, 2016) with the OTUs distributed according to a Maximum-Likelihood tree (100 bootstraps) made using MAFFT (Katoh *et al.*, 2005). The bacterial clades are indicated to the right by class: Flavobacteriia, Cytophagia, Alpha-, Beta- and Gammaproteobacteria. A darker colour in the heatmap corresponds to a higher relative abundance. OTUs with * are present in all samples, those in *italics* are also members of the *A. ostenfeldii* core microbiome. OTUs are identified by the closest relative in GenBank (Table S4) at genus level.

Figure S8


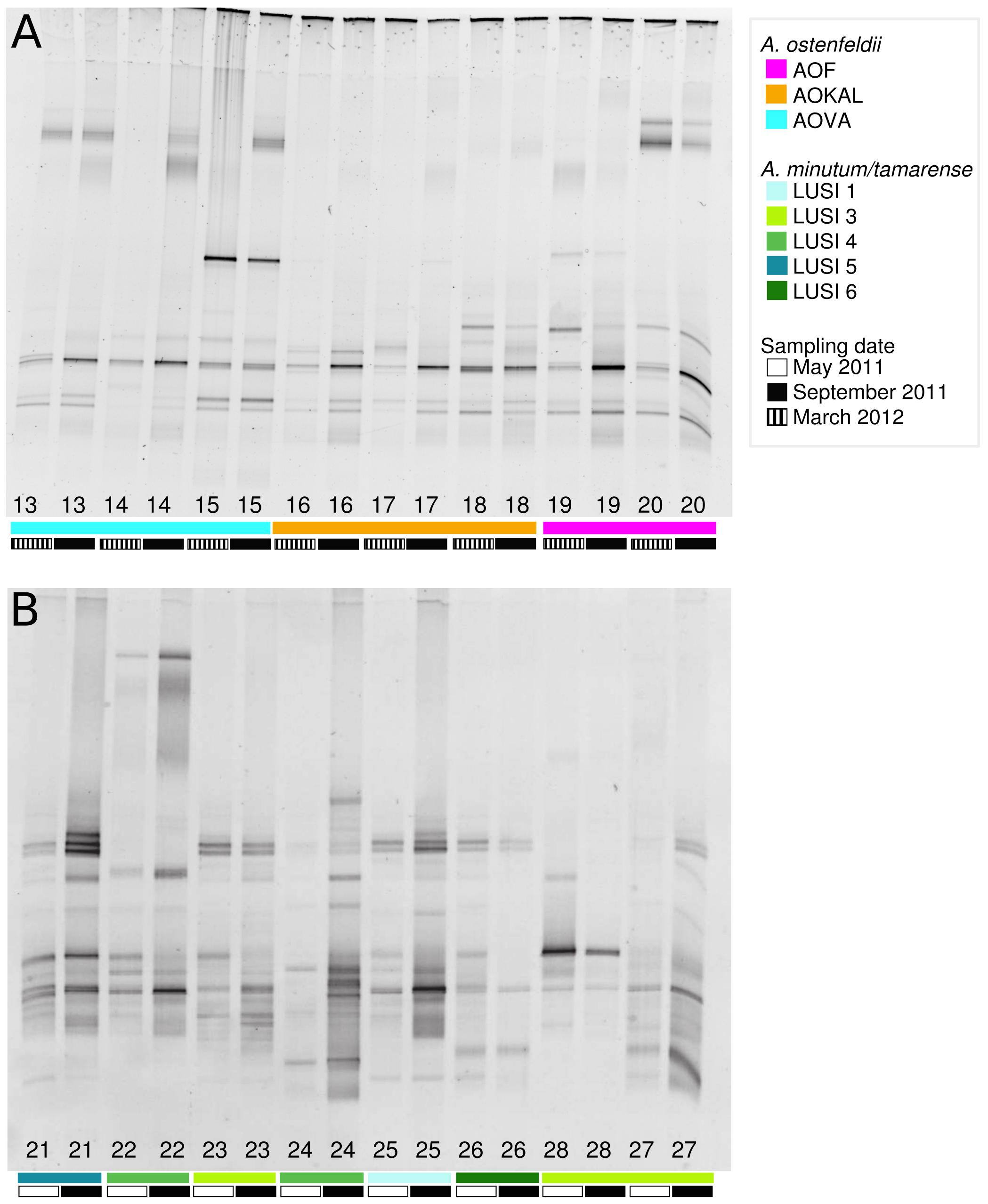


Figure S8 – DGGE gel of bacterial communities extracted from (A) *A. ostenfeldii* and (B) *A. minutum/tamarense* cultures, showing the consistency over time of the respective *Alexandrium* BCCs. The strains are specified by numerical identifiers (Table S1) and colored according to location of sampling (A) or level of anthropogenic impact (LUSI) (B). There are two replicates per strain (in total 16 samples per gel), extracted either 6 months apart (*A. ostenfeldii*), in September 2011 and March 2012, or 4 months apart (*A. minutum/tamarense*), May and September 2011.

Table S1

|  | ***A. ostenfeldii*** | | ***A. tamarense*** | ***A. minutum*** |
| --- | --- | --- | --- | --- |
| **Isolation area** | Baltic Sea | | Mediterranean Sea | |
| **Sample identifier** | **1-12** | **13-20** | **21** | **22-28** |
| **Isolated as** | cyst | cyst | vegetative cell | |
| **Time since isolation (years)** | 2 | 2  +  (6-9 weeks) | 9 | 8-16 |
| **Cell size (µm)** | 40-56 | | 22-51 | 15-30 |
| **Growth phase** | late exponential | | late exponential | |
| **Growth rate (day^-1^)** | 0.07-0.14 | | 0.15-0.28 | |
| **Concentration at harvest (cells ml^-1^)** | 0.03-0.15x10^5^ | | 2.10-3.50x10^5^ | |
| **Harvested volume (ml)** | 40 | | 40 | |
| **Medium** | F/2-Si | | L1 | |
| **Salinity** | 6.5 | 7 | 31 | |
| **Temperature (°C)** | 16 | 16.5 | 16.5 | |
| **Light:dark (h)** | 12:12 | 16:8 | 16:8 | |
| **Irradiance (μmol photons m^-2^s^-1^)** | 100 | 83 | 100 | |

Table S1 – Conditions under which the strains of *Alexandrium* were isolated, maintained in and harvested at. Sample identifiers specify each strain of *Alexandrium*. The growth phase was established by monitoring the cell density over time. The *A. ostenfeldii* were isolated in 2009 (Tahvanainen *et al.*, 2012) and the *A. minutum/tamarense* between 1995 and 2003 during blooms along the NW Mediterranean coast.

Table S2

| **Sample identifier** | **Strain** | **Number of merged reads-pairs** | **Number of reads after QC** | **% discarded**  **reads** | **Number of reads**  **after removal of singletons** | **Number of OTUs**  **after removal of singletons** |
| --- | --- | --- | --- | --- | --- | --- |
| **1** | AOVA0903 | 279,642 | 276,873 | 0.01 | 210,678 | 1,129 |
| **2** | AOVA0929 | 492,794 | 487,070 | 0.01 | 366,378 | 1,175 |
| **3** | AOKAL0919 | 403,069 | 399,007 | 0.01 | 307,763 | 1,077 |
| **4** | AOKAL0933 | 396,196 | 391,200 | 0.01 | 287,736 | 1,182 |
| **5** | AOF0905 | 295,150 | 291,719 | 0.01 | 230,920 | 991 |
| **6** | AOF0919 | 405,056 | 399,594 | 0.01 | 292,473 | 1,224 |
| **7** | AOF0926 | 497,854 | 490,987 | 0.01 | 354,245 | 1,135 |
| **8** | AOPL0925 | 415,605 | 411,235 | 0.01 | 310,278 | 1,195 |
| **9** | AOPL0930 | 284,290 | 279,237 | 0.02 | 242,990 | 719 |
| **10** | AOPL0902 | 370,901 | 366,585 | 0.01 | 277,309 | 1,052 |
| **11** | AOPL0913 | 206,196 | 203,519 | 0.01 | 162,939 | 857 |
| **12** | AOPL0917 | 400,998 | 395,461 | 0.01 | 253,655 | 1,236 |
| **13** | AOVA0907 | 276,756 | 273,423 | 0.01 | 256,506 | 238 |
| **14** | AOVA0917 | 332,588 | 324,220 | 0.03 | 226,889 | 944 |
| **15** | AOVA0924 | 391,318 | 383,530 | 0.02 | 279,230 | 898 |
| **16** | AOKAL0924 | 372,940 | 365,249 | 0.02 | 254,882 | 1,026 |
| **17** | AOKAL0927 | 1,228,113 | 1,211,634 | 0.01 | 1,028,504 | 1,204 |
| **18** | AOKAL0928 | 1343 | 1300 | 0.03 | 1020 | 208 |
| **19** | AOF0922 | 417,311 | 412,442 | 0.01 | 298,791 | 460 |
| **20** | AOF0935 | 22,551 | 22,190 | 0.02 | 19,980 | 275 |
| **21** | AL10C | 342,482 | 336,469 | 0.02 | 272,486 | 254 |
| **22** | MIN2 | 369,266 | 361,240 | 0.02 | 265,400 | 259 |
| **23** | Palmira1 | 369,683 | 362,575 | 0.02 | 232,444 | 598 |
| **24** | AMP4 | 329,139 | 324,167 | 0.02 | 237,124 | 326 |
| **25** | VGO577 | 41,810 | 40,066 | 0.04 | 31,138 | 269 |
| **26** | VGO707 | 1343 | 1302 | 0.03 | 840 | 144 |
| **27** | VGO722 | 300,022 | 293,964 | 0.02 | 266,954 | 287 |
| **28** | VGO712 | 531,777 | 519,340 | 0.02 | 462,502 | 345 |

Table S2 – The sample identifiers and strains followed by the number of reads at different steps of the bioinformatic process from raw reads to OTUs.

**References:**

Bunse, C., Bertos-Fortis, M., Sassenhagen, I., Sildever, S., Sjöqvist, C., Godhe, A., et al. (2016) Spatio-temporal interdependence of bacteria and phytoplankton during a Baltic Sea spring bloom. *Front. Microbiol.* **7**: 1–10.

Caporaso, J.G., Kuczynski, J., Stombaugh, J., Bittinger, K., Bushman, F.D., Costello, E.K., et al. (2010) QIIME allows analysis of high-throughput community sequencing data. *Nat. Methods* **7**: 335–336.

Dereeper, A., Guignon, V., Blanc, G., Audic, S., Buffet, S., Chevenet, F., et al. (2008) Phylogeny.fr: robust phylogenetic analysis for the non-specialist. *Nucleic Acids Res.* **36**: W465–W469.

Katoh, K., Kuma, K.I., Toh, H., and Miyata, T. (2005) MAFFT version 5: Improvement in accuracy of multiple sequence alignment. *Nucleic Acids Res.* **33**: 511–518.

Legrand, C., Fridolfsson, E., Bertos-Fortis, M., Lindehoff, E., Larsson, P., Pinhassi, J., and Andersson, A. (2015) Interannual variability of phyto-bacterioplankton biomass and production in coastal and offshore waters of the Baltic Sea. *Ambio* **44**: 427–438.

Letunic, I. and Bork, P. (2016) Interactive tree of life (iTOL) v3: an online tool for the display and annotation of phylogenetic and other trees. *Nucleic Acids Res.* **44**: W242–W245.

Oksanen, J., Kindt, R., Legendre, P., O’Hara, B., Simpson, G.L., Solymos, P.M., et al. (2008) The vegan package. *Community Ecol. Packag.* 190.

Pruesse, E., Peplies, J., and Glöckner, F.O. (2012) SINA: Accurate high-throughput multiple sequence alignment of ribosomal RNA genes. *Bioinformatics* **28**: 1823–1829.

Quast, C., Pruesse, E., Yilmaz, P., Gerken, J., Schweer, T., Yarza, P., et al. (2013) The SILVA ribosomal RNA gene database project: Improved data processing and web-based tools. *Nucleic Acids Res.* **41**: 590–596.

Tahvanainen, P., Alpermann, T.J., Figueroa, R.I., John, U., Hakanen, P., Nagai, S., et al. (2012) Patterns of post-glacial genetic differentiation in marginal populations of a marine microalga. *PLoS One* **7**: e53602.

Le Tortorec, A.H., Tahvanainen, P., Kremp, A., and Simis, S.G.H. (2016) Diversity of luciferase sequences and bioluminescence production in Baltic Sea *Alexandrium ostenfeldii*. *Eur. J. Phycol.* **51**: 317–327.

White, T.J., Bruns, T., Lee, S., and Taylor, J.W. (1990) Amplification and direct sequencing of fungal ribosomal RNA genes for phylogenetics. In, Innis, M.A., Gelfand, D.H., Sninsky, J.J., and White, T.J. (eds), *PCR-protocols: A guide to methods and applications.* Academic press, Inc., New York, NY, pp. 315–322.

Wickham, H. (2009) ggplot2 Springer New York, New York, NY.
