# Supplementary material for: Consistency in microbiomes in cultures of Alexandrium species isolated from brackish and marine waters
Source: Environ Microbiol Rep. 2019 Mar 7;11(3):425–33. doi: 10.1111/1758-2229.12736 (PMC6563467; doi:10.1111/1758-2229.12736)
Supplement: Supplementary file 2 — Table S3A – Core microbiome of Alexandrium ostenfeldii, with an OTU‐abundance > 0.0001% (Lawson et al. 2017) . Specified by the Class and Family/Genus of the closest relative in GenBank (as given in Table S4A), the number of OTUs per Family and the relative abundance of those OTUs per group. Groups are specified in Fig. S4; AO – all A. ostenfeldii strains; AOA – strains 1–4, 6, 8, 10; AOB – strains 5, 11, 12; AOC – strains 7, 14, 16; AOD – strains 13, 15, 17, 19. Note that samples 18 and 20 are excluded (< 162 000 reads) and samples 9 is considered and outgroup of the A. ostenfeldii cluster (Fig. S4) and are therefore not included in any group. Table S3B – Core microbiome of Alexandrium minutum/tamarenes, with an OTU‐abundance > 0.0001% (Lawson et al. 2017). Specified by the Class and Family/Genus of the closest relative in GenBank (as given in table S4B), the number of OTUs per Family and the relative abundance of those OTUs per group. Groups are specified in Figure S4; AM – all A. minutum/tamarense strains (excluding samples 25, 26 (< 162 000 reads); AMA – strains 21, 23, 28; AMB – strains 22, 27. Note that strain 24 is considered and outgroup of the A. minutum/tamarense cluster in Figure S4 and therefore not included in any group. [file EMI4-11-425-s002.docx]

Table S3

| **A** |  | **AO** | | **AOA** | | **AOB** | | **AOC** | | **AOD** | |
| --- | --- | --- | --- | --- | --- | --- | --- | --- | --- | --- | --- |
| **Class** | **Family/Genus – GenBank** | **n** | **relab %** | **n** | **relab %** | **n** | **relab %** | **n** | **relab %** | **n** | **relab %** |
| Alphaproteobacteria | Phyllobacteriaceae/Hoeflea | 1 | 0.940 | 1 | 0.051 | 3 | 0.058 | 4 | 0.241 | 4 | 2.859 |
| Betaproteobacteria | Burkholderiaceae/Limnobacter thiooxidans | 4 | 2.880 | 5 | 0.744 | 10 | 1.262 | 15 | 1.648 | 13 | 1.466 |
| Betaproteobacteria | Comamonadaceae |  |  | 13 | 0.087 | 8 | 0.086 | 7 | 0.120 | 1 | 2.935 |
| Betaproteobacteria | Comamonadaceae/Hydrogenophaga |  |  | 2 | 0.045 | 2 | 0.033 | 3 | 0.322 | 4 | 1.254 |
| Betaproteobacteria | Methylophilaceae/Methylotenera |  |  | 3 | 0.255 | 1 | 0.245 | 2 | 0.226 | 2 | 1.166 |
| Flavobacteriia | Flavobacteriaceae |  |  | 6 | 0.399 | 4 | 0.199 | 7 | 0.504 | 3 | 1.165 |
|  | **Sum Core/Group (otus):** | 5 |  | 30 |  | 28 |  | 38 |  | 27 |  |
|  | **Total unique otus:** | 55 |  |  |  |  |  |  |  |  |  |

Table S3A – Core microbiome of *Alexandrium ostenfeldii*, with an OTU-abundance > 0.0001% (Lawson *et al.* 2017) . Specified by the Class and Family/Genus of the closest relative in GenBank (as given in table S4A), the number of OTUs per Family and the relative abundance of those OTUs per group. Groups are specified in Figure S4; AO – all *A. ostenfeldii* strains; AOA – strains 1-4, 6, 8, 10; AOB – strains 5, 11, 12; AOC – strains 7, 14, 16; AOD – strains 13, 15, 17, 19. Note that samples 18 & 20 are excluded (<162000 reads) and samples 9 is considered and outgroup of the *A. ostenfeldii* cluster (Figure S4) and are therefore not included in any group.

| **B** |  | **AM** | | **AMA** | | **AMB** | |
| --- | --- | --- | --- | --- | --- | --- | --- |
| **Class** | **Family/Genus – GenBank** | **n** | **Relab %** | **n** | **Relab %** | **n** | **Relab %** |
| Alphaproteobacteria | *Hyphomonadaceae/Hyphomonas jannaschiana* | 1 | 2.181 | 1 | 2.416 | 1 | 2.255 |
| Alphaproteobacteria | *Phyllobacteriaceae/Hoeflea phototrophica* | 1 | 0.102 | 1 | 0.114 | 1 | 0.116 |
| Alphaproteobacteria | *Rhodobacteraceae/Marivita roseacus* | 2 | 0.555 | 7 | 0.760 | 15 | 4.098 |
| Alphaproteobacteria | *Rhodobiaceae/Pyruvatibacter mobilis* | 2 | 0.802 | 4 | 0.974 | 2 | 0.098 |
| Alphaproteobacteria | *Rhodobiaceae/Tepidamorphus gemmatus* | 1 | 0.497 | 2 | 0.296 | 1 | 0.174 |
| Alphaproteobacteria | *Rhodobacteraceae/Ahrensia marina* |  |  | 1 | 0.046 | 1 | 0.044 |
| Alphaproteobacteria | *Sphingomonadacea/Sphingopyxis flavimaris* | 1 | 0.532 | 1 | 0.566 | 1 | 0.480 |
| Betaproteobacteria | *Burkholderiaceae/Limnobacter thiooxidans* | 2 | 0.220 | 2 | 0.205 | 4 | 0.237 |
| Cytophagia | *Cytophagaceae/Taeseokella kangwonensis* | 1 | 0.188 | 1 | 0.325 | 1 | 0.077 |
| Cytophagia | *Flammeovirgaceae/Fabibacter misakiensis* | 1 | 0.211 | 1 | 0.279 | 1 | 0.110 |
| Flavobacteriia | *Crocinitomicaceae/Salinirepens amamiensis* |  |  | 6 | 3.892 | 2 | 1.926 |
| Gammaproteobacteria | *Alteromonadaceae/Marinobacter adhaerens* | 2 | 2.303 | 3 | 3.014 | 2 | 0.292 |
| Gammaproteobacteria | *Piscirickettsiaceae/Methylophaga nitratireducenticrescens* | 2 | 0.348 | 2 | 0.332 | 2 | 0.264 |
| Gammaproteobacteria | *Saccharospirillaceae/Salinispirillum marinum* | 1 | 1.131 | 1 | 0.785 | 1 | 0.653 |
| Gammaproteobacteria | *Spongiibacteraceae/Spongiibacter marinus* | 1 | 0.350 | 1 | 0.498 | 1 | 0.129 |
|  | **Sum Core/Group (otus):** | 18 |  | 34 |  | 36 |  |
|  | **Total unique otus:** | 52 |  |  |  |  |  |

Table S3B – Core microbiome of *Alexandrium minutum/tamarenes*, with an OTU-abundance > 0.0001% (Lawson *et al.* 2017). Specified by the Class and Family/Genus of the closest relative in GenBank (as given in table S4B), the number of OTUs per Family and the relative abundance of those OTUs per group. Groups are specified in Figure S4; AM – all *A. minutum/tamarense* strains (excluding samples 25, 26 (<162000 reads); AMA – strains 21, 23, 28; AMB – strains 22, 27. Note that strain 24 is considered and outgroup of the *A. minutum/tamarense* cluster in Figure S4 and therefore not included in any group.

**References:**

Lawson CA, Raina JB, Kahlke T *et al.* Defining the core microbiome of the symbiotic dinoflagellate, Symbiodinium. *Environ Microbiol Rep* 2017;**10**:7–11.
