# Supplementary material for: Consistency in microbiomes in cultures of Alexandrium species isolated from brackish and marine waters
Source: Environ Microbiol Rep. 2019 Mar 7;11(3):425–33. doi: 10.1111/1758-2229.12736 (PMC6563467; doi:10.1111/1758-2229.12736)
Supplement: Supplementary file 3 — Table S4A Core microbiome (55 OTUs) of Alexandrium ostenfeldii, specifying: OTU, accession number of closest relative in GenBank, % identity, genus and family of that strain and the sequence of the OTU. Table S4B. Core microbiome (52 OTUs) of Alexandrium minutum/tamarense, specifying: OTU, accession number of closest relative in GenBank, % identity, genus and family of that strain and the sequence of the OTU. [file EMI4-11-425-s003.docx]

| ***A. ostenfeldii* Core OTUs** | **ID - GenBank** | **%** | **Genus GenBank** | **Family GenBank** | **Sequence** |
| --- | --- | --- | --- | --- | --- |
| OTU_000002 | NR_025421.1 | 98.92 | *Limnobacter thiooxidans* | Burkholderiaceae | CCTACGGGGGGCTGCAGTGGGGAATTTTGGACAATGGGGGAAACCCTGATCCAGCAATGCCGCGTGTGCGAAGAAGGCCTTCGGGTTGTAAAGCACTTTTGTCAGGGAAGAAATCCTTTGGGCTAATACCCTAGGGGGATGACGGTACCTGAAGAATAAGCACCGGCTAACTACGTGCCAGCAGCCGCGGTAATACGTAGGGTGCAAGCGTTAATCGGAATTACTGGGCGTAAAGCGTGCGCAGGCGGTTGTGTAAGACAGGTGTGAAATCCCCGGGCTTAACCTGGGAATTGCATTTGTGACTGCACGACTAGAGTGTGTCAGAGGGGGGTGGAATTCCACGTGTAGCAGTGAAATGCGTAGATATGTGGAGGAACACCAATGGCGAAGGCAGCCCCCTGGGATAACACTGACGCTCATGCACGAAAGCGTGGGGAGCAAACAGGATTAGATACCCCAGTAGTC |
| OTU_000512 | NR_025421.1 | 98.07 | *Limnobacter thiooxidans* | Burkholderiaceae | CCTACGGGGGGCTGCAGTGGGGAATTTTGGACAATGGGGGAAACCCTGATCCAGCAATGCCGCGTGTGCGAAGAAGGCCTTCGGGTTGTAAAGCACTTTTGTCAGGGAAGAAATCCTTTGGGCTAATACCCTAGGGGGATGACGGTACCTGAAGAATAAGCACCGGCTAACTACGTGCCAGCAGCCGCGGTAATACGTAGGGTGCAAGCGTTAATCGGAATTACTGGGCGTAAAGCGTGCGCAGGCGGTTGTGTAAGACAGGTGTGAAATCCCCGGGCTTAACCTGGGAATGGCATTTTGAACTGGCAGTCTAGAGTGTGTCAGAGGGGGGTGGAATTCCACGTGTAGCAGTGAAATGCGTAGATATGTGGAGGAACACCAATGGCGAAGGCAGCCCCCTGGGATAACACTGACGCTCATGCACGAAAGCGTGGGGAGCAAACAGGATTAGATACCCTGGTAGTC |
| OUT_000568 | NR_025421.1 | 98.28 | *Limnobacter thiooxidans* | Burkholderiaceae | CCTACGGGAGGCTGCAGTGGGGAATTTTGGACAATGGGGGAAACCCTGATCCAGCAATGCCGCGTGTGCGAAGAAGGCCTTCGGGTTGTAAAGCACTTTTGTCAGGGAAGAAATCCTTTGGGCTAATACCCTAGGGGGATGACGGTACCTGAAGAATAAGCACCGGCTAACTACGTGCCAGCAGCCGCGGTAATACGTAGGGTGCAAGCGTTAATCGGAATTACTGGGCGTAAAGCGTGCGCAGGCGGTTGTGTAAGACAGGTGTGAAATCCCCGGGCTTAACCTGGGAATTGCATTTGTGACTGCACGACTAGAGTGTGTCAGAGGGGGGTGGAATTCCACGTGTAGCAGTGAAATGCGTAGATATGTGGAGGAACACCAATGGCGAAGGCAGCCCCCTGGGCCTGCACTGACGCTCATGCACGAAAGCGTGGGGAGCAAACAGGATTAGATACCCGTGTAGTC |
| OTU_000573 | NR_025421.1 | 98.92 | *Limnobacter thiooxidans* | Burkholderiaceae | CCTACGGGTGGCAGCAGTGGGGAATTTTGGACAATGGGGGAAACCCTGATCCAGCAATGCCGCGTGTGCGAAGAAGGCCTTCGGGTTGTAAAGCACTTTTGTCAGGGAAGAAATCCTTTGGGCTAATACCCTAGGGGGATGACGGTACCTGAAGAATAAGCACCGGCTAACTACGTGCCAGCAGCCGCGGTAATACGTAGGGTGCAAGCGTTAATCGGAATTACTGGGCGTAAAGCGTGCGCAGGCGGTTGTGTAAGACAGGTGTGAAATCCCCGGGCTTAACCTGGGAATTGCATTTGTGACTGCACGACTAGAGTGTGTCAGAGGGGGGTAGAATTCCACGTGTAGCAGTGAAATGCGTAGATATGTGGAGGAATACCGATGGCGAAGGCAGCCCCCTGGGATAACACTGACGCTCATGCACGAAAGCGTGGGGAGCAAACAGGATTAGATACCCTGGTAGTC |
| OTU_000578 | NR_025421.1 | 98.28 | *Limnobacter thiooxidans* | Burkholderiaceae | CCTACGGGTGGCTGCAGTGGGGAATTTTGGACAATGGGGGAAACCCTGATCCAGCAATGCCGCGTGTGCGAAGAAGGCCTTCGGGTTGTAAAGCACTTTTGTCAGGGAAGAAATCCTTTGGGCTAATACCCTAGGGGGATGACGGTACCTGAAGAATAAGCACCGGCTAACTACGTGCCAGCAGCCGCGGTAATACGTAGGGTGCAAGCGTTAATCGGAATTACTGGGCGTAAAGCGTGCGCAGGCGGTTGTGCAAGACAGATGTGAAATCCCCGGGCTCAACCTGGGAATTGCATTTGTGACTGCACGGCTAGAGTGTGTCAGAGGGGGGTAGAATTCCACGTGTAGCAGTGAAATGCGTAGATATGTGGAGGAACACCAATGGCGAAGGCAGCCCCCTGGGATAACACTGACGCTCATGCACGAAAGCGTGGGGAGCAAACAGGATTAGATACCCTGGTAGTC |
| OTU_000641 | NR_025421.1 | 98.71 | *Limnobacter thiooxidans* | Burkholderiaceae | CCTACGGGAGGCAGCAGTGGGGAATTTTGGACAATGGGCGCAAGCCTGATCCAGCAATGCCGCGTGTGCGAAGAAGGCCTTCGGGTTGTAAAGCACTTTTGTCAGGGAAGAAATCCTTTGGGCTAATACCCTAGGGGGATGACGGTACCTGAAGAATAAGCACCGGCTAACTACGTGCCAGCAGCCGCGGTAATACGTAGGGTGCAAGCGTTAATCGGAATTACTGGGCGTAAAGCGTGCGCAGGCGGTTGTGTAAGACAGGTGTGAAATCCCCGGGCTTAACCTGGGAATTGCATTTGTGACTGCACGACTAGAGTGTGTCAGAGGGGGGTGGAATTCCACGTGTAGCAGTGAAATGCGTAGATATGTGGAGGAACACCAATGGCGAAGGCAGCCCCCTGGGATAACACTGACGCTCATGCACGAAAGCGTGGGGAGCAAACAGGATTAGATACCCGTGTAGTC |
| OTU_000652 | NR_025421.1 | 98.49 | *Limnobacter thiooxidans* | Burkholderiaceae | CCTACGGGTGGCAGCAGTGGGGAATTTTGGACAATGGGGGAAACCCTGATCCAGCCATTCCGCGTGAGTGAAGAAGGCCTTCGGGTTGTAAAGCACTTTTGTCAGGGAAGAAATCCTTTGGGCTAATACCCTAGGGGGATGACGGTACCTGAAGAATAAGCACCGGCTAACTACGTGCCAGCAGCCGCGGTAATACGTAGGGTGCAAGCGTTAATCGGAATTACTGGGCGTAAAGCGTGCGCAGGCGGTTGTGTAAGACAGGTGTGAAATCCCCGGGCTTAACCTGGGAATTGCATTTGTGACTGCACGACTAGAGTGTGTCAGAGGGGGGTGGAATTCCACGTGTAGCAGTGAAATGCGTAGATATGTGGAGGAACACCAATGGCGAAGGCAGCCCCCTGGGATAACACTGACGCTCATGCACGAAAGCGTGGGGAGCAAACAGGATTAGATACCCCGGTAGTC |
| OTU_000736 | NR_025421.1 | 98.28 | *Limnobacter thiooxidans* | Burkholderiaceae | CCTACGGGCGGCTGCAGTGAGGAATATTGGACAATGGGCGAGAGCCTGATCCAGCAATGCCGCGTGTGCGAAGAAGGCCTTCGGGTTGTAAAGCACTTTTGTCAGGGAAGAAATCCTTTGGGCTAATACCCTAGGGGGATGACGGTACCTGAAGAATAAGCACCGGCTAACTACGTGCCAGCAGCCGCGGTAATACGTAGGGTGCAAGCGTTAATCGGAATTACTGGGCGTAAAGCGTGCGCAGGCGGTTGTGTAAGACAGGTGTGAAATCCCCGGGCTTAACCTGGGAATTGCATTTGTGACTGCACGACTAGAGTGTGTCAGAGGGGGGTGGAATTCCACGTGTAGCAGTGAAATGCGTAGATATGTGGAGGAACACCAATGGCGAAGGCAGCCCCCTGGGATAACACTGACGCTCATGCACGAAAGCGTGGGGAGCAAACAGGATTAGATACCCTGGTAGTC |
| OTU_000815 | NR_025421.1 | 95.94 | *Limnobacter thiooxidans* | Burkholderiaceae | CCTACGGGGGGCTGCAGTGGGGAATTTTGGACAATGGGGGAAACCCTGATCCAGCAATGCCGCGTGTGCGAAGAAGGCCTTCGGGTTGTAAAGCACTTTTGTCAGGGAAGAAATCCTTTGGGCTAATACCCTAGGGGGATGACGGTACCTGAAGAATAAGCACCGGCTAACTACGTGCCAGCAGCCGCGGTAATACGTAGGGTGCGAGCGTTAATCGGAATTACTGGGCGTAAAGCGTGCGCAGGCGGATTGTTAAGCAAGATGTGAAATCCCCGGGCTTAACCTGGGAATGGCATTTTGAACTGGCAGTCTAGAGTGTGTCAGAGGGGGGTGGAATTCCACGTGTAGCAGTGAAATGCGTAGAGATGTGGAGGAATACCAATGGCGAAGGCAGCCCCCTGGGATAATATTGACGCTCATGCACGAAAGCGTGGGGAGCAAACAGGATTAGATACCCCAGTAGTC |
| OTU_000832 | NR_025421.1 | 98.06 | *Limnobacter thiooxidans* | Burkholderiaceae | CCTCCGGGCGGCTGCCGTGGGGCCTTTTGGCCAATGGGGGAAACCCTGATCCAGCAATGCCGCGTGTGCGAAGAAGGCCTTCGGGTTGTAAAGCACTTTTGTCAGGGAAGAAATCCTTTGGGCTAATACCCTAGGGGGATGACGGTACCTGAAGAATAAGCACCGGCTAACTACGTGCCAGCAGCCGCGGTAATACGTAGGGTGCAAGCGTTAATCGGAATTACTGGGCGTAAAGCGTGCGCAGGCGGTTGTGTAAGACAGGTGTGAAATCCCCGGGCTTAACCTGGGAATTGCATTTGTGACTGCACGACTAGAGTGTGTCAGAGGGGGGTGGAATTCCACGTGTAGCAGTGAAATGCGTAGATATGTGGAGGAACACCAATGGCGAAGGCAGCCCCCTGGGATAACACTGACGCTCATGCACGAAAGCGTGGGGAGCAAACAGGATTAGATACCCGGGTAGTC |
| OTU_001066 | NR_025421.1 | 99.35 | *Limnobacter thiooxidans* | Burkholderiaceae | CCTAGGGCGGCAGCAGTGGGGAATTTTGGACAATGGGGGAAACCCTGATCCAGCAATGCCGCGTGTGCGAAGAAGGCCTTCGGGTTGTAAAGCACTTTTGTCAGGGAAGAAATCCTTTGGGCTAATACCCTAGGGGGATGACGGTACCTGAAGAATAAGCACCGGCTAACTACGTGCCAGCAGCCGCGGTAATACGTAGGGTGCAAGCGTTAATCGGAATTACTGGGCGTAAAGCGTGCGCAGGCGGTTGTGTAAGACAGGTGTGAAATCCCCGGGCTTAACCTGGGAATTGCATTTGTGACTGCACGACTAGAGTGTGTCAGAGGGGGGTGGAATTCCACGTGTAGCAGTGAAATGCGTAGATATGTGGAGGAACACCAATGGCGAAGGCAGCCCCCTGGGATAACACTGACGCTCATGCACGAAAGCGTGGGGAGCAAACAGGATTAGATACCCTGGTAGTC |
| OTU_001135 | NR_025421.1 | 98.28 | *Limnobacter thiooxidans* | Burkholderiaceae | CCTACGGGGGGCTGCAGTGGGGAATTTTGGACAATGGGGGAAACCCTGATCCAGCAATGCCGCGTGTGCGAAGAAGGCCTTCGGGTTGTAAAGCACTTTTGTCAGGGAAGAAATCCTTTGGGCTAATACCCTAGGGGGATGACGGTACCTGAAGAATAAGCACCGGCTAACTACGTGCCAGCAGCCGCGGTAATACGTAGGGTGCAAGCGTTAATCGGAATTACTGGGCGTAAAGCGTGCGCAGGCGGTTGTGTAAGACAGGTGTGAAATCCCCGGGCTTAACCTGGGAATTGCATTTGTGACTGCACGACTAGAGTGTGTCAGAGGGGGGTGGAATTCCACGTGTAGCAGTGAAATGCGTAGATATGTGGAGGAACACCAATGGCGAAGGCAGCCCCCTGGGATAACACTGACGCTGAGGTGCGAAAGTGTGGGGAGCAAACAGGATTAGATACCCTGGTAGTC |
| OTU_001765 | NR_025421.1 | 98.06 | *Limnobacter thiooxidans* | Burkholderiaceae | CCTACGGGTGGCAGCAGTGGGGAATTTTGGACAATGGGGGAAACCCTGATCCAGCAATGCCGCGTGTGCGAAGAAGGCCTTCGGGTTGTAAAGCACTTTTGTCAGGGAAGAAATCCTTTGGGCTAATACCCTAGGGGGATGACGGTACCTGAAGAATAAGCACCGGCTAACTACGTGCCAGCAGCCGCGGTAATACGTAGGGTGCAAGCGTTAATCGGAATTACTGGGCGTAAAGCGTGCGCAGGCGGTTGTGTAAGACAGGTGTGAAATCCCCGGGCTTAACCTGGGAACTGCGCTTGTGACTGCACGGCTAGAGTATGGCAGAGGGGGGTGGAATTCCACGTGTAGCAGTGAAATGCGTAGATATGTGGAGGAACACCAATGGCGAAGGCAGCCCCCTGGGATAACACTGACGCTCATGCACGAAAGCGTGGGGAGCAAACAGGATTAGATACCCCGGTAGTC |
| OTU_002028 | NR_025421.1 | 99.14 | *Limnobacter thiooxidans* | Burkholderiaceae | CCTACGGGTGGCAGCAGTGGGGAATTTTGGACAATGGGGGAAACCCTGATCCAGCAATGCCGCGTGTGCGAAGAAGGCCTTCGGGTTGTAAAGCACTTTTGTCAGGGAAGAAATCCTTTGGGCTAATACCCTAGGGGGATGACGGTACCTGAAGAATAAGCACCGGCTAACTACGTGCCAGCAGCCGCGGTAATACGTAGGGTGCAAGCGTTAATCGGAATTACTGGGCGTAAAGCGTGCGCAGGCGGTTGTGTAAGACAGGTGTGAAATCCCCGGGCTTAACCTGGGAATTGCATTTGTGACTGCACGACTAGAGTGTGTCAGAGGGGGGTGGAATTCCACGTGTAGCAGTGAAATGCGTAGATATGTGGAGGAACACCAATGGCGAAGGCAGCCCCCTGGGATAACACTGACGCTCATGCACGAAAGCGTGGGGAGCAAACAGGATTAGATACCCTTGTGTC |
| OTU_003210 | NR_025421.1 | 97.85 | *Limnobacter thiooxidans* | Burkholderiaceae | CCTACGGGGGGCAGCAGTGGGGAATTTTGGACAATGGGGGAAACCCTGATCCAGCAATGCCGCGTGTGTGAAGAAGGCCTTCGGGTTGTAAAGCACTTTCGTCAGGGAAGAAATCCTTTGGGCTAATACCCTAGGGGGATGACGGTACCTGAAGAATAAGCACCGGCTAACTACGTGCCAGCAGCCGCGGTAATACGTAGGGTGCAAGCGTTAATCGGAATTACTGGGCGTAAAGCGTGCGCAGGCGGTTGTGTAAGACAGGTGTGAAATCCCCGGGCTTAACCTGGGAATTGCATTTGTGACTGCACGACTAGAGTGTGTCAGAGGGGGGTGGAATTCCACGTGTAGCGGTGAAATGCGTAGATATGTGGAGGAACACCAGTGGCGAAGGCGGCCCCCTGGGATAACACTGACGCTCATGCGCGAAAGCGTGGGGAGCAAACAGGATTAGATACCCCTGTAGTC |
| OTU_000017 | NR_029023.1 | 98.92 | *Hydrogenophaga atypica* | Comamonadaceae | CCTACGGGGGGCTGCAGTGGGGAATTTTGGACAATGGGCGCAAGCCTGATCCAGCAATGCCGCGTGCAGGAAGAAGGCCTTCGGGTTGTAAACTGCTTTTGTACGGAACGAAACGGCCCTGGTTAATACCTGGGGCTAATGACGGTACCGTAAGAATAAGCACCGGCTAACTACGTGCCAGCAGCCGCGGTAATACGTAGGGTGCAAGCGTTAATCGGAATTACTGGGCGTAAAGCGTGCGCAGGCGGTTTTGTAAGACAGGCGTGAAATCCCCGGGCTTAACCTGGGAATGGCGCTTGTGACTGCAAAGCTGGAGTGCGGCAGAGGGGGATGGAATTCCGCGTGTAGCAGTGAAATGCGTAGATATGCGGAGGAACACCGATGGCGAAGGCAATCCCCTGGGCCTGCACTGACGCTCATGCACGAAAGCGTGGGGAGCAAACAGGATTAGATACCCCGGTAGTC |
| OTU_000082 | NR_114130.1 | 99.14 | *Hydrogenophaga pseudoflava* | Comamonadaceae | CCTACGGGGGGCTGCAGTGGGGAATTTTGGACAATGGGCGCAAGCCTGATCCAGCAATGCCGCGTGCAGGAAGAAGGCCTTCGGGTTGTAAACTGCTTTTGTACGGAACGAAACGGTCTGGGTTAATACCCTGGGCTAATGACGGTACCGTAAGAATAAGCACCGGCTAACTACGTGCCAGCAGCCGCGGTAATACGTAGGGTGCAAGCGTTAATCGGAATTACTGGGCGTAAAGCGTGCGCAGGCGGTTTTGTAAGACAGGCGTGAAATCCCCGGGCTCAACCTGGGAATTGCGCTTGTGACTGCAAGGCTGGAGTGCGGCAGAGGGGGATGGAATTCCGCGTGTAGCAGTGAAATGCGTAGATATGCGGAGGAACACCGATGGCGAAGGCAATCCCCTGGGCCTGCACTGACGCTCATGCACGAAAGCGTGGGGAGCAAACAGGATTAGATACCCCGGTAGTC |
| OTU_000199 | NR_125536.1 | 98.71 | *Rhodoferax saidenbachensis* | Comamonadaceae | CCTACGGGGGGCTGCAGTGGGGAATTTTGGACAATGGGCGCAAGCCTGATCCAGCAATGCCGCGTGCAGGACGAAGGCCTTCGGGTTGTAAACTGCTTTTGTACGGAACGAAACGGCTCCTTCTAATACAGGGGGCTAATGACGGTACCGTAAGAATAAGCACCGGCTAACTACGTGCCAGCAGCCGCGGTAATACGTAGGGTGCGAGCGTTAATCGGAATTACTGGGCGTAAAGCGTGCGCAGGCGGTTATATAAGACAGATGTGAAATCCCCGGGCTCAACCTGGGACCTGCATTTGTGACTGTATAGCTAGAGTACGGTAGAGGGGGATGGAATTCCGCGTGTAGCAGTGAAATGCGTAGATATGCGGAGGAACACCGATGGCGAAGGCAATCCCCTGGACCTGTACTGACGCTCATGCACGAAAGCGTGGGGAGCAAACAGGATTAGATACCCTGGTAGTC |
| OTU_000203 | NR_125491.1 | 98.92 | *Limnohabitans curvus* | Comamonadaceae | CCTACGGGGGGCTGCAGTGGGGAATTTTGGACAATGGACGCAAGTCTGATCCAGCCATTCCGCGTGCAGGACGAAGGCCTTCGGGTTGTAAACTGCTTTTGTACAGAACGAAAAGGTCTCTATTAATACTAGGGGCTCATGACGGTACTGTAAGAATAAGCACCGGCTAACTACGTGCCAGCAGCCGCGGTAATACGTAGGGTGCAAGCGTTAATCGGAATTACTGGGCGTAAAGCGTGCGCAGGCGGTTATATAAGACAGATGTGAAATCCCCGGGCTCAACCTGGGAACTGCATTTGTGACTGTATAGCTGGAGTGCGGCAGAGGGGGATGGAATTCCGCGTGTAGCAGTGAAATGCGTAGATATGCGGAGGAACACCGATGGCGAAGGCAATCCCCTGGGCCTGCACTGACGCTCATGCACGAAAGCGTGGGGAGCAAACAGGATTAGATACCCCAGTAGTC |
| OTU_000224 | NR_113622.1 | 98.71 | *Simplicispira psychrophila* | Comamonadaceae | CCTACGGGGGGCTGCAGTGGGGAATTTTGGACAATGGGCGAAAGCCTGATCCAGCCATGCCGCGTGCAGGATGAAGGCCTTCGGGTTGTAAACTGCTTTTGTACGGAACGAAAAGGTCTTTTCTAATAAAGAAGGCTCATGACGGTACCGTAAGAATAAGCACCGGCTAACTACGTGCCAGCAGCCGCGGTAATACGTAGGGTGCAAGCGTTAATCGGAATTACTGGGCGTAAAGCGTGCGCAGGCGGTTATATAAGACAGATGTGAAATCCCCGGGCTCAACCTGGGAACTGCATTAGTGACTGTATAGCTAGAGTGCGGCAGAGGGGGATGGAATTCCGCGTGTAGCAGTGAAATGCGTAGATATGCGGAGGAACACCGATGGCGAAGGCAATCCCCTGGGCCTGCACTGACGCTCATGCACGAAAGCGTGGGGAGCAAACAGGATTAGATACCCTAGTAGTC |
| OTU_000257 | NR_043769.1 | 98.71 | *Hydrogenophaga caeni* | Comamonadaceae | CCTACGGGGGGCTGCAGTGGGGAATTTTGGACAATGGGCGAAAGCCTGATCCAGCAATGCCGCGTGCAGGAAGAAGGCCTTCGGGTTGTAAACTGCTTTTGTACGGAGCGAAAAGGCTCTCTCTAATACAGGGGGCTCATGACGGTACCGTAAGAATAAGCACCGGCTAACTACGTGCCAGCAGCCGCGGTAATACGTAGGGTGCAAGCGTTAATCGGAATTACTGGGCGTAAAGCGTGCGCAGGCGGTTTTGTAAGACAGGCGTGAAATCCCCGGGCTCAACCTGGGAATGGCGCTTGTGACTGTAAAGCTGGAGTGCGGCAGAGGGGGATGGAATTCCGCGTGTAGCAGTGAAATGCGTAGATATGCGGAGGAACACCGATGGCGAAGGCAATCCCCTGGGCCTGCACTGACGCTCATGCACGAAAGCGTGGGGAGCAAACAGGATTAGATACCCGAGTAGTC |
| OTU_000260 | NR_114228.1 | 96.56 | *Malikia spinosa* | Comamonadaceae | CCTACGGGTGGCTGCAGTGGGGAATTTTGGACAATGGGCGAAAGCCTGATCCAGCAATACCGCGTGCAGGAAGAAGGCCTTCGGGTTGTAAACTGCTTTTGTACGGAACGAAAAGGTCTGGGTTAATACCCTGGGCTCATGACGGTACCGTAAGAATAAGCACCGGCTAACTACGTGCCAGCAGCCGCGGTAATACGTAGGGTGCAAGCGTTAATCGGAATTACTGGGCGTAAAGCGTGCGCAGGCGGTTATGTAAGACAGGCGTGAAATCCCCGGGCTCAACCTGGGAATTGCGCTTGTGACTGCATAACTAGAGTACGGTAGAGGGGGATGGAATTCCGCGTGTAGCAGTGAAATGCGTAGATATGCGGAGGAACACCAATGGCGAAGGCAGTCCCCTGGACCTGTACTGACGCTCATGCACGAAAGCGTGGGGAGCAAACAGGATTAGATACCCCAGTAGTC |
| OTU_000261 | NR_114131.1 | 99.57 | *Hydrogenophaga taeniospiralis* | Comamonadaceae | CCTACGGGTGGCAGCAGTGGGGAATTTTGGACAATGGGCGCAAGCCTGATCCAGCAATGCCGCGTGCAGGAAGAAGGCCTTCGGGTTGTAAACTGCTTTTGTACGGAACGAAACGGTCTGGGTTAATACCCTGGGCTAATGACGGTACCGTAAGAATAAGCACCGGCTAACTACGTGCCAGCAGCCGCGGTAATACGTAGGGTGCAAGCGTTAATCGGAATTACTGGGCGTAAAGCGTGCGCAGGCGGTTTTGTAAGACAGTCGTGAAATCCCCGGGCTCAACCTGGGAATTGCGATTGTGACTGCAAAGCTGGAGTGCGGCAGAGGGGGATGGAATTCCGCGTGTAGCAGTGAAATGCGTAGATATGCGGAGGAACACCGATGGCGAAGGCAATCCCCTGGGCCTGCACTGACGCTCATGCACGAAAGCGTGGGGAGCAAACAGGATTAGATACCCTAGTAGTC |
| OTU_000291 | NR_113736.1 | 98.49 | *Variovorax paradoxus* | Comamonadaceae | CCTACGGGGGGCTGCAGTGGGGAATTTTGGACAATGGGCGCAAGCCTGATCCAGCCATGCCGCGTGCAGGATGAAGGCCTTCGGGTTGTAAACTGCTTTTGTACGGAACGAAACGGCCTTTTCTAATAAAGAGGGCTAATGACGGTACCGTAAGAATAAGCACCGGCTAACTACGTGCCAGCAGCCGCGGTAATACGTAGGGTGCAAGCGTTAATCGGAATTACTGGGCGTAAAGCGTGCGCAGGCGGTGATGTAAGACAGTTGTGAAATCCCCGGGCTCAACCTGGGAACTGCATCTGTGACTGCATCGCTGGAGTACGGCAGAGGGGGATGGAATTCCGCGTGTAGCAGTGAAATGCGTAGATATGCGGAGGAACACCGATGGCGAAGGCAATCCCCTGGGCCTGTACTGACGCTCATGCACGAAAGCGTGGGGAGCAAACAGGATTAGATACCCGAGTAGTC |
| OTU_000338 | NR_043769.1 | 97.64 | *Hydrogenophaga caeni* | Comamonadaceae | CCTACGGGTGGCTGCAGTGGGGAATTTTGGACAATGGGCGCAAGCCTGATCCAGCAATGCCGCGTGCAGGAAGAAGGCCTTCGGGTTGTAAACTGCTTTTGTACGGAGCGAAAAGGTCTTCCCTAATACGGGAGGCTGATGACGGTACCGTAAGAATAAGCACCGGCTAACTACGTGCCAGCAGCCGCGGTAATACGTAGGGTGCGAGCGTTAATCGGAATTACTGGGCGTAAAGCGTGCGCAGGCGGTTTTGTAAGACAGGCGTGAAATCCCCGGGCTCAACCTGGGAATGGCGCTTGTGACTGCAAAGCTGGAGTGCGGCAGAGGGGGATGGAATTCCGCGTGTAGCAGTGAAATGCGTAGATATGCGGAGGAACACCGATGGCGAAGGCAATCCCCTGGGCCTGCACTGACGCTCATGCACGAAAGCGTGGGGAGCAAACAGGATTAGATACCCCAGTAGTC |
| OTU_000481 | NR_029024.1 | 98.71 | *Hydrogenophaga defluvii* | Comamonadaceae | CCTACGGGTGGCAGCAGTGGGGAATTTTGGACAATGGGCGCAAGCCTGATCCAGCAATGCCGCGTGCAGGAAGAAGGCCTTCGGGTTGTAAACTGCTTTTGTACGGAACGAAACGGCCCTGGTTAATACCTGGGGCTAATGACGGTACCGTAAGAATAAGCACCGGCTAACTACGTGCCAGCAGCCGCGGTAATACGTAGGGTGCAAGCGTTAATCGGAATTACTGGGCGTAAAGCGTGCGCAGGCGGTTTTGTAAGACAGGCGTGAAATCCCCGGGCTCAACCTGGGAATTGCGCTTGTGACTGCAAGGCTGGAGTGCGGCAGAGGGGGATGGAATTCCGCGTGTAGCAGTGAAATGCGTAGATATGCGGAGGAACACCGATGGCGAAGGCAATCCCCTGGGCCTGCACTGACGCTCATGCACGAAAGCGTGGGGAGCAAACAGGATTAGATACCCGTGTAGTC |
| OTU_000819 | NR_043769.1 | 99.57 | *Hydrogenophaga caeni* | Comamonadaceae | CCTACGGGAGGCAGCAGTGGGGAATTTTGGACAATGGGCGCAAGCCTGATCCAGCAATGCCGCGTGCAGGAAGAAGGCCTTCGGGTTGTAAACTGCTTTTGTACGGAGCGAAAAAGCTCTCTCTAATACAGGGGGCTCATGACGGTACCGTAAGAATAAGCACCGGCTAACTACGTGCCAGCAGCCGCGGTAATACGTAGGGTGCAAGCGTTAATCGGAATTACTGGGCGTAAAGCGTGCGCAGGCGGTTTTGTAAGACAGGCGTGAAATCCCCGGGCTCAACCTGGGAATGGCGCTTGTGACTGCAAAGCTGGAGTGCGGCAGAGGGGGATGGAATTCCGCGTGTAGCAGTGAAATGCGTAGATATGCGGAGGAACACCGATGGCGAAGGCAATCCCCTGGGCCTGCACTGACGCTCATGCACGAAAGCGTGGGGAGCAAACAGGATTAGATACCCTTGTAGTC |
| OTU_001638 | NR_029023.1 | 98.71 | *Hydrogenophaga atypica* | Comamonadaceae | CCTACGGGAGGCAGCAGTGGGGAATTTTGGACAATGGGCGCAAGCCTGATCCAGCAATGCCGCGTGCAGGAAGAAGGCCTTCGGGTTGTAAACTGCTTTTGTACGGAACGAAACGGTCCTGGTTAATACCTGGGGCTAATGACGGTACCGTAAGAATAAGCACCGGCTAACTACGTGCCAGCAGCCGCGGTAATACGTAGGGTGCAAGCGTTAATCGGAATTACTGGGCGTAAAGCGTGCGCAGGCGGTTTTGTAAGACAGGCGTGAAATCCCCGGGCTTAACCTGGGAATGGCGCTTGAGACTGCAAAGCTGGAGTGCGGCAGAGGGGGATGGAATTCCGCGTGTAGCAGTGAAATGCGTAGATATGCGGAGGAACACCGGTGGCGAAGGCAATCCCCTGGGCCTGCACTGACGCTCATGCACGAAAGCGTGGGGAGCAAACAGGATTAGATACCCTAGTAGTC |
| OTU_002077 | NR_149183.1 | 97.63 | *Hydrogenophaga laconesensis* | Comamonadaceae | CCTACGGGCGGCTGCAGTGGGGAATTTTGGACAATGGGCGCAAGCCTGATCCAGCAATGCCGCGTGCAGGAAGAAGGCCTTCGGGTTGTAAACTGCTTTTGTACGGAACGAAACGGTCCTGGTTAATACCTGGGGCTAATGACGGTACCGTAAGAATAAGCACCGGCTAACTACGTGCCAGCAGCCGCGGTAATACGTAGGGTGCAAGCGTTAATCGGAATTACTGGGCGTAAAGCGTGCGCAGGCGGTTTTGTAAGACAGGCGTGAAATCCCCGGGCTTAACCTGGGAATGGCGCTTGTGACTGCAAAGCTGGAGTGCGGCAGAGGGGGATGGAATTCCGCGTGTAGCAGTGAAATGCGTAGATATGCGGAGGAACACCGATGGCGAAGGCAATCCCCTGGGATAACACTGACGCTCATGCACGAAAGCGTGGGGAGCAAACAGGATTAGATACCCCTGTAGTC |
| OTU_002125 | NR_029024.1 | 98.49 | *Hydrogenophaga defluvii* | Comamonadaceae | CCTACGGGAGGCAGCAGTGGGGAATTTTGGACAATGGGGGAAACCCTGATCCAGCAATGCCGCGTGCAGGAAGAAGGCCTTCGGGTTGTAAACTGCTTTTGTACGGAACGAAACGGCCCTGGTTAATACCTGGGGCTAATGACGGTACCGTAAGAATAAGCACCGGCTAACTACGTGCCAGCAGCCGCGGTAATACGTAGGGTGCAAGCGTTAATCGGAATTACTGGGCGTAAAGCGTGCGCAGGCGGTTTTGTAAGACAGGCGTGAAATCCCCGGGCTTAACCTGGGAATGGCGCTTGTGACTGCAAAGCTGGAGTGCGGCAGAGGGGGATGGAATTCCGCGTGTAGCAGTGAAATGCGTAGATATGCGGAGGAACACCGATGGCGAAGGCAATCCCCTGGGCCTGCACTGACGCTCATGCACGAAAGCGTGGGGAGCAAACAGGATTAGATACCCGAGTAGTC |
| OTU_000034 | NR_117782.1 | 92.72 | *Ferrovum myxofaciens* | Ferrovaceae | CCTACGGGGGGCTGCAGTGGGGAATTTTGGACAATGGGGGAAACCCTGATCCAGCCATTCCGCGTGAGTGAAGAAGGCCTTCGGGTTGTAAAGCTCTTTCGCAAGGGAAGAAAAGATATAGGTGAATAGCTTATGTTGATGACGGTACCTTGACAAGAAGCACCGGCTAACTACGTGCCAGCAGCCGCGGTAATACGTAGGGTGCAAGCGTTAATCGGAATTACTGGGCGTAAAGCGTGCGCAGGCTGTTTTGTAAGTCAGATGTGAAATCCCCGAGCTCAACTTGGGAACTGCGTTTGAAACTACAAGACTAGAATAGGTCAGAGGGGGGTAGAATTCCACGTGTAGCAGTGAAATGCGTAGAGATGTGGAGGAATACCAATGGCGAAGGCAGCCCCCTGGGATCATATTGACGCTCATGCACGAAAGCGTGGGGAGCGAACAGGATTAGATACCCCGGTAGTC |
| OTU_000008 | NR_149769.1 | 97.61 | *Aquaticitalea lipolytica* | Flavobacteriaceae | CCTACGGGGGGCTGCAGTGAGGAATATTGGACAATGGGCGAGAGCCTGATCCAGCCATGCCGCGTGCAGGAAGACGGTCCTATGGATTGTAAACTGCTTTTATACGGGAAGAAACACCCCCTCGTGAGGGGGCTTGACGGTACCGTAAGAATAAGGATCGGCTAACTCCGTGCCAGCAGCCGCGGTAATACGGAGGATCCAAGCGTTATCCGGAATCATTGGGTTTAAAGGGTCCGTAGGTGGATAATTAAGTCAGAGGTGAAATCCTGCAGCTCAACTGTAGAATTGCCTTTGATACTGGTTATCTTGAATTATTATGAAGTAGTTAGAATATGTAGTGTAGCGGTGAAATGCATAGATATTACATAGAATACCAATTGCGAAGGCAGATTACTAATAATTGATTGACACTGATGGACGAAAGCGTGGGGAGCGAACAGGATTAGATACCCCAGTAGTC |
| OTU_000016 | NR_112839.1 | 98.04 | *Flavobacterium saccharophilum* | Flavobacteriaceae | CCTACGGGGGGCTGCAGTGAGGAATATTGGTCAATGGGCGCAAGCCTGAACCAGCCATGCCGCGTGCAGGATGACGGTCCTATGGATTGTAAACTGCTTTTATACGAGAAGAAACACTCCTTCGTGAAGGAGCTTGACGGTATCGTAAGAATAAGGATCGGCTAACTCCGTGCCAGCAGCCGCGGTAATACGGAGGATCCAAGCGTTATCCGGAATCATTGGGTTTAAAGGGTCCGTAGGCGGTTAGATAAGTCAGTGGTGAAAGCCCATCGCTCAACGGTGGAACGGCCATTGATACTGTTTAACTTGAATTATTAGGAAGTAACTAGAATATGTAGTGTAGCGGTGAAATGCTTAGAGATTACATGGAATACCAATTGCGAAGGCAGGTTACTACTAATGGATTGACGCTGATGGACGAAAGCGTGGGTAGCGAACAGGATTAGATACCCCAGTAGTC |
| OTU_000031 | NR_156151.1 | 98.04 | *Polaribacter lacunae* | Flavobacteriaceae | CCTACGGGGGGCTGCAGTGAGGAATATTGGGCAATGGAGGCAACTCTGACCCAGCCATGCCGCGTGCAGGAAGACGGCCCTATGGGTTGTAAACTGCTTTTATACGGGAAGAAACACCCCCTCGTGAGGGGGCTTGACGGTACCGTAAGAATAAGCACCGGCTAACTCCGTGCCAGCAGCCGCGGTAATACGGAGGGTGCAAGCGTTATCCGGAATCATTGGGTTTAAAGGGTCCGCAGGCGGTCAATTAAGTCAGAGGTGAAATCCCATAGCTTAACTATGGAACTGCCTTTGATACTGGTTGACTTGAGTTATACGGAAGTAGATAGAATGTGTAGTGTAGCGGTGAAATGCATAGATATTACACAGAATACCGATTGCGAAGGCAGTCTACTACGTATATACTGACGCTCATGGACGAAAGCGTGGGGAGCGAACGGGATTAGATACCCCGGTAGTC |
| OTU_000292 | NR_112839.1 | 97.17 | *Flavobacterium saccharophilum* | Flavobacteriaceae | CCTACGGGTGGCTGCAGTGAGGAATATTGGTCAATGGGCGCAAGCCTGAACCAGCCATGCCGCGTGCAGGATGACGGTCCTATGGATTGTAAACTGCTTTTATACGAGAAGAAACACTCCTTCGTGAAGGAGCTTGACGGTATCGTAAGAATAAGGATCGGCTAACTCCGTGCCAGCAGCCGCGGTAATACGGAGGATCCAAGCGTTATCCGGAATCATTGGGTTTAAAGGGTCCGTAGGCGGTTAGATAAGTCAGTGGTGAAAGCCCATCGCTCAACGGTGGAACGGCCATTGATACTGTTTAACTTGAATTATTAGGAAGTAACTAGAATATGTAGTGTAGCGGTGAAATGCTTAGAGATTACATGGAATACCAATTGCGAAGGCAGGTTACTACTAATGGATTGACGCTCATGCACGAAAGCGTGGGGAGCAAACAGGATTAGATACCCGTGTAGTC |
| OTU_000313 | NR_112839.1 | 98.04 | *Flavobacterium saccharophilum* | Flavobacteriaceae | CCTACGGGTGGCAGCAGTGGGGAATTTTGGACAATGGGCGCAAGCCTGAACCAGCCATGCCGCGTGCAGGATGACGGTCCTATGGATTGTAAACTGCTTTTATACGAGAAGAAACACTCCTTCGTGAAGGAGCTTGACGGTATCGTAAGAATAAGGATCGGCTAACTCCGTGCCAGCAGCCGCGGTAATACGGAGGATCCAAGCGTTATCCGGAATCATTGGGTTTAAAGGGTCCGTAGGCGGTTAGATAAGTCAGTGGTGAAAGCCCATCGCTCAACGGTGGAACGGCCATTGATACTGTTTAACTTGAATTATTAGGAAGTAACTAGAATATGTAGTGTAGCGGTGAAATGCTTAGAGATTACATGGAATACCAATTGCGAAGGCAGGTTACTACTAATGGATTGACGCTGATGGACGAAAGCGTGGGTAGCGAACAGGATTAGATACCCGTGTAGTC |
| OTU_001189 | NR_112839.1 | 98.26 | *Flavobacterium saccharophilum* | Flavobacteriaceae | CCTACGGGAGGCAGCAGTGAGGAATATTGGTCAATGGGCGAGAGCCTGAACCAGCCATGCCGCGTGCAGGAAGACGGTCCTATGGATTGTAAACTGCTTTTATACGAGAAGAAACACTCCTTCGTGAAGGAGCTTGACGGTATCGTAAGAATAAGGATCGGCTAACTCCGTGCCAGCAGCCGCGGTAATACGGAGGATCCAAGCGTTATCCGGAATCATTGGGTTTAAAGGGTCCGTAGGCGGTTAGATAAGTCAGTGGTGAAAGCCCATCGCTCAACGGTGGAACGGCCATTGATACTGTTTAACTTGAATTATTAGGAAGTAACTAGAATATGTAGTGTAGCGGTGAAATGCTTAGAGATTACATGGAATACCAATTGCGAAGGCAGGTTACTACTAATGGATTGACGCTGATGGACGAAAGCGTGGGTAGCGAACAGGATTAGATACCCTGGTAGTC |
| OTU_001410 | NR_149769.1 | 97.83 | *Aquaticitalea lipolytica* | Flavobacteriaceae | CCTACGGGAGGCAGCAGTGGGGAATCTTGCACAATGGGCGAGAGCCTGATCCAGCCATGCCGCGTGCAGGAAGACGGTCCTATGGATTGTAAACTGCTTTTATACGGGAAGAAACACCCCCTCGTGAGGGGGCTTGACGGTACCGTAAGAATAAGGATCGGCTAACTCCGTGCCAGCAGCCGCGGTAATACGGAGGATCCAAGCGTTATCCGGAATCATTGGGTTTAAAGGGTCCGTAGGTGGATAATTAAGTCAGAGGTGAAATCCTGCAGCTCAACTGTAGAATTGCCTTTGATACTGGTTATCTTGAATTATTATGAAGTAGTTAGAATATGTAGTGTAGCGGTGAAATGCATAGATATTACATAGAATACCAATTGCGAAGGCAGATTACTAATAATTGATTGACACTGATGGACGAAAGCGTGGGGAGCGAACAGGATTAGATACCCTGGTAGTC |
| OTU_002162 | NR_112839.1 | 97.61 | *Flavobacterium saccharophilum* | Flavobacteriaceae | CCTACGGGCGGCAGCAGTGAGGAATATTGGTCAATGGGCGCAAGCCTGAACCAGCCATGCCGCGTGCAGGATGACGGTCCTATGGATTGTAAACTGCTTTTATACGAGAAGAAACACTCCTTCGTGAAGGAGCTTGACGGTATCGTAAGAATAAGGATCGGCTAACTCCGTGCCAGCAGCCGCGGTAATACGGAGGATCCAAGCGTTATCCGGAATCATTGGGTTTAAAGGGTCCGTAGGCGGTTAGATAAGTCAGTGGTGAAAGCCCATCGCTCAACGGTGGAACGGCCATTGATACTGTTTAACTTGAATTATTAGGAAGTAACTAGAATATGTAGTGTAGCGGTGAAATGCTTAGAGATTACATGGAATACCAATTGCGAAGGCAGGTTACTACAAGTGGATTGACGCTGATGGACGAAAGCGTGGGGAGCAAACAGGATTAGATACCCGGGTAGTC |
| OTU_002360 | NR_156151.1 | 97.39 | *Polaribacter lacunae* | Flavobacteriaceae | CCTACGGGCGGCAGCAGTGGGGAATTTTGGACAATGGAGGCAACTCTGACCCAGCCATGCCGCGTGCAGGAAGACGGCCCTATGGGTTGTAAACTGCTTTTATACGGGAAGAAACACCCCCTCGTGAGGGGGCTTGACGGTACCGTAAGAATAAGCACCGGCTAACTCCGTGCCAGCAGCCGCGGTAATACGGAGGGTGCAAGCGTTATCCGGAATCATTGGGTTTAAAGGGTCCGCAGGCGGTCAATTAAGTCAGAGGTGAAATCCCATAGCTTAACTATGGAACTGCCTTTGATACTGGTTGACTTGAGTTATACGGAAGTAGATAGAATGTGTAGTGTAGCGGTGAAATGCATAGATATTACACAGAATACCGATTGCGAAGGCAGTCTACTACGTATATACTGACGCTCATGGACGAAAGCGTGGGGAGCGAACGGGATTAGATACCCGAGTAGTC |
| OTU_002398 | NR_156151.1 | 97.83 | *Polaribacter lacunae* | Flavobacteriaceae | CCTACGGGTGGCAGCAGTGAGGAATATTGGGCAATGGAGGCAACTCTGACCCAGCCATGCCGCGTGCAGGAAGACGGCCCTATGGGTTGTAAACTGCTTTTATACGGGAAGAAACACCCCCTCGTGAGGGGGCTTGACGGTACCGTAAGAATAAGCACCGGCTAACTCCGTGCCAGCAGCCGCGGTAATACGGAGGGTGCAAGCGTTATCCGGAATCATTGGGTTTAAAGGGTCCGCAGGCGGTCAATTAAGTCAGAGGTGAAATCCCATAGCTTAACTATGGAACTGCCTTTGATACTGGTTGACTTGAGTTATACGGAAGTAGATAGAATGTGTAGTGTAGCGGTGAAATGCATAGATATTACACAGAATACCGATTGCGAAGGCAGTCTACTACGTATATACTGACGCTCATGCACGAAAGCGTGGGGAGCAAACAGGATTAGATACCCGTGTAGTC |
| OTU_000059 | NR_074693.1 | 98.06 | *Methylotenera* | Methylophilaceae | CCTACGGGGGGCTGCAGTGGGGAATTTTGGACAATGGGCGAAAGCCTGATCCAGCCATTCCGCGTGAGTGAAGAAGGCCTTCGGGTTGTAAAGCTCTTTCGCAAGAGAAGAAAACTTAGTTACTAATATTAACTGAGGTTGACGGTATCTTGATAAGAAGCACCGGCTAACTACGTGCCAGCAGCCGCGGTAATACGTAGGGTGCGAGCGTTAATCGGAATTACTGGGCGTAAAGCGTGCGCAGGCTGTTTTGTAAGTCAGATGTGAAATCCCCGAGCTCAACTTGGGAACTGCGTTTGAAACTACAAGACTAGAATATGTCAGAGGGGGGTAGAATTCCACGTGTAGCAGTGAAATGCGTAGAGATGTGGAGGAATATCAATGGCGAAGGCAGCCCCCTGGGATAATATTGACGCTCATGCACGAAAGCGTGGGGAGCAAACAGGATTAGATACCCCGGTAGTC |
| OTU_000592 | NR_074693.1 | 94.84 | *Methylotenera* | Methylophilaceae | CCTACGGGGGGCTGCAGTGGGGAATTTTGGACAATGGGGGAAACCCTGATCCAGCCATTCCGCGTGAGTGAAGAAGGCCTTCGGGTTGTAAAGCTCTTTCGCAAGGGAAGAAAAGATACAAATGAATAGTTTGTGTTGATGACGGTACCTTGACAAGAAGCACCGGCTAACTACGTGCCAGCAGCCGCGGTAATACGTAGGGTGCAAGCGTTAATCGGAATTACTGGGCGTAAAGCGTGCGCAGGCTGTTTTGTAAGTCAGATGTGAAATCCCCGAGCTCAACTTGGGAACTGCGTTTGAAACTACAAGACTAGAATAGGTCAGAGGGGGGTAGAATTCCACGTGTAGCAGTGAAATGCGTAGAGATGTGGAGGAATACCAATGGCGAAGGCAGCCCCCTGGGATCATATTGACGCTCATGCACGAAAGCGTGGGGAGCGAACAGGATTAGATACCCGTGTAGTC |
| OTU_000043 | NR_114175.1 | 96.99 | *Oxalicibacterium solurbis* | Oxalobacteraceae | CCTACGGGGGGCTGCAGTGGGGAATTTTGGACAATGGGGGAAACCCTGATCCAGCAATGCCGCGTGTGTGAAGAAGGCCTTCGGGTTGTAAAGCACTTTTGTCAGGAACGAAACGGTGGGGGCTAATATCCTCTGCTAATGACGGTACCTGAAGAATAAGCACCGGCTAACTACGTGCCAGCAGCCGCGGTAATACGTAGGGTGCAAGCGTTAATCGGAATTACTGGGCGTAAAGCGTGCGCAGGCGGTTGTGCAAGACAGATGTGAAATCCCCGGGCTCAACCTGGGAATTGCATTTGTGACTGCACGGCTAGAGTGTGTCAGAGGGGGGTAGAATTCCACGTGTAGCAGTGAAATGCGTAGATATGTGGAGGAATACCGATGGCGAAGGCAGCCCCCTGGGATAACACTGACGCTCATGCACGAAAGCGTGGGGAGCAAACAGGATTAGATACCCCAGTAGTC |
| OTU_003546 | NR_132302.1 | 96.77 | *Paraherbaspirillum soli* | Oxalobacteraceae | CCTACGGGTGGCTGCAGTGGGGAATTTTGGACAATGGGGGAAACCCTGATCCAGCAATGCCGCGTGTGTGAAGAAGGCCTTCGGGTTGTAAAGCACTTTTGTCAGGAACGAAACGGTGGGGGCTAATATCCTCTGCTAATGACGGTACCTGAAGAATAAGCACCGGCTAACTACGTGCCAGCAGCCGCGGTAATACGTAGGGTGCAAGCGTTAATCGGAATTACTGGGCGTAAAGCGTGCGCAGGCGGTTGTGTAAGACAGGTGTGAAATCCCCGGGCTTAACCTGGGAATTGCATTTGTGACTGCACGACTAGAGTGTGTCAGAGGGGGGTGGAATTCCACGTGTAGCAGTGAAATGCGTAGATATGTGGAGGAATACCGATGGCGAAGGCAGCCCCCTGGGATAACACTGACGCTCATGCACGAAAGCGTGGGGAGCAAACAGGATTAGATACCCGAGTAGTC |
| OTU_000005 | NR_136771.1 | 98.87 | *Hoeflea olei* | Phyllobacteriaceae | CCTACGGGGGGCTGCAGTGGGGAATATTGGACAATGGGCGCAAGCCTGATCCAGCCATGCCGCGTGAGTGATGAAGGCCCTAGGGTTGTAAAGCTCTTTCACCGGTGAAGATAATGACGGTAACCGGAGAAGAAGCCCCGGCTAACTTCGTGCCAGCAGCCGCGGTAATACGAAGGGGGCTAGCGTTGTTCGGAATTACTGGGCGTAAAGCGCACGTAGGCGGATCGTTAAGTGAGGGGTGAAATCCCAGGGCTCAACCCTGGAACTGCCTTTCATACTGGCGATCTTGAGTTCGAGAGAGGTGAGTGGAATTCCGAGTGTAGAGGTGAAATTCGTAGATATTCGGAGGAACACCAGTGGCGAAGGCGGCTCACTGGCTCGATACTGACGCTGAGGTGCGAAAGCGTGGGGAGCAAACAGGATTAGATACCCCAGTAGTC |
| OTU_000241 | NR_118230.1 | 99.55 | *Hoeflea phototrophica* | Phyllobacteriaceae | CCTACGGGTGGCTGCAGTGGGGAATATTGGACAATGGGCGCAAGCCTGATCCAGCCATGCCGCGTGTGTGATGAAGGCCCTAGGGTTGTAAAGCACTTTCAACGGTGAAGATAATGACGGTAACCGTAGAAGAAGCCCCGGCTAACTTCGTGCCAGCAGCCGCGGTAATACGAAGGGGGCTAGCGTTGTTCGGAATTACTGGGCGTAAAGCGCACGTAGGCGGATCGTTAAGTGAGGGGTGAAATCCCAGGGCTCAACCCTGGAACTGCCTTTCATACTGGCGATCTTGAGTTCGAGAGAGGTGAGTGGAATTCCGAGTGTAGAGGTGAAATTCGTAGATATTCGGAGGAACACCAGTGGCGAAGGCGGCTCACTGGCTCGATACTGACGCTGAGGTGCGAAAGCGTGGGGAGCAAACAGGATTAGATACCCTGGTAGTC |
| OTU_002632 | NR_108835.1 | 98.41 | *Hoeflea halophila* | Phyllobacteriaceae | CCTACGGGAGGCTGCAGTGGGGAATCTTGCACAATGGGCGAAAGCCTGATGCAGCCATGCCGCGTGAGTGATGAAGGCCCTAGGGTTGTAAAGCTCTTTCACCGGTGAAGATAATGACGGTAACCGGAGAAGAAGCCCCGGCTAACTTCGTGCCAGCAGCCGCGGTAATACGAAGGGGGCTAGCGTTGTTCGGAATTACTGGGCGTAAAGCGCACGTAGGCGGATCGTTAAGTGAGGGGTGAAATCCCAGGGCTCAACCCTGGAACTGCCTTTCATACTGGCGATCTTGAGTTCGAGAGAGGTGAGTGGAATTCCGAGTGTAGAGGTGAAATTCGTAGATATTCGGAGGAACACCAGTGGCGAAGGCGGCTCACTGGCTCGATACTGACGCTGAGGTGCGAAAGCGTGGGGAGCAAACAGGATTAGATACCCGGGTAGTC |
| OTU_002800 | NR_136771.1 | 98.41 | *Hoeflea olei* | Phyllobacteriaceae | CCTACGGGTGGCAGCAGTGGGGAATTTTGGACAATGGGGGAAACCCTGATCCAGCCATGCCGCGTGAGTGATGAAGGCCCTAGGGTTGTAAAGCTCTTTCACCGGTGAAGATAATGACGGTAACCGGAGAAGAAGCCCCGGCTAACTTCGTGCCAGCAGCCGCGGTAATACGAAGGGGGCTAGCGTTGTTCGGAATTACTGGGCGTAAAGCGCACGTAGGCGGATCGTTAAGTGAGGGGTGAAATCCCAGGGCTCAACCCTGGAACTGCCTTTCATACTGGCGATCTTGAGTTCGAGAGAGGTGAGTGGAATTCCGAGTGTAGAGGTGAAATTCGTAGATATTCGGAGGAACACCAGTGGCGAAGGCGGCTCACTGGCTCGATACTGACGCTGAGGTGCGAAAGCGTGGGGAGCAAACAGGATTAGATACCCCGGTAGTC |
| OTU_000091 | NR_025841.1 | 98.49 | *Rubrivivax gelatinosus* | Unclassified Burkholderiales | CCTACGGGGGGCTGCAGTGGGGAATTTTGGACAATGGACGAAAGTCTGATCCAGCCATGCCGCGTGCGGGAAGAAGGCCTTCGGGTTGTAAACCGCTTTTGTCAGGGAAGAAATCTTCTGGGTTAATACCTCGGGAGGATGACGGTACCTGAAGAATAAGCACCGGCTAACTACGTGCCAGCAGCCGCGGTAATACGTAGGGTGCAAGCGTTAATCGGAATTACTGGGCGTAAAGCGTGCGCAGGCGGTTATGTAAGACAGATGTGAAATCCCCGGGCTCAACCTGGGAACTGCATTTGTGACTGCATAGCTTGAGTGCGGCAGAGGGGGATGGAATTCCGCGTGTAGCAGTGAAATGCGTAGATATGCGGAGGAACACCGATGGCGAAGGCAATCCCCTGGGCCTGCACTGACGCTCATGCACGAAAGCGTGGGGAGCAAACAGGATTAGATACCCCGGTAGTC |
| OTU_000165 | NR_025841.1 | 98.71 | *Rubrivivax gelatinosus* | Unclassified Burkholderiales | CCTACGGGGGGCTGCAGTGGGGAATTTTGGACAATGGGCGCAAGCCTGATCCAGCCATGCCGCGTGCGGGAAGAAGGCCTTCGGGTTGTAAACCGCTTTTGTCAGGGAAGAAATCTTCTGGGTTAATACCTCGGGAGGATGACGGTACCTGAAGAATAAGCACCGGCTAACTACGTGCCAGCAGCCGCGGTAATACGTAGGGTGCAAGCGTTAATCGGAATTACTGGGCGTAAAGCGTGCGCAGGCGGTTATGTAAGACAGAGGTGAAATCCCCGGGCTCAACCTGGGAACTGCCTTTGTGACTGCATAGCTTGAGTGCGGCAGAGGGGGATGGAATTCCGCGTGTAGCAGTGAAATGCGTAGATATGCGGAGGAACACCGATGGCGAAGGCAATCCCCTGGGCCTGCACTGACGCTCATGCACGAAAGCGTGGGGAGCAAACAGGATTAGATACCCTAGTAGTC |
| OTU_000192 | NR_025841.1 | 97.42 | *Rubrivivax gelatinosus* | Unclassified Burkholderiales | CCTACGGGGGGCTGCAGTGGGGAATTTTGGACAATGGACGCAAGTCTGATCCAGCCATGCCGCGTGCGGGAAGAAGGCCTTCGGGTTGTAAACCGCTTTTGTCAGGGAAGAAATCTTCTGGGCTAATACCCGAGGAGGATGACGGTACCTGAAGAATAAGCACCGGCTAACTACGTGCCAGCAGCCGCGGTAATACGTAGGGTGCAAGCGTTAATCGGAATTACTGGGCGTAAAGCGTGCGCAGGCGGTTATGCAAGACAGATGTGAAATCCCCGGGCTCAACCTGGGAACTGCATTTGTGACTGCATAGCTGGAGTGCGGCAGAGGGGGATGGAATTCCGCGTGTAGCAGTGAAATGCGTAGATATGCGGAGGAACACCGATGGCGAAGGCAATCCCCTGGGCCTGCACTGACGCTCATGCACGAAAGCGTGGGGAGCAAACAGGATTAGATACCCCAGTAGTC |
| OTU_000246 | NR_026108.1 | 98.49 | *Ideonella dechloratans* | Unclassified Burkholderiales | CCTACGGGGGGCTGCAGTGGGGAATTTTGGACAATGGGCGCAAGCCTGATCCAGCCATGCCGCGTGCGGGAAGAAGGCCTTCGGGTTGTAAACCGCTTTTGTCAGGGAAGAAATCTTCTGGGCTAATACCTCGGGAGGATGACGGTACCTGAAGAATAAGCACCGGCTAACTACGTGCCAGCAGCCGCGGTAATACGTAGGGTGCAAGCGTTAATCGGAATTACTGGGCGTAAAGCGTGCGCAGGCGGTTTTGTAAGACAGAGGTGAAATCCCCGGGCTTAACCTGGGAACTGCCTTTGTGACTGCAAGGCTTGAGTGCGGCAGAGGGGGATGGAATTCCGCGTGTAGCAGTGAAATGCGTAGATATGCGGAGGAACACCGATGGCGAAGGCAATCCCCTGGGCCTGCACTGACGCTCATGCACGAAAGCGTGGGGAGCAAACAGGATTAGATACCCCGGTAGTC |
| OTU_002366 | NR_025841.1 | 97.64 | *Rubrivivax gelatinosus* | Unclassified Burkholderiales | CCTACGGGAGGCTGCAGTGAGGAATATTGGACAATGGGCGCAAGCCTGATCCAGCCATGCCGCGTGCGGGAAGAAGGCCTTCGGGTTGTAAACCGCTTTTGTCAGGGAAGAAATCTTCTGGGCTAATACCCGAGGAGGATGACGGTACCTGAAGAATAAGCACCGGCTAACTACGTGCCAGCAGCCGCGGTAATACGTAGGGTGCAAGCGTTAATCGGAATTACTGGGCGTAAAGCGTGCGCAGGCGGTTATGCAAGACAGATGTGAAATCCCCGGGCTCAACCTGGGAACTGCATTTGTGACTGCATAGCTGGAGTGCGGCAGAGGGGGATGGAATTCCGCGTGTAGCAGTGAAATGCGTAGATATGCGGAGGAACACCGATGGCGAAGGCAATCCCCTGGGCCTGCACTGACGCTCATGCACGAAAGCGTGGGGAGCAAACAGGATTAGATACCCGTGTAGTC |
| OTU_002472 | NR_025841.1 | 98.49 | *Rubrivivax gelatinosus* | Unclassified Burkholderiales | CCTACGGGAGGCAGCAGTGGGGAATTTTGGACAATGGGCGCAAGCCTGATCCAGCCATGCCGCGTGCGGGAAGAAGGCCTTCGGGTTGTAAACCGCTTTTGTCAGGGAGGAAATCTTCTGGGTTAATACCTCGGGAGGATGACGGTACCTGAAGAATAAGCACCGGCTAACTACGTGCCAGCAGCCGCGGTAATACGTAGGGTGCAAGCGTTAATCGGAATTACTGGGCGTAAAGCGTGCGCAGGCGGTTTTGTAAGACAGAGGTGAAATCCCCGGGCTTAACCTGGGAACTGCCTTTGTGACTGCAAAGCTTGAGTGCGGCAGAGGGGGATGGAATTCCGCGTGTAGCAGTGAAATGCGTAGATATGCGGAGGAACACCGATGGCGAAGGCAATCCCCTGGGCCTGCACTGACGCTCATGCACGAAAGCGTGGGGAGCAAACAGGATTAGATACCCGAGTAGTC |

Table S3A - Core microbiome (55 OTUs) of *Alexandrium ostenfeldii*, specifying: OTU, accession number of closest relative in GenBank, % identity, genus and family of that strain and the sequence of the OTU.

| 1. ***A. minutum/*** 2. ***tamarense* Core OTUs** | **ID - GenBank** | **%** | **Genus GenBank** | **Family GenBank** | **Sequence** |
| --- | --- | --- | --- | --- | --- |
| OTU_000019 | NR_074765.1 | 98.92 | Marinobacter adhaerens | Alteromonadaceae | CCTACGGGGGGCTGCAGTGGGGAATATTGGACAATGGGGGCAACCCTGATCCAGCCATGCCGCGTGTGTGAAGAAGGCTTTCGGGTTGTAAAGCACTTTCAGTGAGGAGGAAAACTCTGCGACTAATACTCGTAGGGCTTGACGTTACTCACAGAAGAAGCACCGGCTAACTCCGTGCCAGCAGCCGCGGTAATACGGAGGGTGCAAGCGTTAATCGGAATTACTGGGCGTAAAGCGCGCGTAGGTGGTTTGATAAGCGAGATGTGAAAGCCCCGGGCTTAACCTGGGAACGGCATTTCGAACTGTCAGGCTAGAGTGTGGTAGAGGGTAGTGGAATTTCCTGTGTAGCGGTGAAATGCGTAGATATAGGAAGGAACACCAGTGGCGAAGGCGGCTACCTGGACCAACACTGACACTGAGGTGCGAAAGCGTGGGGAGCAAACAGGATTAGATACCCCGGTAGTC |
| OTU_000445 | NR_074765.1 | 99.57 | Marinobacter adhaerens | Alteromonadaceae | CCTACGGGAGGCAGCAGTGGGGAATATTGGACAATGGGGGCAACCCTGATCCAGCCATGCCGCGTGTGTGAAGAAGGCTTTCGGGTTGTAAAGCACTTTCAGTGAGGAGGAAAACTCTGCGGCTAATACTCGTAGGGCTTGACGTTACTCACAGAAGAAGCACCGGCTAACTCCGTGCCAGCAGCCGCGGTAATACGGAGGGTGCAAGCGTTAATCGGAATTACTGGGCGTAAAGCGCGCGTAGGTGGTTTGATAAGCGAGATGTGAAAGCCCCGGGCTTAACCTGGGAACGGCATTTCGAACTGTCAGGCTAGAGTATGGTAGAGGGTAGTGGAATTTCCTGTGTAGCGGTGAAATGCGTAGATATAGGAAGGAACACCAGTGGCGAAGGCGGCTACCTGGACCAATACTGACACTGAGGTGCGAAAGCGTGGGGAGCAAACAGGATTAGATACCCTTGTAGTC |
| OTU_001290 | NR_074765.1 | 98.28 | Marinobacter adhaerens | Alteromonadaceae | CCTACGGGAGGCAGCAGTCGGGAATATTGGACAATGGGGGCAACCCTGATCCAGCCATGCCGCGTGTGTGAAGAAGGCCCTAGGGTTGTAAAGCACTTTCAGTGAGGAGGAAAACTCTGCGACTAATACTCGTAGGGCTTGACGTTACTCACAGAAGAAGCACCGGCTAACTCCGTGCCAGCAGCCGCGGTAATACGGAGGGTGCAAGCGTTAATCGGAATTACTGGGCGTAAAGCGCGCGTAGGTGGTTTGATAAGCGAGATGTGAAAGCCCCGGGCTTAACCTGGGAACGGCATTTCGAACTGTCAGGCTAGAGTGTGGTAGAGGGTAGTGGAATTTCCTGTGTAGCGGTGAAATGCGTAGATATAGGAAGGAACACCAGTGGCGAAGGCGGCTACCTGGACCAACACTGACACTGAGGTGCGAAAGCGTGGGGAGCAAACAGGATTAGATACCCGAGTAGTC |
| OTU_000002 | NR_025421.1 | 98.92 | Limnobacter thiooxidans | Burkholderiaceae | CCTACGGGGGGCTGCAGTGGGGAATTTTGGACAATGGGGGAAACCCTGATCCAGCAATGCCGCGTGTGCGAAGAAGGCCTTCGGGTTGTAAAGCACTTTTGTCAGGGAAGAAATCCTTTGGGCTAATACCCTAGGGGGATGACGGTACCTGAAGAATAAGCACCGGCTAACTACGTGCCAGCAGCCGCGGTAATACGTAGGGTGCAAGCGTTAATCGGAATTACTGGGCGTAAAGCGTGCGCAGGCGGTTGTGTAAGACAGGTGTGAAATCCCCGGGCTTAACCTGGGAATTGCATTTGTGACTGCACGACTAGAGTGTGTCAGAGGGGGGTGGAATTCCACGTGTAGCAGTGAAATGCGTAGATATGTGGAGGAACACCAATGGCGAAGGCAGCCCCCTGGGATAACACTGACGCTCATGCACGAAAGCGTGGGGAGCAAACAGGATTAGATACCCCAGTAGTC |
| OTU_000573 | NR_025421.1 | 98.92 | Limnobacter thiooxidans | Burkholderiaceae | CCTACGGGTGGCAGCAGTGGGGAATTTTGGACAATGGGGGAAACCCTGATCCAGCAATGCCGCGTGTGCGAAGAAGGCCTTCGGGTTGTAAAGCACTTTTGTCAGGGAAGAAATCCTTTGGGCTAATACCCTAGGGGGATGACGGTACCTGAAGAATAAGCACCGGCTAACTACGTGCCAGCAGCCGCGGTAATACGTAGGGTGCAAGCGTTAATCGGAATTACTGGGCGTAAAGCGTGCGCAGGCGGTTGTGTAAGACAGGTGTGAAATCCCCGGGCTTAACCTGGGAATTGCATTTGTGACTGCACGACTAGAGTGTGTCAGAGGGGGGTAGAATTCCACGTGTAGCAGTGAAATGCGTAGATATGTGGAGGAATACCGATGGCGAAGGCAGCCCCCTGGGATAACACTGACGCTCATGCACGAAAGCGTGGGGAGCAAACAGGATTAGATACCCTGGTAGTC |
| OTU_000641 | NR_025421.1 | 98.71 | Limnobacter thiooxidans | Burkholderiaceae | CCTACGGGAGGCAGCAGTGGGGAATTTTGGACAATGGGCGCAAGCCTGATCCAGCAATGCCGCGTGTGCGAAGAAGGCCTTCGGGTTGTAAAGCACTTTTGTCAGGGAAGAAATCCTTTGGGCTAATACCCTAGGGGGATGACGGTACCTGAAGAATAAGCACCGGCTAACTACGTGCCAGCAGCCGCGGTAATACGTAGGGTGCAAGCGTTAATCGGAATTACTGGGCGTAAAGCGTGCGCAGGCGGTTGTGTAAGACAGGTGTGAAATCCCCGGGCTTAACCTGGGAATTGCATTTGTGACTGCACGACTAGAGTGTGTCAGAGGGGGGTGGAATTCCACGTGTAGCAGTGAAATGCGTAGATATGTGGAGGAACACCAATGGCGAAGGCAGCCCCCTGGGATAACACTGACGCTCATGCACGAAAGCGTGGGGAGCAAACAGGATTAGATACCCGTGTAGTC |
| OTU_002028 | NR_025421.1 | 99.14 | Limnobacter thiooxidans | Burkholderiaceae | CCTACGGGTGGCAGCAGTGGGGAATTTTGGACAATGGGGGAAACCCTGATCCAGCAATGCCGCGTGTGCGAAGAAGGCCTTCGGGTTGTAAAGCACTTTTGTCAGGGAAGAAATCCTTTGGGCTAATACCCTAGGGGGATGACGGTACCTGAAGAATAAGCACCGGCTAACTACGTGCCAGCAGCCGCGGTAATACGTAGGGTGCAAGCGTTAATCGGAATTACTGGGCGTAAAGCGTGCGCAGGCGGTTGTGTAAGACAGGTGTGAAATCCCCGGGCTTAACCTGGGAATTGCATTTGTGACTGCACGACTAGAGTGTGTCAGAGGGGGGTGGAATTCCACGTGTAGCAGTGAAATGCGTAGATATGTGGAGGAACACCAATGGCGAAGGCAGCCCCCTGGGATAACACTGACGCTCATGCACGAAAGCGTGGGGAGCAAACAGGATTAGATACCCTTGTGTC |
| OTU_000004 | NR_112980.1 | 93.71 | Salinirepens amamiensis | Crocinitomicaceae | CCTACGGGGGGCTGCAGTGAGGAATATTGGACAATGGACGAAAGTCTGATCCAGCCATGCCGCGTGCAGGAAGACTGCCCTATGGGTTGTAAACTGCTTTTATTTGGGAATAAACCCCTCTACGTGTAGAGGGCTGAAGGTACCAAACGAATAAGCACCGGCTAACTCCGTGCCAGCAGCCGCGGTAATACGGAGGGTGCAAGCGTTATCCGGAATCATTGGGTTTAAAGGGTCCGCAGGCGGACATATAAGTCAGTGGTGAAAGCCTACAGCTTAACTGTAGAACTGCCATTGATACTGTATGTCTTGAATTCGGTCGAAGTGGGCGGAATGTGTCATGTAGCGGTGAAATGCATAGATATGACACAGAACACCGATAGCGAAGGCAGCTCACTAGGCCTGAATTGACGCTCAGGGACGAAAGCGTGGGGAGCGAACAGGATTAGATACCCCAGTAGTC |
| OTU_000057 | NR_133750.1 | 95.43 | Fluviicola hefeinensis | Crocinitomicaceae | CCTACGGGGGGCTGCAGTGAGGAATATTGGACAATGGGCGCAAGCCTGATCCAGCCATGCCGCGTGCAGGAAGAATGCCCTATGGGTTGTAAACTGCTTTTATTTGGGAATAAACCTCCTTACGTGTAGGGAGCTGAATGTACCAAACGAATAAGCACCGGCTAACTCCGTGCCAGCAGCCGCGGTAATACGGAGGGTGCAAGCGTTATCCGGAATCATTGGGTTTAAAGGGTCCGCAGGCGGACTTATAAGTCAGTGGTGAAAGCCTACAGCTTAACTGTAGAACTGCCATTGATACTGTAAGTCTTGAATTCGGTCGAAGTGGGCGGAATATGTCATGTAGCGGTGAAATGCTTAGATATGACATAGAACACCGATAGCGAAGGCAGCTCACTAGGCCTGGATTGACGCTCAGGGACGAAAGCGTGGGGAGCGAACAGGATTAGATACCCCAGTAGTC |
| OTU_000698 | NR_112980.1 | 93.28 | Salinirepens amamiensis | Crocinitomicaceae | CCTACGGGTGGCAGCAGTGAGGAATATTGGACAATGGACGAAAGTCTGATCCAGCCATGCCGCGTGCAGGAAGACTGCCCTATGGGTTGTAAACTGCTTTTATTTGGGAATAAACCCCTCTACGTGTAGAGGGCTGAAGGTACCAAACGAATAAGCACCGGCTAACTCCGTGCCAGCAGCCGCGGTAATACGGAGGGTGCAAGCGTTATCCGGAATCATTGGGTTTAAAGGGTCCGCAGGCGGACATATAAGTCAGTGGTGAAAGCCTACAGCTTAACTGTAGAACTGCCATTGATACTGTATGTCTTGAATTCGGTCGAGGTGGGCGGAATGTGTCGTGTAGCGGTGAAATGCATAGATATGACACAGAACACCGATGGCGAAGGCAGCTCACTAGGCCTGAATTGACGCTCAGGGACGAAAGCGTGGGGAGCGAACAGGATTAGATACCCGTGTAGTC |
| OTU_001369 | NR_133750.1 | 95.43 | Fluviicola hefeinensis | Crocinitomicaceae | CCTACGGGTGGCAGCAGTGGGGAATCTTAGACAATGGGCGCAAGCCTGATCCAGCCATGCCGCGTGCAGGAAGAATGCCCTATGGGTTGTAAACTGCTTTTATTTGGGAATAAACCTCCTTACGTGTAGGGAGCTGAATGTACCAAACGAATAAGCACCGGCTAACTCCGTGCCAGCAGCCGCGGTAATACGGAGGGTGCAAGCGTTATCCGGAATCATTGGGTTTAAAGGGTCCGCAGGCGGACTTATAAGTCAGTGGTGAAAGCCTACAGCTTAACTGTAGAACTGCCATTGATACTGTAAGTCTTGAATTCGGTCGAAGTGGGCGGAATATGTCATGTAGCGGTGAAATGCTTAGATATGACATAGAACACCGATAGCGAAGGCAGCTCACTAGGCCTGGATTGACGCTCAGGGACGAAAGCGTGGGGAGCGAACAGGATTAGATACCCTGGTAGTC |
| OTU_001587 | NR_112980.1 | 92.84 | Salinirepens amamiensis | Crocinitomicaceae | CCTACGGGTGGCTGCAGTGAGGAATATTGGACAATGGACGAAAGTCTGATCCAGCCATGCCGCGTGCAGGAAGACTGCCCTATGGGTTGTAAACTGCTTTTATTTGGGAATAAACCCCTCTACGTGTAGAGGGCTGAAGGTACCAAACGAATAAGCACCGGCTAACTCCGTGCCAGCAGCCGCGGTAATACGGAGGGTGCAAGCGTTATCCGGAATCATTGGGTTTAAAGGGTCCGCAGGCGGACATATAAGTCAGTGGTGAAAGCCTACAGCTTAACTGTAGAACTGCCATTGATACTGTATGTCTTGAATTCGGTCGAAGTGGGCGGAATGTGTCATGTAGCGGTGAAATGCATAGATATGACACAGAACACCGATAGCGAAGGCAGCTCACTAGGCCTGAATTGACGCTCATGTGCGAAAGTGTGGGTAGCGAACAGGATTAGATACCCTAGTAGTC |
| OTU_003354 | NR_112980.1 | 93.93 | Salinirepens amamiensis | Crocinitomicaceae | CCTACGGGAGGCAGCAGTGAGGAATATTGGACAATGGACGAAAGTCTGATCCAGCCATGCCGCGTGCAGGAAGACTGCCCTATGGTTTGTAAACTGCTTTTATTTGGGAATAAACCCCTCTACGTGTAGAGGGCTGAAGGTACCACACGAATAAGCACCGGCTAACTCCGTGCCAGCAGCCGCGGTAATACGGAGGGTGCAAGCGTTATCCGGAATCATTGGGTTTAAAGGGTCCGCAGGCGGACATATAAGTCAGTGGTGAAAGCCTACAGCTTAACTGTAGAACTGCCATTGATACTGTATGTCTTGAATTCGGTCGAAGTGGGCGGAATGTGTCATGTAGCGGTGAAATGCATAGATATGAGACAGAACACCGATAGCGAAGGCAGCTCACTAGGCCTGAATTGACGCTCAGGGACGAAAGCGTGGGGAGCGAACAGGATTAGATACCCGGGTAGTC |
| OTU_003481 | NR_112980.1 | 93.71 | Salinirepens amamiensis | Crocinitomicaceae | CCTACGGGAGGCAGCAGTGAGGAATATTGGACAATGGACGAAAGTCTGATCCAGCCATGCCGCGTGCAGGAAGACTGCCCTATGGGTTGTAAACTGCTTTTATTTGGGAATAAACCCCTCTACGTGTAGAGGGCTGAAGGTACCAAACGAATAAGCACCGGCTAACTCCGTGCCAGCAGCCGCGGTAATACGGAGGGTGCAAGCGTTATCCGGAATCATTGGGTTTAAAGGGTCCGCAGGCGGACATATAAGTCAGTGGTGAAAGCCTACAGCTTAACTGTAGAACTGCCATTGATACTGTATGTCTTGAGTTCGGTCGAAGTGGGCGGAATGTGTCATGTAGCGGTGAAATGCATAGATATGACGCAGAACACCGATAGCGAAGGCAGCCCACTAGGCCTGAATTGACGCTCAGGGACGAAAGCGTGGGGAGCGAACAGGATTAGATACCCTTGTAGTC |
| OTU_003559 | NR_112980.1 | 94.01 | Salinirepens amamiensis | Crocinitomicaceae | CCTACGGGTGGCAGCAGTGAGGAATATTGGACAATGGCCGAAAGTCTGATCCAGCCATGCCGCGTGCAGGAAGACTGCCCTATGGGTTGTAAACTGCTTTTATTTGGGAATAAACCCCTCTACGTGTAGAGGGCTGAAGGTACCAAACGAATAAGCACCGGCTAACTCCGTGCCAGCAGCCGCGGTAATACGGAGGGTGCAAGCGTTATCCGGAATCATTGGGTTTAAAGGGTCCGCAGGCGGACATATAAGTCAGTGGTGAAAGCCTACAGCTTAACTGTAGAACTGCCATTGATACTGTATGTCTTGAATTCGGTCGAAGTGGGCGGAATGTGTCATGTAGCGGTGAAATGCATAGATATGACACAGAACACCGATAGCGAAGGCAGCTCACTAGGCCTGAATTGACGCTCAGGGACGAAAGCGTGGGGAGCGAACAGGATTAGATACGTAGTC |
| OTU_000123 | NR_145843.1 | 99.35 | Taeseokella kangwonensis | Cytophagaceae | CCTACGGGGGGCTGCAGTAGGGAATATTGGTCAATGGACGAGAGTCTGAACCAGCCATGCCGCGTGCAGGAAGACGGCCCTCTGGGTTGTAAACTGCTTTTATATGGGAAGAAAAGACTCCTGCGGGAGGCATTGACGGTACCATAGGAATAAGCCACGGCTAACTACGTGCCAGCAGCCGCGGTAATACGTAGGTGGCGAGCGTTATCCGGATTTATTGGGTTTAAAGGGTGCGTAGGCGGCCTGTTAAGTCGGTGGTTAAAGGTAGCAGCTTAACTGTTTTACATGCCATCGATACTGACAGGCTTGAGTTATCAGAAGGCAGGCGGAATTTCTGGTGTAGCGGTGAAATGCATAGATACCAGAAGGAACACCTATTGCGAAGGCAGCTTGCTGCAGATAAACTGACGCTGATGCACGAAAGCGTGGGGAGCGAACAGGATTAGATACCCCGGTAGTC |
| OTU_000131 | NR_137379.1 | 97.17 | Fabibacter misakiensis | Flammeovirgaceae | CCTACGGGGGGCTGCAGTAGGGAATATTGGTCAATGGGCGAGAGCCTGAACCAGCCATGCCGCGTGTAGGAAGACGGCTTTCTGAGTTGTAAACTACTTTTATATGGGAAGAAAAAGGCCATGCGTGGCAAATTGCCGGTACCATATGAATAAGCACCGGCTAACTCCGTGCCAGCAGCCGCGGTAATACGGAGGGTGCAAGCGTTGTCCGGATTTATTGGGTTTAAAGGGTGCGTAGGCGGGTCTTTAAGTCAGTGGTGAAAGCCTGCAGCTTAACTGTAGAACTGCCATTGATACTGGAGACCTTGAGTATACTAGAGGTAGGCGGAATTTATGGTGTAGCGGTGAAATGCATAGATACCATAAAGAACACCGATAGCGTAGGCAGCTTACTGGAGTATAACTGACGCTGATGCACGAAAGCATGGGTAGCGAACAGGATTAGATACCCCGGTAGTC |
| OTU_000033 | NR_025326.1 | 96.36 | Hyphomonas jannaschiana | Hyphomonadaceae | CCTACGGGGGGCTGCAGTGGGGAATATTGCACAATGGGCGCAAGCCTGATGCAGCCATGCCGCGTGAATGATGAAGGCCTTAGGGTTGTAAAATTCTTTCGCCAAGGATGATAATGACAGTACTTGGTAAAGAAGCCCCGGCTAACTTCGTGCCAGCAGCCGCGGTAATACGAAGGGGGCTAGCGTTGTTCGGAATTACTGGGCGTAAAGCGCACGTAGGCGGACTTTTAAGTCAGATGTGAAATCCCAAGGCTCAACCTTGGAACTGCATTTGAAACTGGGAGTCTAGAGATCAGGAGAGGTTAGTGGAATACCGAGTGTAGAGGTGAAATTCGTAGATATTCGGTGGAACACCAGTGGCGAAGGCGACTAACTGGACTGATACTGACGCTGAGGTGCGAAAGTGTGGGGAGCAAACAGGATTAGATACCCCAGTAGTC |
| OTU_000241 | NR_118230.1 | 99.55 | Hoeflea phototrophica | Phyllobacteriaceae | CCTACGGGTGGCTGCAGTGGGGAATATTGGACAATGGGCGCAAGCCTGATCCAGCCATGCCGCGTGTGTGATGAAGGCCCTAGGGTTGTAAAGCACTTTCAACGGTGAAGATAATGACGGTAACCGTAGAAGAAGCCCCGGCTAACTTCGTGCCAGCAGCCGCGGTAATACGAAGGGGGCTAGCGTTGTTCGGAATTACTGGGCGTAAAGCGCACGTAGGCGGATCGTTAAGTGAGGGGTGAAATCCCAGGGCTCAACCCTGGAACTGCCTTTCATACTGGCGATCTTGAGTTCGAGAGAGGTGAGTGGAATTCCGAGTGTAGAGGTGAAATTCGTAGATATTCGGAGGAACACCAGTGGCGAAGGCGGCTCACTGGCTCGATACTGACGCTGAGGTGCGAAAGCGTGGGGAGCAAACAGGATTAGATACCCTGGTAGTC |
| OTU_000310 | NR_042358.1 | 92.97 | Mesorhizobium thiogangeticum | Phyllobacteriaceae | CCTACGGGGGGCTGCAGTGGGGAATATTCGGCAATGGGGGCAACCCTGACCGAGCCATGCCGCGTGTGTGATGAAGGCCCTAGGGTTGTAAAGCACTTTCAACGGTGAAGATAATGACGGTAACCGTAGAAGAAGCTCCGGCTAACTTCGTGCCAGCAGCCGCGGTAATACGAAGGGAGCTAGCGTTGTTCGGAATTACTGGGCGTAAAGCGCGCGTAGGCGGACTGATCAGTCAGGGGTGAAATCCCGGGGCTCAACCCCGGAACTGCCTTTGATACTGTCAGTCTAGAGTCCGGAAGAGGTGAATGGAACTCCTAGTGTAGAGGTGAAATTCGTAGATATTAGTGAAGAACACCAGTGGCGAAGGCGATTCACTGGTCCGGTACTGACGCTGAGGTGCGAAAGCGTGGGGAGCAAACAGGATTAGATACCCCTGTAGTC |
| OTU_000075 | NR_074321.1 | 99.14 | Methylophaga nitratireducenticrescens | Piscirickettsiaceae | CCTACGGGGGGCTGCAGTGGGGAATATTGGACAATGGGCGCAAGCCTGATCCAGCAATGCCGCGTGTGTGAAGAAGGCCTTCGGGTTGTAAAGCACTTTAAGTTGGGAGGAAAAGCTTGGGGTTAATAGCCTTAAGTGTTGACGTTACCAACAGAATAAGCACCGGCTAACTCCGTGCCAGCAGCCGCGGTAATACGGAGGGTGCAAGCGTTAATCGGAATTACTGGGCGTAAAGCGCGCGTAGGCGGTTTGTTAAGTCAGATGTGAAATCCCCGGGCTCAACCTGGGAATGGCATTTGAGACTGGCAGGCTAGAGTATGGTAGAGGTGAGTGGAATTTCAGGTGTAGCGGTGAAATGCGTAGAGATCTGAAGGAACATCAGTGGCGAAGGCGACTCACTGGGCCATTACTGACGCTGAGGTGCGAAAGCGTGGGTAGCAAACAGGATTAGATACCCCAGTAGTC |
| OTU_003278 | NR_074321.1 | 98.92 | Methylophaga nitratireducenticrescens | Piscirickettsiaceae | CCTACAGGCGGCAGCAGTGGGGAATATTGGACAATGGGCGCAAGCCTGATCCAGCAATGCCGCGTGTGTGAAGAAGGCCTTCGGGTTGTAAAGCACTTTAAGTTGGGAGGAAAAGTTTAGGGTTAATAGCCTTAAGTGTTGACGTTACCAACAGAATAAGCACCGGCTAACTCCGTGCCAGCAGCCGCGGTAATACGGAGGGTGCAAGCGTTAATCGGAATTACTGGGCGTAAAGCGCGCGTAGGCGGTTTGTTAAGTCAGATGTGAAATCCCCGGGCTCAACCTGGGAATGGCATTTGAGACTGGCAGGCTAGAGTATGGTAGAGGTGAGTGGAATTTCAGGTGTAGCGGTGAAATGCGTAGAGATCTGAAGGAACATCAGTGGCGAAGGCGACTCACTGGGCCATTACTGACGCTGAGGTGCGAAAGCGTGGGTAGCAAACAGGATTAGATACCCGGGTAGTC |
| OTU_000003 | NR_132662.1 | 99.32 | Marivita roseacus | Rhodobacteraceae | CCTACGGGGGGCTGCAGTGGGGAATCTTAGACAATGGGCGCAAGCCTGATCTAGCCATGCCGCGTGAGTGATGAAGGCCTTAGGGTCGTAAAGCTCTTTCGCCTGTGAAGATAATGACTGTAGCAGGTAAAGAAACCCCGGCTAACTCCGTGCCAGCAGCCGCGGTAATACGGAGGGGGTTAGCGTTGTTCGGAATTACTGGGCGTAAAGCGCGCGTAGGCGGACATTTAAGTCAGAGGTGAAATCCCAGGGCTCAACCCTGGAACTGCCTTTGATACTGGGTGTCTTGAGTTCGAGAGAGGTGAGTGGAATTCCGAGTGTAGAGGTGAAATTCGTAGATATTCGGAGGAACACCAGTGGCGAAGGCGGCTCACTGGCTCGATACTGACGCTGAGGTGCGAAAGTGTGGGGAGCAAACAGGATTAGATACCCCGGTAGTC |
| OTU_000040 | NR_115918.1 | 99.09 | Thalassobacter stenotrophicus | Rhodobacteraceae | CCTACGGGGGGCTGCAGTGGGGAATCTTAGACAATGGGCGCAAGCCTGATCTAGCCATGCCGCGTGAGTGATGAAGGCCCTAGGGTCGTAAAGCTCTTTCGCCAGAGATGATAATGACAGTATCTGGTAAAGAAACCCCGGCTAACTCCGTGCCAGCAGCCGCGGTAATACGGAGGGGGTTAGCGTTGTTCGGAATTACTGGGCGTAAAGCGCGCGTAGGCGGACTATTAAGTTAGAGGTGAAATCCCAGGGCTCAACCCTGGAACTGCCTTTAATACTGGTAGTCTTGAGTTCGAGAGAGGTGAGTGGAATTCCAAGTGTAGAGGTGAAATTCGTAGATATTTGGAGGAACACCAGTGGCGAAGGCGGCTCACTGGCTCGATACTGACGCTGAGGTGCGAAAGTGTGGGGAGCAAACAGGATTAGATACCCCAGTAGTC |
| OTU_000064 | NR_156050.1 | 99.09 | Marimonas arenosa | Rhodobacteraceae | CCTACGGGGGGCTGCAGTGGGGAATCTTAGACAATGGGCGCAAGCCTGATCTAGCCATGCCGCGTGAGTGATGAAGGCCCTAGGGTCGTAAAGCTCTTTCGCCAGGGATGATAATGACAGTACCTGGTAAAGAAACCCCGGCTAACTCCGTGCCAGCAGCCGCGGTAATACGGAGGGGGTTAGCGTTGTTCGGAATTACTGGGCGTAAAGCGCGCGTAGGCGGATTAGTCAGTCAGAGGTGAAATCCCAGGGCTCAACCCTGGAACTGCCTTTGATACTGCTAGTCTTGAGTTCGAGAGAGGTGAGTGGAATTCCGAGTGTAGAGGTGAAATTCGTAGATATTCGGAGGAACACCAGTGGCGAAGGCGGCTCACTGGCTCGATACTGACGCTGAGGTGCGAAAGCGTGGGGAGCAAACAGGATTAGATACCCCGGTAGTC |
| OTU_000081 | NR_153748.1 | 97.73 | Primorskyibacter aestuariivivens | Rhodobacteraceae | CCTACGGGGGGCTGCAGTGGGGAATCTTGGACAATGGGGGCAACCCTGATCCAGCCATGCCGCGTGAGTGATGAAGGCCCTAGGGTCGTAAAGCTCTTTCGCCAGGGATGATAATGACAGTACCTGGTAAAGAAACCCCGGCTAACTCCGTGCCAGCAGCCGCGGTAATACGGAGGGGGTTAGCGTTGTTCGGAATTACTGGGCGTAAAGCGCGCGTAGGCGGACTGGAAAGTTGGGGGTGAAATCCCAGGGCTCAACCCTGGAACTGCCTCCAAAACTATCAGTCTAGAGTTCGAGAGAGGTGAGTGGAATTCCGAGTGTAGAGGTGAAATTCGTAGATATTCGGAGGAACACCAGTGGCGAAGGCGGCTCACTGGCTCGATACTGACGCTGAGGTGCGAAAGTGTGGGGAGCAAACAGGATTAGATACCCCAGTAGTC |
| OTU_000137 | NR_148642.1 | 93.41 | Ahrensia marina | Rhodobacteraceae | CCTACGGGGGGCTGCAGTGGGGAATATTGCACAATGGGCGCAAGCCTGATGCAGCCATGCCGCGTGTGTGATGAAGGCCTTAGGGTTGTAAAACACTTTCATCGGTGAAGATAATGACGGTAGCCGAAGAAGAAGCCCCGGCTAACTCCGTGCCAGCAGCCGCGGTAATACGGAGGGGGCAAGCGTTGTTCGGAATTACTGGGCGTAAAGCGCACGTAGGCGGACTGATCAGTTGGGGGTGAAATCCCGAGGCTTAACCTCGGAACTGCCTTCAATACTGTCAGTCTTGAGATCGGAAGAGGTGAGTGGAATTCCTAGTGTAGAGGTGAAATTCGTAGATATTAGGAAGAACACCAGTGGCGAAGGCGGCTCACTGGTCCGATACTGACGCTGAGGTGCGAAAGCGTGGGGAGCGAACAGGATTAGATACCCCGGTAGTC |
| OTU_000298 | NR_145564.1 | 98.86 | Aliiroseovarius zhejiangensis | Rhodobacteraceae | CCTACGGGTGGCAGCAGTGGGGAATCTTAGACAATGGGCGCAAGCCTGATCTAGCCATGCCGCGTGAGTGATGAAGGCCTTAGGGTCGTAAAGCTCTTTCACCAGGGATGATAATGACAGTACCTGGAAAAGAAACCCCGGCTAACTCCGTGCCAGCAGCCGCGGTAATACGGAGGGGGTTAGCGTTGTTCGGAATTACTGGGCGTAAAGCGCGCGTAGGCGGATTAGTCAGTCAGAGGTGAAATCCCAGGGCTCAACCCTGGAACTGCCTTTGATACTGCTAGTCTTGAGTTCGAGAGAGGTGAGTGGAATTCCGAGTGTAGAGGTGAAATTCGTAGATATTCGGAGGAACACCAGTGGCGAAGGCGGCTCACTGGCTCGATACTGACGCTGAGGTGCGAAAGCGTGGGGAGCAAACAGGATTAGATACCCGAGTAGTC |
| OTU_000422 | NR_132662.1 | 99.32 | Marivita roseacus | Rhodobacteraceae | CCTACGGGTGGCAGCAGTGAGGAATATTGGACAATGGGCGCAAGCCTGATCTAGCCATGCCGCGTGAGTGATGAAGGCCTTAGGGTCGTAAAGCTCTTTCGCCTGTGAAGATAATGACTGTAGCAGGTAAAGAAACCCCGGCTAACTCCGTGCCAGCAGCCGCGGTAATACGGAGGGGGTTAGCGTTGTTCGGAATTACTGGGCGTAAAGCGCGCGTAGGCGGACATTTAAGTCAGAGGTGAAATCCCAGGGCTCAACCCTGGAACTGCCTTTGATACTGGGTGTCTTGAGTTCGAGAGAGGTGAGTGGAATTCCGAGTGTAGAGGTGAAATTCGTAGATATTCGGAGGAACACCAGTGGCGAAGGCGGCTCACTGGCTCGATACTGACGCTGAGGTGCGAAAGTGTGGGGAGCAAACAGGATTAGATACCCGTGTAGTC |
| OTU_000440 | NR_132662.1 | 99.32 | Marivita roseacus | Rhodobacteraceae | CCTACGGGTGGCAGCAGTGGGGAATCTTAGACAATGGGCGCAAGCCTGATCTAGCCATGCCGCGTGAGTGATGAAGGCCTTAGGGTCGTAAAGCTCTTTCGCCTGTGAAGATAATGACTGTAGCAGGTAAAGAAACCCCGGCTAACTCCGTGCCAGCAGCCGCGGTAATACGGAGGGGGTTAGCGTTGTTCGGAATTACTGGGCGTAAAGCGCGCGTAGGCGGACATTTAAGTCAGAGGTGAAATCCCAGGGCTCAACCCTGGAACTGCCTTTGATACTGGGTGTCTTGAGTTCGAGAGAGGTGAGTGGAATTCCGAGTGTAGAGGTGAAATTCGTAGATATTCGGAAGAACACCAGTGGCGAAGGCGGCTCACTGGCTCGGTACTGACGCTGAGGTGCGAAAGCGTGGGGAGCAAACAGGATTAGATACCCTTGTAGTC |
| OTU_000576 | NR_116682.1 | 98.86 | Marivita byunsanensis | Rhodobacteraceae | CCTACGGGGGGCAGCAGTGGGGAATCTTAGACAATGGGCGCAAGCCTGATCTAGCCATGCCGCGTGAGTGATGAAGGCCTTAGGGTCGTAAAGCTCTTTCGCCTGTGAAGATAATGACTGTAGCAGGTAAAGAAACCCCGGCTAACTCCGTGCCAGCAGCCGCGGTAATACGGAGGGGGTTAGCGTTGTTCGGAATTACTGGGCGTAAAGCGCGCGTAGGCGGATTAGTCAGTCAGAGGTGAAATCCCAGGGCTCAACCCTGGAACTGCCTTTGATACTGCTAGTCTTGAGTTCGAGAGAGGTGAGTGGAATTCCGAGTGTAGAGGTGAAATTCGTAGATATTCGGAGGAACACCAGTGGCGAAGGCGGCTCACTGGCTCGATACTGACGCTGAGGTGCGAAAGTGTGGGGAGCAAACAGGATTAGATACCCTGGTAGTC |
| OTU_000628 | NR_148278.1 | 98.41 | Antarctobacter jejuensis | Rhodobacteraceae | CCTACGGGGGGCTGCAGTGGGGAATCTTAGACAATGGGCGCAAGCCTGATCTAGCCATGCCGCGTGTGTGATGAAGGCCCTAGGGTCGTAAAGCACTTTCGCCAGGGATGATAATGACAGTACCTGGTAAAGAAACCCCGGCTAACTCCGTGCCAGCAGCCGCGGTAATACGGAGGGGGTTAGCGTTGTTCGGAATTACTGGGCGTAAAGCGCACGTAGGCGGATCAGAAAGTAGGGGGTGAAATCCCGGGGCTCAACCCCGGAACTGCCTTCTAAACTCCTGGTCTAGAGTTCGAGAGAGGTGAGTGGAATTCCGAGTGTAGAGGTGAAATTCGTAGATATTCGGAGGAACACCAGTGGCGAAGGCGGCTCACTGGCTCGATACTGACGCTGAGGTGCGAAAGTGTGGGGAGCAAACAGGATTAGATACCCTGGTAGTC |
| OTU_000631 | NR_115918.1 | 98.64 | Thalassobacter stenotrophicus | Rhodobacteraceae | CCTACGGGTGGCAGCAGTGGGGAATCTTAGACAATGGGCGCAAGCCTGATCTAGCCATGCCGCGTGAGTGATGAAGGCCCTAGGGTCGTAAAGCTCTTTCGCCAGAGATGATAATGACAGTATCTGGTAAAGAAACCCCGGCTAACTCCGTGCCAGCAGCCGCGGTAATACGGAGGGGGTTAGCGTTGTTCGGAATTACTGGGCGTAAAGCGCGCGTAGGCGGACTATTAAGTTAGAGGTGAAATCCCAGGGCTCAACCCTGGAACTGCCTTTAATACTGGGTGTCTTGAGTTCGAGAGAGGTGAGTGGAATTCCGAGTGTAGAGGTGAAATTCGTAGATATTCGGAGGAACACCAGTGGCGAAGGCGGCTCACTGGCTCGATACTGACGCTGAGGTGCGAAAGTGTGGGGAGCAAACAGGATTAGATACCCTAGTAGTC |
| OTU_000711 | NR_132662.1 | 97.95 | Marivita roseacus | Rhodobacteraceae | CCTACGGGAGGCAGCAGTGAGGAATATTGGACAATGGAGGCAACTCTGATCCAGCCATGCCGCGTGAGTGATGAAGGCCTTAGGGTCGTAAAGCTCTTTCGCCTGTGAAGATAATGACTGTAGCAGGTAAAGAAACCCCGGCTAACTCCGTGCCAGCAGCCGCGGTAATACGGAGGGGGTTAGCGTTGTTCGGAATTACTGGGCGTAAAGCGCGCGTAGGCGGACATTTAAGTCAGAGGTGAAATCCCAGGGCTCAACCCTGGAACTGCCTTTGATACTGGGTGTCTTGAGTTCGAGAGAGGTGAGTGGAATTCCGAGTGTAGAGGTGAAATTCGTAGATATTCGGAGGAACACCAGTGGCGAAGGCGGCTCACTGGCTCGATACTGACGCTGAGGTGCGAAAGTGTGGGGAGCAAACAGGATTAGATACCCCGGTAGTC |
| OTU_000752 | NR_132662.1 | 97.73 | Marivita roseacus | Rhodobacteraceae | CCTACGGGGGGCAGCAGTGGGGAATATTGCACAATGGGGGAAACCCTGATGCAGCCATGCCGCGTGAGTGATGAAGGCCTTAGGGTCGTAAAGCTCTTTCGCCTGTGAAGATAATGACTGTAGCAGGTAAAGAAACCCCGGCTAACTCCGTGCCAGCAGCCGCGGTAATACGGAGGGGGTTAGCGTTGTTCGGAATTACTGGGCGTAAAGCGCGCGTAGGCGGACATTTAAGTCAGAGGTGAAATCCCAGGGCTCAACCCTGGAACTGCCTTTGATACTGGGTGTCTTGAGTTCGAGAGAGGTGAGTGGAATTCCGAGTGTAGAGGTGAAATTCGTAGATATTCGGAGGAACACCAGTGGCGAAGGCGGCTCACTGGCTCGATACTGACGCTGAGGTGCGAAAGTGTGGGGAGCAAACAGGATTAGATACCCGGGTAGTC |
| OTU_000773 | NR_132662.1 | 97.95 | Marivita roseacus | Rhodobacteraceae | CCTACGGGCGGCAGCAGTGGGGAATCTTAGACAATGGGCGCAAGCCTGATCTAGCCATGCCGCGTGAGTGATGAAGGCCCTAGGGTCGTAAAGCTCTTTCGCCAGGGATGATAATGACAGTACCTGGTAAAGAAACCCCGGCTAACTCCGTGCCAGCAGCCGCGGTAATACGGAGGGGGTTAGCGTTGTTCGGAATTACTGGGCGTAAAGCGCGCGTAGGCGGACATTTAAGTCAGAGGTGAAATCCCAGGGCTCAACCCTGGAACTGCCTTTGATACTGGGTGTCTTGAGTTCGAGAGAGGTGAGTGGAATTCCGAGTGTAGAGGTGAAATTCGTAGATATTCGGAGGAACACCAGTGGCGAAGGCGGCTCACTGGCTCGATACTGACGCTGAGGTGCGAAAGTGTGGGGAGCAAACAGGATTAGATACCCTAGTAGTC |
| OTU_000776 | NR_115918.1 | 99.09 | Thalassobacter stenotrophicus | Rhodobacteraceae | CCTACGGGAGGCAGCAGTGGGGAATCTTGGACAATGGGCGCAAGCCTGATCCAGCCATGCCGCGTGAGTGATGAAGGCCTTAGGGTCGTAAAGCTCTTTCGCCAGAGATGATAATGACAGTATCTGGTAAAGAAACCCCGGCTAACTCCGTGCCAGCAGCCGCGGTAATACGGAGGGGGTTAGCGTTGTTCGGAATTACTGGGCGTAAAGCGCGCGTAGGCGGACTATTAAGTTAGAGGTGAAATCCCAGGGCTCAACCCTGGAACTGCCTTTAATACTGGTAGTCTTGAGTTCGAGAGAGGTGAGTGGAATTCCAAGTGTAGAGGTGAAATTCGTAGATATTTGGAGGAACACCAGTGGCGAAGGCGGCTCACTGGCTCGATACTGACGCTGAGGTGCGAAAGTGTGGGGAGCAAACAGGATTAGATACCCGGGTAGTC |
| OTU_001301 | NR_132662.1 | 98.64 | Marivita roseacus | Rhodobacteraceae | CCTACGGGAGGCTGCAGTGGGGAATCTTAGACAATGGGCGCAAGCCTGATCTAGCCATGCCGCGTGAGTGATGAAGGCCTTAGGGTCGTAAAGCTCTTTCGCCTGTGAAGATAATGACTGTAGCAGGTAAAGAAGCCCCGGCTAACTTCGTGCCAGCAGCCGCGGTAATACGAAGGGGGCTAGCGTTGTTCGGAATTACTGGGCGTAAAGCGCGCGTAGGCGGACATTTAAGTCAGAGGTGAAATCCCAGGGCTCAACCCTGGAACTGCCTTTGATACTGGGTGTCTTGAGTTCGAGAGAGGTGAGTGGAATTCCGAGTGTAGAGGTGAAATTCGTAGATATTCGGAGGAACACCAGTGGCGAAGGCGGCTCACTGGCTCGATACTGACGCTGAGGTGCGAAAGTGTGGGGAGCAAACAGGATTAGATACCCTAGTAGTC |
| OTU_001324 | NR_132662.1 | 98.41 | Marivita roseacus | Rhodobacteraceae | CCTACGGGGGGCAGCAGTGGGGAATCTTAGACAATGGGCGCAAGCCTGATCTAGCCATGCCGCGTGAGTGATGAAGGCCTTAGGGTCGTAAAGCTCTTTCGCCTGTGAAGATAATGACTGTAGCAGGTAAAGAAACCCCGGCTAACTCCGTGCCAGCAGCCGCGGTAATACGGAGGGGGTTAGCGTTGTTCGGAATTACTGGGCGTAAAGCGCGCGTAGGCGGACATTTAAGTCAGAGGTGAAATCCCAGGGCTCAACCCTGGAACTGCCTTTGATACTGGGTGTCTTGAGTTCGAGAGAGGTGAGTGGAATTCCGAGTGTAGAGGTGAAATTCGTAGATATTCGGAGGAACACCAGTGGCGAAGGCGGCTCACTGGCTCGATACTGACGCTGAGGGACGAAAGCGTGGGGAGCGAACAGGATTAGATACCCGAGTAGTC |
| OTU_001405 | NR_132662.1 | 98.41 | Marivita roseacus | Rhodobacteraceae | CCTACGGGTGGCTGCAGTGGGGAATCTTAGACAATGGGCGCAAGCCTGATCTAGCCATGCCGCGTGAGTGATGAAGGCCTTAGGGTCGTAAAGCTCTTTCGCCTGTGAAGATAATGACTGTAGCAGGTAAAGAAACCCCGGCTAACTCCGTGCCAGCAGCCGCGGTAATACGGAGGGGGTTAGCGTTGTTCGGAATTACTGGGCGTAAAGCGCGCGTAGGCGGACATTTAAGTCAGAGGTGAAATCCCAGGGCTCAACCCTGGAACTGCCTTTGATACTGGGTGTCTTGAGTTCGAGAGAGGTGAGTGGAATTCCGAGTGTAGAGGTGAAATTCGTAGATATTCGGAGGAACACCAGTGGCGAAGGCGGCTCACTGGCTCGATACTGACGCTCATGTGCGAAAGTGTGGGTAGCGAACAGGATTAGATACCCTTGTAGTC |
| OTU_002024 | NR_109594.1 | 98.86 | Roseovarius litoreus | Rhodobacteraceae | CCTACGGGCGGCAGCAGTGGGGAATCTTGGACAATGGGGGCAACCCTGATCCAGCCATGCCGCGTGAGTGATGAAGGCCCTAGGGTCGTAAAGCTCTTTCGCCAGGGATGATAATGACAGTACCTGGTAAAGAAACCCCGGCTAACTCCGTGCCAGCAGCCGCGGTAATACGGAGGGGGTTAGCGTTGTTCGGAATTACTGGGCGTAAAGCGCGCGTAGGCGGATTGGAAAGTTGGGGGTGAAATCCCAGGGCTCAACCCTGGAACGGCCTCCAAAACTCCCAGTCTTGAGTTCGAGAGAGGTGAGTGGAATTCCGAGTGTAGAGGTGAAATTCGTAGATATTCGGAGGAACACCAGTGGCGAAGGCGGCTCACTGGCTCGATACTGACGCTGAGGTGCGAAAGTGTGGGGAGCAAACAGGATTAGATACCCGGGTAGTC |
| OTU_002076 | NR_136489.1 | 97.95 | Mameliella atlantica | Rhodobacteraceae | CCTACGGGTGGCTGCAGTGGGGAATCTTAGACAATGGGGGCAACCCTGATCTAGCCATGCCGCGTGAGTGATGAAGGCCCTAGGGTCGTAAAGCTCTTTCGCCAGGGATGATAATGACAGTACCTGGTAAAGAAACCCCGGCTAACTCCGTGCCAGCAGCCGCGGTAATACGGAGGGGGTTAGCGTTGTTCGGAATTACTGGGCGTAAAGCGCGCGTAGGCGGATTGGAAAGTTGGGGGTGAAATCCCGGGGCTCAACCCCGGAACGGCCTCCAAAACTCCCAGTCTTGAGTTCGAGAGAGGTGAGTGGAATTCCGAGTGTAGAGGTGAAATTCGTAGATATTCGGAGGAACACCAGTGGCGAAGGCGGCTCACTGGCTCGATACTGACGCTGAGGTGCGAAAGTGTGGGGAGCAAACAGGATTAGATACCCGTGTAGTC |
| OTU_003560 | NR_132662.1 | 98.41 | Marivita roseacus | Rhodobacteraceae | CCTACGGGTGGCTGCAGTCGGGAATATTGGACAATGGGGGCAACCCTGATCTAGCCATGCCGCGTGAGTGATGAAGGCCTTAGGGTCGTAAAGCTCTTTCGCCTGTGAAGATAATGACTGTAGCAGGTAAAGAAACCCCGGCTAACTCCGTGCCAGCAGCCGCGGTAATACGGAGGGGGTTAGCGTTGTTCGGAATTACTGGGCGTAAAGCGCGCGTAGGCGGACATTTAAGTCAGAGGTGAAATCCCAGGGCTCAACCCTGGAACTGCCTTTGATACTGGGTGTCTTGAGTTCGAGAGAGGTGAGTGGAATTCCGAGTGTAGAGGTGAAATTCGTAGATATTCGGAGGAACACCAGTGGCGAAGGCGGCTCACTGGCTCGATACTGACGCTGAGGTGCGAAAGTGTGGGGAGCAAACAGGATTAGATACCCTGGTAGTC |
| OTU_000035 | NR_147733.1 | 98.86 | Pyruvatibacter mobilis | Rhodobiaceae | CCTACGGGGGGCTGCAGTGGGGAATATTGGACAATGGGCGCAAGCCTGATCCAGCCATGCCGCGTGAGTGATGAAGGCCCTAGGGTTGTAAAACTCTTTCAGTGGTGAAGATAATGACGGTAACCACAGAAGAAGCTCCGGCTAACTCCGTGCCAGCAGCCGCGGTAATACGGAGGGAGCTAGCGTTGTTCGGAATTACTGGGCGTAAAGCGCACGTAGGCGGTCTATAAAGTTGGGGGTGAAATCCCGGAGCTCAACTCCGGAACTGCCTCCAAAACTGCTAGACTCGAGTTCGGAAGAGGTAAGTAGAATTCCCAGTGTAGAGGTGAAATTCGTAGATATTGGGAAGAATACCAGTGGCGAAGGCGGCTTACTGGTCCGATACTGACGCTGAGGTGCGAAAGCGTGGGGAGCAAACAGGATTAGATACCCCGGTAGTC |
| OTU_000190 | NR_117452.1 | 92.08 | Tepidamorphus gemmatus | Rhodobiaceae | CCTACGGGGGGCTGCAGTGGGGAATATTGGACAATGGGCGCAAGCCTGATCCAGCCATGCCGCGTGAGTGAAGAAGGCCTTAGGGTTGTAAAGCTCTTTCAGCAGGGAAGATGATGACGGTACCTGCAGAAGAAGCCCCGGCCAACTCCGTGCCAGCAGCCGCGGTAATACGGAGGGGGCAAGCGTTGTTCGGAATTACTGGGCGTAAAGCGCGCGTAGGCGGTTTGTCTAGTCAGGCGTGAAATCCCGGGGCTCAACCCCGGAACTGCGCTTGATACTGGCATACTAGAGGTCTGGAGAGGGTAGTGGAATTCCCAGTGTAGAGGTGAAATTCGTAGATATTGGGAAGAACACCAGCGGCGAAGGCGGCTACCTGGACAGATACTGACGCTGAGGTGCGAAAGCGTGGGGAGCAAACAGGATTAGATACCCCAGTAGTC |
| OTU_001321 | NR_147733.1 | 98.64 | Pyruvatibacter mobilis | Rhodobiaceae | CCTACGGGAGGCAGCAGTGGGGAATATTGGACAATGGGCGCAAGCCTGATCCAGCCATGCCGCGTGAGTGATGAAGGCCCTAGGGTTGTAAAACTCTTTCAGTGGTGAAGATAATGACGGTAACCACAGAAGAAGCTCCGGCTAACTCCGTGCCAGCAGCCGCGGTAATACGGAGGGAGCTAGCGTTGTTCGGAATTACTGGGCGTAAAGCGCACGTAGGCGGTCTATAAAGTTGGGGGTGAAATCCCGGAGCTCAACTCCGGAACTGCCTCCAAAACTGCTAGACTCGAGTTCGGAAGAGGTAAGTAGAATTCCCAGTGTAGAGGTGAAATTCGTAGATATTGGGAAGAACACCAGTGGCGAAGGCGGCTCACTGGTCCGTAACTGACGCTGAGGTGCGAAAGCGTGGGGAGCAAACAGGATTAGATACCCTGGTAGTC |
| OTU_002737 | NR_147733.1 | 98.64 | Pyruvatibacter mobilis | Rhodobiaceae | CCTACGGGAGGCAGCAGTGGGGAATCTTAGACAATGGGCGCAAGCCTGATCTAGCCATGCCGCGTGAGTGATGAAGGCCCTAGGGTTGTAAAACTCTTTCAGTGGTGAAGATAATGACGGTAACCACAGAAGAAGCTCCGGCTAACTCCGTGCCAGCAGCCGCGGTAATACGGAGGGAGCTAGCGTTGTTCGGAATTACTGGGCGTAAAGCGCACGTAGGCGGTCTATAAAGTTGGGGGTGAAATCCCGGAGCTCAACTCCGGAACTGCCTCCAAAACTGCTAGACTCGAGTTCGGAAGAGGTAAGTAGAATTCCCAGTGTAGAGGTGAAATTCGTAGATATTGGGAAGAATACCAGTGGCGAAGGCGGCTTACTGGTCCGATACTGACGCTGAGGTGCGAAAGCGTGGGGAGCAAACAGGATTAGATACCCTTGTAGTC |
| OTU_003215 | NR_147733.1 | 97.50 | Pyruvatibacter mobilis | Rhodobiaceae | CCTACGGGGGGCTGCAGTGGGGAATATTGGACAATGGGCGCAAGCCTGATCCAGCCATGCCGCGTGAGTGATGAAGGCCCTAGGGTTGTAAAACTCTTTCAGTGGTGAAGATAATGACGGTACCTGCAGAAGAAGCCCCGGCTAACTCCGTGCCAGCAGCCGCGGTAATACGGAGGGGGCTAGCGTTGTTCGGAATTACTGGGCGTAAAGCGCACGTAGGCGGTCTATAAAGTTGGGGGTGAAATCCCGGAGCTCAACTCCGGAACTGCCTCCAAAACTGCTAGACTCGAGTTCGGAAGAGGTAAGTAGAATTCCCAGTGTAGAGGTGAAATTCGTAGATATTGGGAAGAATACCAGTGGCGAAGGCGGCTTACTGGTCCGATACTGACGCTGAGGTGCGAAAGCGTGGGGAGCAAACAGGATTAGATACCCGAGTAGTC |
| OTU_000076 | NR_116475.1 | 92.78 | Magnetospira thiophila | Rhodospirillaceae | CCTACGGGGGGCTGCAGTGGGGAATATTGGACAATGGGGGAAACCCTGATCCAGCAATGCCGCGTGAGTGAAGAAGGCCTTAGGGTTGTAAAACTCTTTCACTGATGAAGATGATGACGGTAATCAGAGAAGAAGCCCCGGCTAACTCCGTGCCAGCAGCCGCGGTAATACGGAGGGGGCTAGCGTTATTCGGATTTACTGGGCGTAAAGCGCACGTAGGCGGCGTATCTAGTCAGAGGTGAAAGCCCGGGGCTCAACCCCGGAATTGCCTTTGATACTGGTATGCTTGAGTTCGGAAGAGGGTAGTGGAATTCCAAGTGTAGAGGTGAAATTCGTAGATATTTGGAAGAACACCGGTGGCGAAGGCGGCTACCTGGTCCGATACTGACGCTGAGGTGCGAAAGCGTGGGGAGCGAACAGGATTAGATACCCCGGTAGTC |
| OTU_000077 | NR_134169.1 | 91.40 | Salinispirillum marinum | Saccharospirillaceae | CCTACGGGGGGCTGCAGTCGGGAATATTGGACAATGGGGGCAACCCTGATCCAGCCATGCCGCGTGTGTGAAGAAGGCCCTAGGGTTGTAAAGCACTTTAAGTTGTGAAGAAGGCTGTACGGTTAATAGCCGTGCGGATTGACATTAGCAACAGAATAAGCACCGGCTAACTCCGTGCCAGCAGCCGCGGTAATACGGAGGGTGCAAGCGTTAATCGGAATTACTGGGCGTAAAGCGCGCGTAGGTGGTTTGTTAAGTGAGATGTGAAAGCCCAGGGCTCAACCTTGGAACTGCATCTCATACTGGCAGGCTAGAGTATGGTAGAGGGAGGTAGAATTCCACGTGTAGCGGTGAAATGCGTAGAGATGTGGAGGAATACCAGTGGCGAAGGCGGCCTCCTGGACTAATACTGACACTGAGGTGCGAAAGCGTGGGGAGCAAACAGGATTAGATACCCCAGTAGTC |
| OTU_000084 | NR_025814.1 | 99.09 | Sphingopyxis flavimaris | Sphingomonadaceae | CCTACGGGGGGCTGCAGTGGGGAATATTGGACAATGGGCGAAAGCCTGATCCAGCAATGCCGCGTGAGTGATGAAGGCCTTAGGGTTGTAAAGCTCTTTTACCAGGGATGATAATGACAGTACCTGGAGAATAAGCTCCGGCTAACTCCGTGCCAGCAGCCGCGGTAATACGGAGGGAGCTAGCGTTGTTCGGAATTACTGGGCGTAAAGCGCGCGTAGGCGGTTACTCAAGTCAGAGGTGAAAGCCCGGAGCTCAACTCCGGAACTGCCTTTGAAACTAGGTGACTAGAATCTTGGAGAGGCGAGTGGAATTCCGAGTGTAGAGGTGAAATTCGTAGATATTCGGAAGAACACCAGTGGCGAAGGCGACTCGCTGGACAAGTATTGACGCTGAGGTGCGAAAGCGTGGGGAGCAAACAGGATTAGATACCCCAGTAGTC |
| OTU_000086 | NR_118015.1 | 95.70 | Spongiibacter marinus | Spongiibacteraceae | CCTACGGGGGGCTGCAGTGGGGAATATTGCGCAATGGGGGAAACCCTGACGCAGCCATGCCGCGTGTGTGAAGAAGGCTTTCGGGTTGTAAAGCACTTTCAATAGGGAGGAAAGGTTGCAGATTAATACTCTGTAGCTGTGACGTTACCTATAGAAGAAGCACCGGCTAACTCCGTGCCAGCAGCCGCGGTAATACGGAGGGTGCAAGCGTTAATCGGAATTACTGGGCGTAAAGCGCGCGTAGGCGGCTTCGTCAGTTGGATGTGAAAGCCCCGGGCTTAACCTGGGAACTGCATTCAATACTGCGGAGCTAGAGTATGGTAGAGGATAGTGGAATTCCAGGTGTAGCGGTGAAATGCGTAGATATCTGGAGGAACATCAGTGGCGAAGGCGACTGTCTGGACCAATACTGACGCTGAGGTGCGAAAGCGTGGGGAGCAAACAGGATTAGATACCCCAGTAGTC |

Table S3B - Core microbiome (52 OTUs) of *Alexandrium minutum/tamarense*, specifying: OTU, accession number of closest relative in GenBank, % identity, genus and family of that strain and the sequence of the OTU.
